# Understanding the key features of the spontaneous formation of *bona fide* prions through a novel methodology that enables their swift and consistent generation

**DOI:** 10.1186/s40478-023-01640-8

**Published:** 2023-09-07

**Authors:** Hasier Eraña, Carlos M. Díaz-Domínguez, Jorge M. Charco, Enric Vidal, Ezequiel González-Miranda, Miguel A. Pérez-Castro, Patricia Piñeiro, Rafael López-Moreno, Cristina Sampedro-Torres-Quevedo, Leire Fernández-Veiga, Juan Tasis-Galarza, Nuria L. Lorenzo, Aileen Santini-Santiago, Melisa Lázaro, Sandra García-Martínez, Nuno Gonçalves-Anjo, Maitena San-Juan-Ansoleaga, Josu Galarza-Ahumada, Eva Fernández-Muñoz, Samanta Giler, Mikel Valle, Glenn C. Telling, Mariví Geijó, Jesús R. Requena, Joaquín Castilla

**Affiliations:** 1 Center for Cooperative Research in Biosciences (CIC bioGUNE), Basque Research and Technology Alliance (BRTA), Bizkaia Technology Park, 48160 Derio, Bizkaia Spain; 2 ATLAS Molecular Pharma S. L. Bizkaia Technology Park, 48160 Derio, Spain; 3 Centro de Investigación Biomédica en Red de Enfermedades Infecciosas (CIBERINFEC), Carlos III National Health Institute, 28029 Madrid, Spain; 4 IRTA, Programa de Sanitat Animal, Centre de Recerca en Sanitat Animal (CReSA), Campus de la Universitat Autònoma de Barcelona (UAB), Bellaterra, Catalonia Spain; 5 CIMUS Biomedical Research Institute and Department of Medical Sciences, University of Santiago de Compostela-IDIS, 15782 Santiago de Compostela, Spain; 6 Prion Research Center (PRC), Colorado State University, Fort Collins, CO 80523 USA; 7 Animal Health Department, NEIKER-Basque Institute for Agricultural Research and Development, Basque Research and Technology Alliance (BRTA), Bizkaia Technology Park, 48160 Derio, Spain; 8 IKERBASQUE, Basque Foundation for Science, 48011 Bilbao, Spain

**Keywords:** Prion, Transmissible spongiform encephalopathies, Spontaneous misfolding, Strain

## Abstract

Among transmissible spongiform encephalopathies or prion diseases affecting humans, sporadic forms such as sporadic Creutzfeldt–Jakob disease are the vast majority. Unlike genetic or acquired forms of the disease, these idiopathic forms occur seemingly due to a random event of spontaneous misfolding of the cellular PrP (PrP^C^) into the pathogenic isoform (PrP^Sc^). Currently, the molecular mechanisms that trigger and drive this event, which occurs in approximately one individual per million each year, remain completely unknown. Modelling this phenomenon in experimental settings is highly challenging due to its sporadic and rare occurrence. Previous attempts to model spontaneous prion misfolding in vitro have not been fully successful, as the spontaneous formation of prions is infrequent and stochastic, hindering the systematic study of the phenomenon. In this study, we present the first method that consistently induces spontaneous misfolding of recombinant PrP into *bona fide* prions within hours, providing unprecedented possibilities to investigate the mechanisms underlying sporadic prionopathies. By fine-tuning the Protein Misfolding Shaking Amplification method, which was initially developed to propagate recombinant prions, we have created a methodology that consistently produces spontaneously misfolded recombinant prions in 100% of the cases. Furthermore, this method gives rise to distinct strains and reveals the critical influence of charged surfaces in this process.

**Supplementary Information:**

The online version contains supplementary material available at 10.1186/s40478-023-01640-8.

## Introduction

Sporadic prion disease in humans, in particular sporadic Creutzfeldt–Jakob disease (sCJD), accounts for approximately 85% of currently diagnosed transmissible spongiform encephalopathies (TSE) [27]. With the near eradication of TSE acquired from exogenous sources [72], and the foreseeable reduction of genetic cases resulting from the popularization of genetic counseling and pre-implantation genetic diagnosis [37, 68], sporadic and idiopathic (putatively spontaneous) cases will likely become the focus on the field of these devastating neurodegenerative disorders. However, given the low incidence of sporadic prion disorders in both, humans [27] and other mammals [42], researchers studying the phenomenon of spontaneous misfolding of wild-type prion protein (PrP), probably a stochastic event, suffer from scarcity of suitable models. In fact, the main event underlying genetic and sporadic prion disorders—the conversion of the cellular prion protein (PrP^C^) into a self-propagating and neurotoxic misfolded isoform (PrP^Sc^)—remains a mystery [58]. Despite some theoretical models have been proposed for PrP^Sc^-induced misfolding of PrP^C^, the molecular mechanisms driving the formation of the initial PrP^Sc^ seed are completely unknown, regardless of the presence or absence of disease-associated mutations, which seemingly increase the propensity of PrP^C^ to misfold spontaneously into PrP^Sc^ [3].

To study this event and demonstrate the “protein-only” hypothesis, which states that TSEs are caused by an exclusively proteic pathogen [57], the generation of infectious prions in vitro that mimic the prion misfolding event in a cell-free environment has been pivotal. However, consistently replicating the spontaneous prion formation phenomenon is still challenging. Initially, to demonstrate that PrP^Sc^ was formed from PrP^C^ through a nucleation-dependent autocatalytic process, PrP^Sc^-seeded amplification reactions were attempted. These reactions showed low conversion yields [14, 46], but demonstrated strain-specific propagation and replication of interspecies transmission barriers [8, 47]. Conversion efficiency was later improved by using whole brain homogenates as PrP^C^ source and by incorporating cycles of incubation and sonication. This was key for the development of Protein Misfolding Cyclic Amplification (PMCA) [61]. This enhanced system allowed for the definitive demonstration of the nucleation-dependent prion propagation through indefinite serially diluted amplification reactions. PMCA also revealed the importance of yet unknown brain components, later identified as conversion cofactors that were mainly polyanionic molecules promoting conversion [24, 36], and physical processes such as sonication on the prion propagation or misfolding efficiency. Other modifications, such as the addition of beads [32, 38], further improved conversion efficiency. Such an efficient cell-free prion propagation system led to the observation that protease-resistant PrP (PrP^res^) could be formed in absence of brain-derived PrP^Sc^ at low frequency in PMCA, either with minimal components that seem to stimulate spontaneous conversion [23], or with whole brain homogenates, with few modifications in operational parameters [5]. These de novo generated PrP^res^ turned out to be infectious in vivo and showed unique strain properties, establishing the first in vitro prion misfolding systems that could model sporadic prion disorders. Despite the fact that spontaneous misfolding in vitro could be replicated with PrP^C^ from species other than rodents [52], the formation of prions was always an infrequent and stochastic event.

In order to develop a simpler and more versatile in vitro prion propagation system, which could definitively demonstrate the “protein only” hypothesis and facilitate the study of prion misfolding, the use of recombinant PrP (rec-PrP) as substrate was explored. The first report of the spontaneous generation of a synthetic prion in vitro, capable of causing disease in animal models upon intracerebral inoculation, opened the way for the systematic study of this phenomenon [49]. Although this first recombinant prion preparation was not very efficient in inducing misfolding of brain-derived PrP^C^ and subsequent disease, several different attempts followed [21–23, 44, 50], each providing valuable information regarding the complex mechanism driving PrP conversion into its pathogenic counterpart. In 2010, Wang and colleagues achieved a major breakthrough in this regard. They reported the generation of the first highly infectious recombinant prion spontaneously, in the absence of any mammalian brain-derived material, with incubation periods similar to those of naturally occurring prions in wild-type animals [71]. Apart from giving definitive support to the “protein only” hypothesis [63], this groundbreaking study demonstrated that infectious recombinant prions with high titer could be generated in vitro spontaneously, an invaluable model to study sporadic TSEs. However, in spite of the conclusive results, the method used for the spontaneous generation of prions still required quite complex equipment and a highly specific skillset, as it relied on PMCA. As with the brain homogenate-based system, the difficulty in controlling parameters and the stochastic nature of spontaneous prion protein misfolding made the process difficult to reproduce, as evidenced by unsuccessful attempts by other laboratories [67, 78].

Thus, despite advances in the generation of recombinant prions in vitro using different procedures and reaction substrates, the molecular mechanisms underlying spontaneous PrP misfolding remain largely unknown. Denaturing agents have been used to favor the formation of recombinant PrP^res^ in vitro, although the resulting amyloids exhibit highly variable properties in terms of in vivo infectivity [21, 49, 50]. With regards to substrate composition, a significant advancement in mimicking spontaneous prion misfolding in vitro comes from the removal of brain homogenates or other brain-derived components from the substrates, which are replaced by chemically defined cofactors. These could help reduce the inherent variability of the process, promote the spontaneous misfolding of the recombinant prion protein into infectious *bona fide* prions, and unveil some of the conditions and molecular factors required for it [24, 25, 33, 71, 75, 75]. However, the exact role of these polyanionic cofactors remains unclear. Apart from favoring prion propagation in vitro [23, 71], they have been proposed to restrict or direct prion formation towards cofactor-specific conformers or strains [26, 33]. Furthermore, they have also been deemed necessary for the formation of infectious prions from wild-type PrP [10], although for some disease-associated mutant PrP was proven non-essential, as they could be converted in the absence of cofactors [18, 55].

Inspired by the process that led from seeded, brain homogenate-based PMCA to unseeded, recombinant PrP and chemically defined substrate-based PMCA, we have successfully devised a method that consistently generates highly infectious recombinant prions spontaneously in a simple, rapid and efficient way. Through adaptations in substrate composition, bead type and amount and shaking conditions, we have refined the PMSA methodology [30, 31] to induce the spontaneous formation of highly infectious recombinant prions in less than 24 h, exhibiting expected strain variability. Thus, starting from a method highly efficient for recombinant prion propagation from previously generated seeds, we developed a variant that permits de novo prion formation without the need of seeding with pre-formed prions, being the first method for consistent and swift spontaneous prion misfolding in vitro. The simplicity of this system permits an in depth analysis of each variable that influences the process, bringing us closer to understanding the physicochemical parameters that govern the spontaneous formation of prions. Furthermore, its ease of implementation allows for seamless transfer to any laboratory, providing an invaluable tool for answering all the unknowns underlying the development of sporadic TSEs.

In summary, we present a simple and robust methodology that allows to break down the spontaneous prion misfolding phenomenon into its minimal requirements.

## Material and methods

### Production and purification of recombinant PrP

Bacterial expression and purification of bank vole I109 recombinant PrP (rec-PrP) (amino acids 23-231, based on Genbank accession number AF367624 sequence) was performed as described previously [34]. Briefly, pOPIN E expression vector containing the wild-type I109 bank vole *Prnp* gene was obtained from genomic DNA of bank vole I109 by PCR using the oligonucleotides 5′ AGGAGATATACCATGAAGAAGCGGCCAAAGCCTGG3′ and 5′ GTGATGGTGATGTTTGGAACTTCTCCCTTCGTAGTA3′ and cloned in the pOPIN E expression vector. *E. coli* Rosetta™ (DE3) Competent Cells (EMD Millipore) were transformed with the expression vector using standard molecular biology procedures allowing the expression of the recombinant protein in LB broth (Pronadisa) upon Isopropyl β-d-1-thiogalactopyranoside (IPTG) (Gold biotechnology) induction. Purification of the protein was performed with a histidine affinity column (HisTrap FF crude 5 ml, GE Healthcare Amersham) taking advantage of the natural His present in the octapeptide repeat region of PrP. After elution in buffer (20 mM Tris-HCl, 500 mM NaCl, 500 mM imidazole and 2 M guanidine-HCl, pH 8), the quality and purity of protein batches was assessed by BlueSafe (NZYtech) staining after electrophoresis in SDS-PAGE gels (BioRad). Finally, guanidine-HCl was added, to a final concentration of 6 M, for long-term storage of purified protein preparations at − 80 °C. Throughout the work, different batches of protein were produced from the same transformed bacterial colonies and as described above. No independent batches were mixed for the experiments presented.

### Preparation of PMSA substrates

Preparation of recombinant bank vole PrP-based in vitro propagation substrates was performed as described previously [30]. Briefly, the purified rec-PrP stored with 6 M of guanidine-HCl was diluted 1:5 in phosphate buffered saline (PBS, Hyclone) and dialyzed against PBS at 1:2000 ratio for 1 h at room temperature. The dialyzed sample was centrifuged at 19,000*g* for 15 min at 4 °C and the supernatant used for substrate preparation. Rec-PrP concentration in the supernatant was measured (BCA protein assay kit, Thermo Scientific) and adjusted to the working concentration, which, unless otherwise indicated, was of 20 µM to reach a final concentration of 2 µM when diluted in the substrate. The protein, after dialysis and concentration adjustment was mixed with conversion buffer (CB) [34] 1:9, and dextran sulfate sodium salt from *Leuconostoc *spp. with molecular weights ranging from 6500 to 10,000 (Sigma-Aldrich) was added to a final concentration of 0.5% (w/v). The substrate was aliquoted and stored at − 80 °C until required. From each independent protein batch, several substrate preparations were done, performing independent dialysis for every preparation. Distinct substrate preparations were not mixed for the same experiment.

### PMSA reaction for spontaneous misfolding of recombinant PrP and its in vitro propagation

PMSA was initially performed using rec-PrP derived substrates supplemented with dextran sulfate placed in 2 ml tubes with conical bottom and screw cap (Fisherbrand). The reaction temperature was set at 39 °C using either a Thermomixer (Eppendorf) or a Digital shaking Drybath (ThermoScientific) with internal temperature control, and shaking at 700 rpm continuously for 24 h PMSA rounds unless indicated otherwise. At first, 0.1–0.15 g of 1 mm zirconium silicate beads (BioSpec Products, Inc.) were added to the reaction tubes, which were also used in all prion propagation experiments done by serial dilution of the seed (from 10^–1^ to 10^–9^). Alternatively, other bead types were used: 1.5 mm diameter steel beads (BioSpec Products, Inc.), 1.5 mm diameter Teflon® beads (Marteau & Lemarie), 1 mm borosilicate beads (Sigma-Aldrich), 1 mm acid-washed glass beads (BioSpec Products, Inc.), 1 mm silicon carbide beads (BioSpec Products, Inc.), and 1 mm zirconium beads (BioSpec Products, Inc.). Once glass beads were chosen for spontaneous misfolding, 0.1–0.4 g of acid-washed 1 mm glass beads were regularly added to each PMSA tubes, unless specified otherwise in experiments in which bead amount was modified. Also, glass beads from different sizes were used in some experiments such as glass beads of 0.15–0.21 mm, 0.21–0.3 mm, 0.42–0.6 mm and 0.7–1.18 mm (all from Sigma-Aldrich), 9–13 µm diameter glass spheres (Sigma-Aldrich) and 0.1–0.2 mm diameter glass beads (Sigma-Aldrich). And in a single experiment, acid washed and unwashed glass beads of ≤ 106 µm were compared (Sigma-Aldrich). In all cases, serial 24 h PMSA rounds were performed at 1:10 to 1:1,000 dilutions using the same bead type and amount at each round.

### Loading of prions in zirconium silicate beads for long term storage and use as a seed

Potential recombinant prion strains, characterized by a particular electrophoretic banding pattern after PK digestion, formed in PMSA, were stabilized for long-term storage and posterior propagation or expansion. Stabilization was performed through a slightly modified PMSA procedure. The PMSA product in which each potentially different recombinant prions were first detected was used as seed to inoculate a fresh PMSA substrate containing the same bank vole rec-PrP at 1:10 dilution (seed:substrate). Instead of adding glass beads to the reaction tube, which stimulate spontaneous rec-PrP misfolding, 1 mm diameter zirconium silicate beads (BioSpec Products, Inc.) were used that favour propagation of pre-formed prions. Additionally, these beads show the capacity to bind to prions during the PMSA reaction, getting coated with recombinant prions propagated from the original seed. After 24 h PMSA reaction at 39 °C and 700 rpm, the zirconium silicate beads were recovered from each tube, washed thoroughly with PBS and used as seeds for a second PMSA round in which more non prion-coated zirconium silicate beads were added. 3 to 6 serial 24 h PMSA rounds using fresh-substrate and mixing prion coated and non-coated beads were performed until obtaining the desired amount of recombinant prion-coated beads, which after washing with PBS thoroughly and air drying can be conserved during years at room temperature until their use as seeds. In all cases, conservation of the electrophoretic pattern from the original seed in the prion-coated beads was checked by an additional PMSA round, using 1–5 prion-coated beads as seed. After a 24 h PMSA reaction at 39 °C and 700 rpm, the supernatant was analysed by proteinase K digestion, electrophoresis and total protein staining as detailed below.

### Misfolded recombinant PrP detection

PMSA products were transferred from the reaction tubes to clean Eppendorf tubes and digested by adding proteinase K (PK) (Roche) at 25 µg/ml for 1 h at 42 °C in an oven (Nahita). Immediately after digestion, samples were centrifuged at 19,000*g* at 4 °C for 15 min, the supernatant was discarded and the pellet resuspended and washed with at least 700 µl of PBS (Fisher Bioreagents). After washing, samples were centrifuged for additional 5 min at 19,000*g* and 4 °C, supernatant discarded, and the pellet resuspended in 15 µl of loading buffer 4 ×  (NuPage LDS, Invitrogen), previously diluted to 1 × with PBS. PK-resistant PrP detection was done through electrophoresis and total protein staining. For that, PK-digested and concentrated samples in loading buffer were boiled for 10 min at 100 °C and loaded onto 4–12% acrylamide gels (NuPAGE Midi gel, Invitrogen Life Technologies), subjected to electrophoresis for 1 h and 20 min (10 min at 70 V, 10 min at 110 V and 1 h at 150 V) and stained with BlueSafe (NZYTech) for 1 h at room temperature. The same procedure was done for the determination of PK resistance of one of the PMSA products, but using 25, 100, 200, 500, 1000, 2000, 2500 and 3000 µg/ml of PK in a previously aliquoted sample. Additionally, PK digestions were also performed in one case using 25 µg/ml of PK at room temperature and 42 °C, for 6 and 24 h without shaking.

### PMCA for the assessment of the potential infectivity of recombinant misfolded PrP

#### Preparation of PMCA substrate

Perfused whole brains of TgVole 1 × mouse model (see details on the model in the next section), transgenic mice expressing onefold bank vole I109 PrP under the control of the murine PrP promoter in a murine Prnp^0/0^ background [30], were homogenized at 10% (w/v) in CB with protease inhibitor cocktail (Roche) in a glass potter pestle (Fisher scientific), aliquoted and stored at − 80 °C until required.

#### Preparation of recombinant seeds

PMSA products containing rec-PrP^res^ were purified prior to their use as seeds for brain homogenate-based PMCA. For that, 6 ml of each PMSA product were ultracentrifuged through a continuous cesium sulfate gradient. This gradient was prepared mixing two Cs_2_SO_4_ (Sigma-Aldrich) solutions in PBS, one at 1 M and the other at 1.7 M, with a 15 ml gradient mixer (Sigma-Aldrich), then placed in Thinwall Ultra-Clear, 13.2 ml, centrifuge tubes (Beckman Coulter). PMSA products concentrated through sedimentation to 2 ml were afterwards placed on top of the gradient and submitted to ultracentrifugation, using a SW41 Ti Swinging bucket rotor (Beckman Coulter) and an Optima L-90K ultracentrifuge (Beckman Coulter), at 210,000*g* for 15 h at 20 °C. Once centrifuged, fractions in which visible precipitated halos were detected, were transferred to 5 ml Eppendorf tubes and diluted with the maximum volume of MilliQ grade water. After centrifugation at 4000*g* for 30 min, the resuspended pellet was transferred to a 1.5 ml Eppendorf tube and washed with 1 ml of PBS and 15 min centrifugations at 19,000*g*, repeating the PBS resuspension and centrifugation steps at least two times for each halo. Finally, the purified fractions, resuspended in 25–50 µl of PBS, were used as seed in PMCA reactions.

#### Brain-PMCA

Brain-PMCA was performed based on modified versions of the PMCA described previously [13, 60, 61], to estimate the potential in vivo infectivity of the recombinant seeds generated prior to bioassays [33]. Using TgVole 1 × brain homogenates as substrates, seeded at 1:10 dilution with the previously purified recombinant seed, a 24 h PMCA was performed in an S-4000 Misonix sonicator with microplate system (Qsonica) with incubation cycles of 30 min followed by sonication pulses of 20 s at 80% power at 38 °C regulated by a circulating water bath. To avoid cross-contamination, all PMCA tubes were sealed with plastic film (Parafilm) prior to introduction in the bath sonicator to prevent accidental opening. Unseeded tubes were also included in the same PMCA round as controls for spontaneous misfolding or cross-contamination.

### Bioassay in TgVole 1 × mouse model

TgVole animals, transgenic mice expressing onefold the PrP^C^ from bank voles (*Myodes glareolus*) bearing isoleucine at position 109 (Genbank accession number AF367624) and generated as detailed in [30], were used for all inoculation experiments shown.

#### Preparation of inocula

For the inoculation of rec-PrP^res^, PMSA products were diluted 1:10 in sterile DPBS (Invitrogene), taking care not to include beads of any kind in the final inoculum. The protease-resistant rec-PrP amount, estimated by electrophoresis and total protein staining, was comparable in all samples. To determine specific infectivity of one of the recombinant inocula, the PMSA product was first diluted 1:10 in DPBS, and then further diluted serially from 10^–1^ to 10^–6^. Brain-derived inocula or inocula for the second passages were prepared by homogenizing brains of diseased animals at 10% (w/v) in DPBS with protease inhibitor cocktail (PI, Roche) and further diluting the brain homogenate 1:10 in DPBS to reach a final 1% brain homogenate. Finally, glass sphere-based inoculum was prepared using a prion-coated glass sphere suspension (obtained as detailed in section *Recombinant prion propagation in PMSA using prion-coated glass spheres as seed* of Materials and methods), serially diluted in DPBS from 10^–1^ to 10^–6^ and then further diluted 1:10 in PBS as done with the inocula in solution.

#### Animal inoculation

Groups of 5 to 10 TgVole 1 × animals were inoculated intracerebrally with 20 µl of each inocula into the left cerebral hemisphere using a sterile disposable 27-gauge hypodermic needle while under gaseous anesthesia (Isofluorane, IsoVet®, Braun). The mice were fed ad libitum and were examined twice a week until neurological clinical signs manifested, after which they were examined daily. The presence of prion disease-associated clinical signs was monitored including kyphosis, gait abnormalities, altered coat state, depressed mental state, flattened back, eye discharge, hyperactivity, loss of body condition and incontinence. Clinically affected animals with two or more severe signs, unresponsive mental state or invalidating motor disturbances were culled before neurological impairment compromised their welfare, by exposure to a rising concentration of carbon dioxide. Survival time was calculated as the interval between inoculation and culling or death in days and expressed as days post-inoculation (dpi). *Post-mortem*, the brain was removed and divided sagittally to be stored at -80 °C and fixed in formalin for posterior biochemical and anatomopathological analysis.

TgVole 1 × mice were obtained from the breeding colonies at CIC bioGUNE (Spain) and were inoculated at the University of Santiago de Compostela and Neiker—Basque Institute for Agricultural Research and Development. All experiments involving animals in Spain adhered to the guidelines included in the Spanish law “Real Decreto 53/2013 de 1 de febrero” on protection of animals used for experimentation and other scientific purposes, which is based on the European Directive 2010/63/UE on Laboratory Animal Protection. The project was approved by the Ethical Committees on Animal Welfare (project codes assigned by the Ethical Committee P-CBG-CBBA-0519, NEIKER-OEBA-2021-003, and 15005/16/006) and performed under their supervision.

### Detection of PrP^Sc^ in brain and PMCA samples by Western blotting

Presence of protease-resistant, disease-associated misfolded PrP (PrP^Sc^) in brain homogenates or PMCA products was assessed through PK digestion, electrophoresis and immunoblotting. Western blotting was performed as described previously [41]. Briefly, 10% brain homogenates or PMCA products were diluted 1:1 (v/v) in digestion buffer [2% (w/v) Tween-20 (Sigma-Aldrich), 2% (v/v) NP-40 (Sigma-Aldrich) and 5% (w/v) Sarkosyl (Sigma-Aldrich) in PBS]. Proteinase K (Roche) was added to reach a final concentration of 85 µg/ml to each sample and these were incubated at 42 °C for 1 h with moderate shaking, 450 rpm. Digestion was stopped by adding loading buffer (NuPage 4 × Loading Buffer, Invitrogen) 1:3 (v/v) boiling samples for 10 min at 100 °C prior to electrophoresis. Samples were then loaded on 4–12% acrylamide gels (NuPAGE Midi gel Invitrogen Life Technologies), subjected to electrophoresis for approximately 1 h and 20 min and transferred to a PVDF membrane (Trans-Blot Turbo Transfer Pack, Bio-Rad) using the Trans-Blot® TurboTM transfer system (Bio-Rad). After blocking non-specific antibody binding of the membranes by incubation in 5% non-fat milk powder for 1 h at room temperature, monoclonal antibodies D18 (1:5000) [75], 9A2 (1:4000) (Central Veterinary Institute, Wageningen UR) or Sha31 (1:4000) (Bertin Pharma) were added and incubated for 1 h at room temperature, prior to washing. After incubation with peroxidase-conjugated secondary goat anti-human IgG [H + L, Thermo Scientific or anti-mouse antibody (m-IgGκ BP-HRP, Santa Cruz Biotechnology)], membranes were washed again and developed with an enhanced chemiluminescent horseradish peroxidase substrate (West Pico Plus, Thermo Scientific), using a FluorChem Q (Alpha Innotech) or iBright 750 (ThermoScientific) for image acquisition and the software AlphaView (Alpha Innotech) for image processing.

### Anatomopathological analysis and immunohistochemistry

Transversal sections of the half-brains fixed with 10% phosphate buffered formalin were performed at the levels of the medulla oblongata, piriform cortex and optic chiasm. Samples were embedded in paraffin-wax after dehydration through increasing alcohol concentrations and xylene. Four-micrometer sections were mounted on glass microscope slides and stained with hematoxylin and eosin for morphological evaluation. Additional sections were mounted in 3-trietoxysilil-propilamine-coated glass microscope slides for immunohistochemistry. Immunohistochemistry (IHC) for detection of PrP^res^ was performed as described previously [62]. Briefly, deparaffinized sections were subjected to epitope unmasking treatments: immersed in formic acid and boiled at low pH (6.15) in a pressure cooker and pre-treated with proteinase K (4 µg/mL, Roche). Endogenous peroxidases were blocked by immersion in a 3% H_2_O_2_ in methanol solution. Sections were then incubated overnight with anti-PrP monoclonal antibody 6C2 (1:1000, CVI-Wageningen UR) or exceptionally with 2G11 (1:1000, Bertin Pharma), and subsequently visualized using the Goat anti-mouse EnVision system (DAKO) and 3,3′-diaminobenzidine (Sigma Aldrich) as the chromogen substrate. As a background control, incubation with the primary antibody was omitted. Brain histological lesions (i.e. spongiform change) and PrP^res^ immunolabeling were evaluated under a light microscope by a single pathologist. A semi-quantitative approach was used as previously described [69]. Spongiform lesion and PrP^res^ immunolabeling were separately scored. Fourteen different brain regions were chosen: piriform cortex (Pfc), hippocampus (H), occipital cortex (Oc), temporal cortex (Tc), parietal cortex (Pc), frontal cortex (Fc), striatum (S), thalamus (T), hypothalamus (HT), mesencephalon (M), medulla oblongata (Mobl), cerebellar nuclei (Cm), cerebellar vermis (Cv) and cerebellar cortex (Cc). Scores ranging from (0) absence of spongiosis or immunolabeling: (1) mild, (2) moderate, (3) intense and (4) maximum intensity of lesion or immunolabeling were assigned to each brain area studied. Each area was investigated globally as region for the scoring. Brain profiles were plotted as a function of the anatomical areas which were ordered along the horizontal axis to represent the caudo-rostral axis of the encephalon. Graphs were plotted using Microsoft Office Excel software.

### Cryo-electron microscopy

PMSA product containing Ust02 rec-PrP^res^, approximately 20 ml, was digested with proteinase K at 25 µg/ml and 42 °C for 1 h and purified by ultracentrifugation through a continuous cesium sulfate gradient prior to imaging. After digestion, the sample was concentrated 10 × by sedimentation, reducing the total volume to 2 ml that were washed with Tris-NaCl buffer [10 mM Tris (BioRad) and 100 mM NaCl (Sigma-Aldrich), pH 7.4], centrifuged at 19,000*g* for 15 min twice. Finally, the pellet was resuspended in 2 ml of Tris-NaCl buffer. The concentrated and PK digested PMSA product was then placed in a Thinwall Ultra-Clear, 13.2 ml, centrifuge tube (Beckman Coulter) on top of a continuous Cs_2_SO_4_ gradient ranging from 1 M to 1.7 M and prepared with a gradient mixer (Sigma-Aldrich). After ultracentrifugation, using a SW41 Ti Swinging bucket rotor (Beckman Coulter) at 210,000*g* for 15 h at 20 °C, fractions in which a visible precipitated halo was detected, was transferred to a 2 ml Eppendorf tube and diluted with the maximum volume of Tris-NaCl buffer. After centrifugation at 19,000*g* for 15 min, the pellet was washed with 1 ml of Tris-NaCl buffer and centrifuged again, repeating the washing steps three times. Finally, the purified fraction, was resuspended in 50 µl of Tris-NaCl and Amphipol A8-35 (Jena Bioscience) added for a final concentration of 0.02% (V/V). Once sonicated in a cup horn sonicator (S700, Qsonica) at 80% power for 2 min (4 pulses of 30 s), it was loaded in grids as follows. Quantifoil R 2/2 300-mesh copper grids coated with a thin layer of carbon were glow-discharged immediately prior cryo-EM grids vitrification. Grids, loaded with 4 µl of purified Ust02 recombinant prions were prepared by plunge-freezing in liquid ethane using LEICA EM GP2 Vitrobot and cooled with liquid nitrogen. The following settings were used: chamber temperature at 8 °C under > 95% of humidity, blotting time of 30 s with filter paper, and sample drop volume of 4 µl. Vitrified Ust02 rec-PrP^Sc^ samples were visualized in a 200 kV JEM-2200FS/CR (JEOL) transmission electron microscope, equipped with an UltraScan 4000 SP cooled slow-scan CCD camera (GATAN).

### Scanning electron microscopy (SEM)

To assess whether recombinant PrP and/or misfolded rec-PrP could be bound to the glass beads used in PMSA reactions, glass spheres from 9 to 13 µm of diameter (Sigma-Aldrich) were chosen. Five different samples were prepared for visualization through SEM: Glass spheres washed with distilled water, glass spheres incubated for 1 h at 37 °C and 700 rpm (Thermomixer, Eppendorf) with substrate devoid of rec-PrP, glass spheres incubated in the same conditions with bank vole rec-PrP containing PMSA substrate, glass spheres submitted to PMSA for four 24 h serial rounds, seeded 1:10 in the first one with Ust02 recombinant prions, letting spheres sediment for 15 min after each round and replacing the supernatant for fresh PMSA substrate. After the last PMSA round, glass spheres were harvested and divided in to parts, submitting one half to proteinase K digestion at 5 µg/ml for 1 h at 42 °C and 450 rpm. Both, PK-treated and untreated glass spheres were thoroughly washed with distilled water, and as all the rest of spheres were oven-dried before preparing for visualization. Once all dried, they were sputtered with gold at 25 mA (Emitech K550X, Aname) and analysed by secondary electron emission with a Hitachi S-4000 SEM, operated at 5 kV (SGIker, UPV/EHU).

### Mass spectrometry

2 ml of the PMSA product were digested by the addition of PK to a final concentration of 25 µg/ml and incubation at 42 °C for 1 h. After digestion, samples were centrifuged for 30 min at 19,000*g*, the supernatant was discarded and the pellet was resuspended and washed with 1 ml of PBS. Subsequently, the sample was centrifuged again for 30 min at 19,000*g*, the supernatant was discarded and the pellet was resuspended in 50 µl Gdn/HCl 6M with 3 pulses of a tip sonicator and incubated for 1 h at 37 °C. After incubation, TFA was added to a final concentration of 1%. Samples (4 µl) were injected into a micro liquid chromatography system (Eksigent Technologies nanoLC 400, SCIEX) coupled to a high-speed Triple TOF 6600 mass spectrometer (SCIEX) with a microflow source, and equipped with a silica-based reversed-phase column ChromXP C18 150 × 0.30 mm, 3 mm particle size and 120 Å pore size (Eksigent, SCIEX). A YMC-TRIART C18 trap column was connected prior to the separating column, online (3 mm particle size and 120 Å pore size, YMC Technologies, Teknokroma). After sample loading and washing with 0.1% formic acid in water to remove Gdn/HCl and other non-peptide components of the sample, the flow was switched onto the analytical column and separation proceeded at a flow rate of 5 μl/min with a solvent system consisting of 0.1% formic acid in water as mobile phase A, and 0.1% formic acid in acetonitrile as mobile phase B. Peptides were separated over 40min with a gradient ranging from 2 to 90% of mobile phase B. Data acquisition was performed in a TripleTOF 6600 System (SCIEX, Foster City, CA) using a Data dependent workflow. Source and interface conditions were the following: ionspray voltage floating (ISVF) 5500 V, curtain gas (CUR) 25, collision energy (CE) 10 556and ion source gas 1 (GS1) 25. The instrument was operated with Analyst TF1.7.1 software (SCIEX, USA). Switching criteria were set to ions greater than mass-to-charge ratio (m/z) 350 and smaller than m/z 1400 with a charge state of 2–5, mass tolerance 250 ppm and an abundance threshold of more than 200 counts (cps). Former target ions were excluded for 15 s. The instrument was automatically calibrated every 4 h using tryptic peptides from PepCalMix as an external calibrant. For data analysis, the sample TIC was opened using the PeakView2.2 software that allows protein reconstruction. The LC–MS Peptide Reconstruct feature uses a peak finding algorithm to identify groups of peaks that form isotope series and charge series. Protein deconvolution was carried out between 800 to 20,000 Da.

### Siliconization of glass beads for PMSA

Siliconization involves placing a thin layer of dimethyldichlorosilane onto a glass surface, making it extremely hydrophobic and preventing binding of many biomolecules. This procedure was used to siliconize glass beads prior to their use in PMSA. A 2% (V/V) dimethyldichlorosilane (Sigma-Aldrich) solution was prepared in chloroform (Sigma-Aldrich), and glass beads were submerged in it, moving them around using polypropylene forceps for few minutes. After removing the beads from the solution, the excess of solution was drained and beads were placed in a paper towel for air drying overnight. Finally, the siliconized glass beads were baked for two hours in an oven at 60 °C, washed thoroughly with MilliQ grade water and allowed to air dry prior to their use in PMSA. This was performed at the usual conditions of 39 °C and continuous shaking at 700 rpm for 24 h, with approximately 50 siliconized glass beads of 1 mm diameter per tube, and the control reaction was done with the same amount of non-siliconized glass beads of the same batch.

### PMSA in glass tube versus plastic tube with and without glass beads

To evaluate if a quiescent glass surface could be enough to promote spontaneous rec-PrP misfolding, PMSA was performed in glass tubes (9 mm short thread glass vial, Fisherbrand, Fisher Scientific), complemented or with glass beads. The internal glass surface of the tube was of approximately 125π mm^2^, so the amount of beads added to plastic 2 ml Eppendorf tubes that served as control was of 0.35 g, representing also 125π mm^2^. The PMSA was performed with the glass tube without and with 0.35 g of 1 mm diameter glass beads and with the usual plastic tubes without and with the same amount of glass beads. All of them were submitted to a 24 h PMSA in standard conditions of 39 °C and continuous shaking at 700 rpm.

### Immobilization of glass beads in plastic resin for PMSA

To assess whether glass bead movement plays a role in spontaneous rec-PrP misfolding, 1 mm diameter glass beads were embedded in a plastic resin that allowed most of the surface of the beads to be solvent exposed. Fragments of the resin, adding up to 50 embedded glass beads, were spiked to four PMSA reaction tubes and a positive control was included in duplicate with the same number of free glass beads of the same batch. Additionally, two tubes with no beads were added also as negative control. All tubes were submitted to a single 24 h PMSA round in standard conditions.

### Evaluation of rec-PrP adsorption to glass beads

To determine rec-PrP adsorption to glass beads and characterize the kinetics of the phenomenon, triplicates of two tubes were prepared with recombinant bank vole protein dialyzed and diluted in PBS as for the preparation of PMSA substrates, one of them without glass beads and the other with 0.1 g of 0.1 mm diameter glass beads (Sigma Aldrich), which corresponds to 500π mm^2^ of glass surface. Both tubes were incubated in a rotary shaker for 24 h, retrieving aliquots of the supernatant from each tube at time points 0, 15 min, 30 min, 45 min, 1 h, 2 h, 6 h and 24 h. rec-PrP concentration in the supernatant at each time point was measured by BCA protein assay kit (ThermoScientific), following the instructions from the manufacturer.

### Recombinant prion propagation in PMSA using prion-coated glass spheres as seed

To assess the propagation capacity and potential infectivity of recombinant prions adsorbed to glass beads, 9–13 µm diameter clean glass spheres (Sigma Aldrich) were first coated with Ust02 prions. For that, 0.2 g of spheres were added to a PMSA substrate that was seeded with a PMSA product containing Ust02 prions at 1:10 dilution. After 24 h of PMSA at 39 °C and continuous shaking at 700 rpm, supernatant was removed and fresh PMSA substrate and 0.2 g of clean glass spheres were added to the tube with already prion-coated spheres. Three PMSA rounds were performed in total, getting a total of 0.6 g of Ust02 prion-coated spheres. After the final round, the supernatant was taken and stored and glass spheres were washed 3 times with PBS and two additional times with MilliQ-grade water to finally air dry the spheres to be used as seed.

To assess in vitro propagation capacity of prion-coated spheres in comparison to the same prions in solution, dried prion-coated spheres were resuspended in 400 µl of PBS to be used as stock suspension. From this stock 1:10 serial dilutions were performed from 10^–1^ to 10^–8^ on tubes containing PMSA substrate complemented with 3 zirconium silicate beads of 1 mm diameter (BioSpec Products, Inc.). Similarly, the supernatant obtained from the prion-coated glass sphere production process was serially diluted 1:10 (from 10^–1^ to 10^–8^) in the same substrate complemented with zirconium silicate beads, and both sets of tubes were submitted to a 24 h PMSA round at 39 °C and continuous shaking at 700 rpm. Results were evaluated as previously, through proteinase K digestion, electrophoresis and total protein staining. Additionally, to confirm prion coating of glass spheres, the suspension prepared previously was treated with proteinase K as the inocula in solution and then mixed with loading buffer (NuPage 4 × , Invitrogene) to release adsorbed prions, including an undigested suspension as control. These samples were also submitted to electrophoresis and total protein staining as described above.

### Determination of the dynamics of spontaneous misfolding and prion propagation in PMSA

To assess if spontaneous misfolding of rec-PrP is a frequent or an infrequent event, the time required for obtaining detectable levels of rec-PrP^res^ was measured in an unseeded PMSA reaction using 30 glass beads of 1 mm diameter and in two seeded PMSA reactions: one containing just 1 prion loaded glass bead (obtained from a 24 h PMSA in which Ust02 prion strain was generated) and the other with 30 prion loaded glass beads from the same source as the previous one. All reactions were performed with standard PMSA conditions, 39 °C and 700 rpm of continuous shaking, including 4 replicates of each condition. Aliquots from the supernatant of each tube were collected every 30 min during the first hour and a half, and every 90 min from there. Rec-PrP^res^ presence was evaluated as detailed above by digestion with proteinase K, electrophoresis and total protein staining.

## Results

### A highly efficient technique to propagate bona fide prions reveals spontaneous stochastic protein misfolding events

Recently, we presented a new method for the efficient and indefinite propagation of recombinant infectious prions in vitro called PMSA [30]. This easily scalable method is able to propagate recombinant *bona fide* prions with extraordinary efficiency in a chemically defined environment and is aimed to obtain large amounts of prion samples for structural studies. However, as most techniques for in vitro protein misfolding that favor aggregate formation of highly amyloidogenic proteins, it presents an intrinsic issue associated with the stochastic nature of the prion misfolding event. Likely related to its high conversion efficiency, PMSA gave raise to spontaneous, low frequency, stochastic misfolding of the recombinant bank vole prion protein used as substrate into protease-resistant isoforms (rec-PrP^res^), mainly in tubes with highly diluted seeds or unseeded.

Intrigued by the spontaneous formation of potentially *bona fide* recombinant prions, we decided to assess the frequency of occurrence of this phenomenon and determine if the PMSA could serve for the spontaneous formation of prions. For that, 20 independent and unseeded PMSA reactions were set up, each submitted to three serial rounds of 24 h. Rec-PrP^res^ from bank vole with an electrophoretic migration pattern resembling that of previously propagated recombinant prions was found in just one tube at the third round of PMSA (Additional file 1: Fig. S1). The frequency of potential spontaneous misfolding was 0 in the first and second PMSA rounds and 5% in the third. To avoid any possible bias due to differential recognition of proteolytic fragments by primary antibodies, all samples were concentrated approximately 50 times to improve the detection limit and were visualized by total protein staining.

### Only a certain type of beads efficiently drives spontaneous recombinant PrP misfolding

**Fig. 1:**
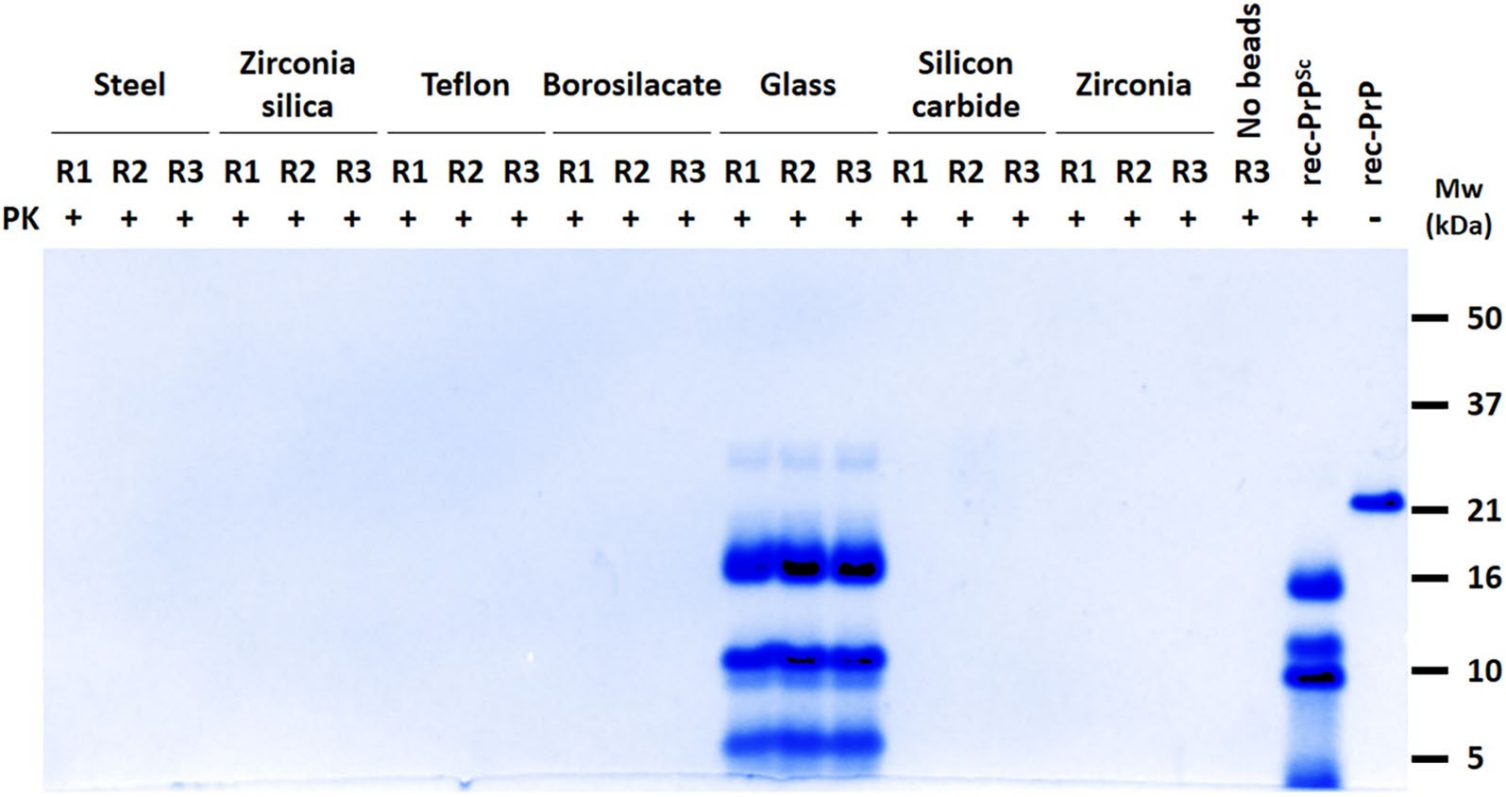
**Assessing the spontaneous formation of prions by PMSA using beads made of different materials.** 1 mm beads made of 7 different materials as well as a negative control without beads were used to evaluate their propensity to spontaneously generate bank vole PrP^res^. A substrate based on bank vole rec-PrP were subjected to three 24 h serial rounds of PMSA (1:10 dilutions). All the samples were concentrated approximately 50 times to improve detection limit and were visualized by total protein staining. Bank vole PrP^res^ was found from the first round of PMSA only when glass beads were used. rec-PrP^Sc^: L-seeded-PMSA recombinant prion strain used as control [38]. rec-PrP: Untreated substrate. Mw: Molecular weight

Given the simplicity of the PMSA and the limited number of elements comprising the propagation system, we decided to further explore some of the individual components and their contribution to the spontaneous formation of rec-PrP^res^. One of the most critical elements for the high efficiency of prion propagation by PMSA is the presence of 1 mm zirconium silicate beads, which seemingly enhance seed-induced recombinant PrP misfolding [30]. Consequently, we tested whether the spontaneous misfolding frequency could be modified using beads made of different materials. With this purpose in mind, 1 mm beads made of 7 different materials were tested (50–70 beads per tube) and their propensity to give rise to PrP^res^ determined, as well as that of a negative control without beads, over three serial 24 h rounds of PMSA. Taking advantage of the large reaction volumes accommodated by PMSA, misfolded and PK-resistant PrP was visualized through total protein staining (Fig. 1).

Interestingly, while zirconium and zirconium silicate beads did not cause spontaneous formation of rec-PrP^res^ in agreement with the low frequency showed in the previous study (5% in third round), glass beads showed induction of spontaneous misfolding, with rec-PrP^res^ detected from the first round of PMSA.

### A highly reproducible and truly spontaneous protein misfolding event

The unexpected behavior of the recombinant bank vole PrP when subjected to PMSA with glass beads, dramatically different than when using other bead materials, required further confirmation. Moreover, the products of spontaneous misfolding obtained in such highly efficient prion propagation systems, arise doubts on their spontaneous nature regarding the potential occurrence of cross-contamination with seeds previously handled within the same laboratory. To address these concerns, three independent rec-PrP batches were produced, substrates were prepared, and newly opened glass beads were added in a prion-free laboratory. Given the simplicity of the system, prion-free shaking equipment could be used, not only completely eliminating the risk of cross-contamination but also showing that this procedure can be easily performed anywhere. Nonetheless, additional negative controls were included to discard any chance of cross-contamination, using one of the substrate replicates with zirconia silicate beads instead of glass beads. Thus, the different batches were submitted to the same PMSA procedure in two independent experiments. The results, shown in Additional file 1: Fig. S2, consistently confirm the effectiveness of the method to induce truly spontaneous rec-PrP misfolding. Notably, rec-PrP^res^ detected in 100% of the tubes from the first round of PMSA, being the negative control with zirconia silicate beads negative in all rounds.

### Recombinant PrP spontaneously misfolded by PMSA exhibits the key biochemical hallmarks of bona fide prions

The self-propagation capacity and protease resistance are distinctive features of *bona fide* prions that can be rapidly assessed in vitro. Therefore, to determine if the spontaneously generated misfolded and partially protease-resistant rec-PrP produced by PMSA are indeed *bona fide* prions, we assessed their self-propagation capacity and the relative resistance to proteinase K (PK). In addition, the spontaneously generated misfolded rec-PrP was used as a seed in PMCA to examine whether these preparations could propagate on a brain homogenate substrate containing brain-derived PrP^C^, indicating their potential in vivo infectivity.

**Fig. 2:**
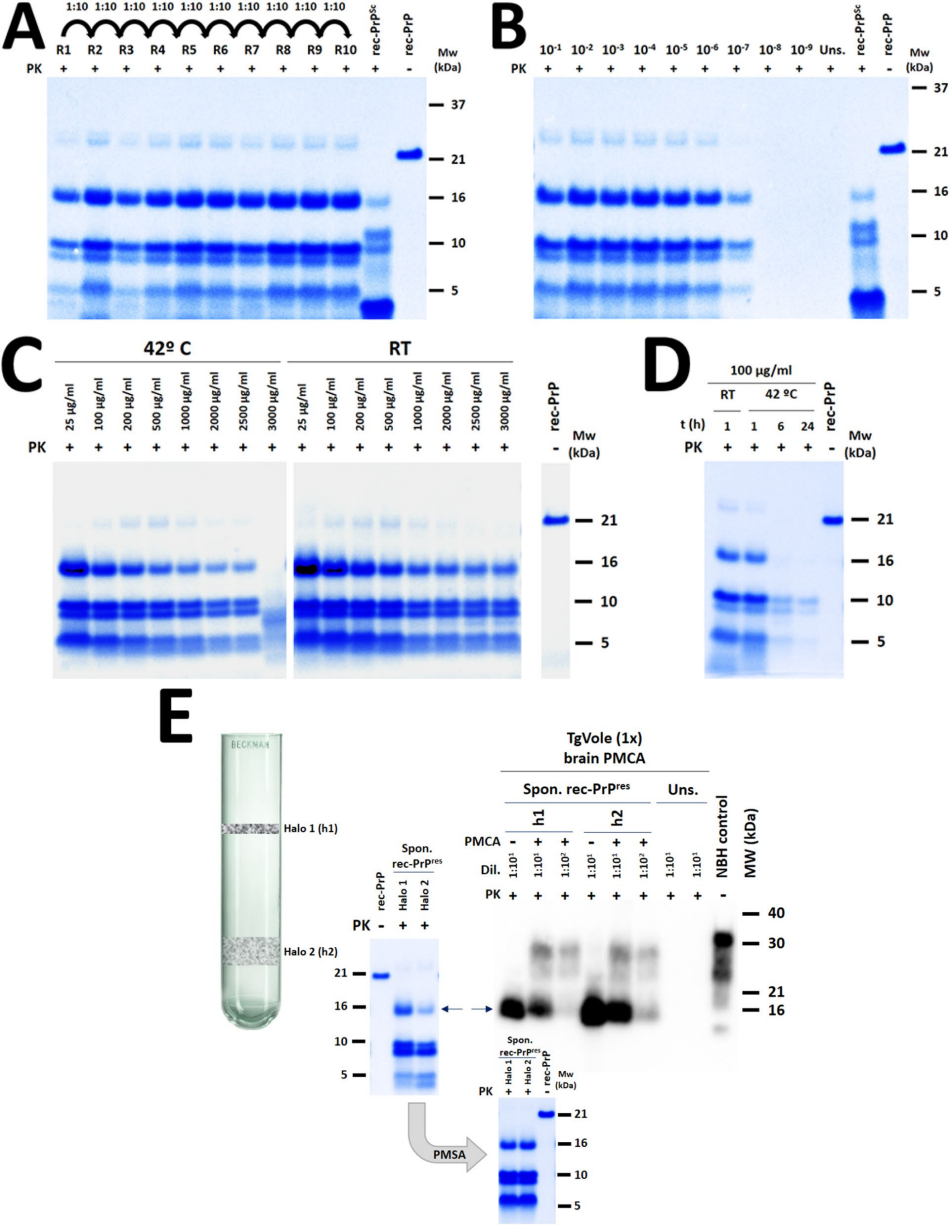
**Biochemical analysis of spontaneously generated misfolded rec-PrP**^**res**^ **to determine the fulfillment of key characteristics expected for** ***bona fide*** **prions**. **A** Evaluation of self-propagation ability by serial PMSA passages. The spontaneously generated PMSA product, complemented with 1 mm zirconia silicate beads, was used to seed a fresh PMSA substrate at a 1:10 dilution. This was followed by a 24 h PMSA round. A total of 10 PMSA rounds, using a 1:10 dilution of the previous round as the seed, were performed. All reactions included 1 mm zirconia silicate beads, which promote propagation without spontaneous misfolding. The samples were concentrated approximately 50 times for improved detection and visualized by total protein staining. A stable and successful propagation was shown by the preservation of the original electrophoretic migration profile after PK digestion. **B** Evaluation of self-propagation ability by serial dilutions in PMSA. The spontaneously generated PMSA product was serially diluted from 10^–1^ to 10^–9^ in fresh substrate, supplemented with 1 mm zirconia silicate beads, and subjected to a single 24 h PMSA round. An unseeded sample served as a negative control. The samples were concentrated approximately 50 times for improved detection and visualized through total protein staining. After PK digestión, PrP^res^ was detectable up to a dilution of 10^–7^, indicating high efficiency in in vitro propagation. **C**, **D** Evaluation of the proteinase K resistance of the spontaneously generated rec-PrP^res^. The resistance to proteinase K (PK) digestion of the spontaneously generated misfolded rec-PrP by PMSA was assessed using the product obtained from the serial PMSA propagation. The samples were digested with increasing concentrations of PK, ranging from 25 to 3000 µg/ml, at 42 °C and at room temperature (RT) for 1 h. The rec-PrP^res^ propagated from the spontaneously generated seed exhibited high resistance, withstanding up to 2500 µg/ml at 42 °C and up to 3000 µg/ml at RT. This fulfilled another characteristic commonly observed in brain-derived prions. Additionally, the same sample underwent PK digestion with 100 µg/ml at 42 °C for 6 and 24 h, revealing resistance up to 6 h, with 10 kDa fragments remaining resistant even after 24 h of digestion. **E** Assessment of the capacity of the recombinant misfolded PrP to induce misfolding of PrP^C^ from brain in vitro. To predict the potential in vivo infectivity of the spontaneously generated recombinant misfolded PrP (referred to as Spon. rec-PrP^res^) in PMSA, the capacity to induce misfolding of PrP^C^ in brain homogenates of TgVole 1 × animals was evaluated using PMCA. The aggregates were purified through ultracentrifugation using a density gradient, resulting in two distinct visible halos of proteic aggregate (referred to Halo 1, h1, and Halo 2, h2). These purified fractions exhibited indistinguishable biochemical properties to the original product, as demonstrated by proteinase K digestion and retention of the same electrophoretic pattern. These purified fractions were used to seed a PMCA substrate based on TgVole 1 × brain homogenate at dilutions of 1:10 (10^–1^ and 10^–2^ dilutions), and single a 24 h PMCA reaction was performed. An additional seeded tube was included for each halo at the same 1:10 dilution but was not subjected to PMCA, as control of the signal of the seed. Two unseeded tubes were also included as control for spontaneous misfolding or cross contamination. After a 24 h PMCA round, PrP^Sc^ detection was carried out by proteinase K digestion (85 µg/ml, for 1 h at 42 °C) and Western blotting using mAb Sha31 at a dilution of 1:4000. Both fractions showed the capacity to induce PrP^C^ misfolding up to dilution 10^–2^, resulting in a classical three-banded PrP^Sc^ pattern, indicative of potential in vivo infectivity. Furthermore, we assessed the propagation capacity of the same fractions on rec-PrP-based PMSA substrate using PMSA. Both halos were used as seeds at a 1:10 dilution on a substrate supplemented with zirconium silicate beads. Following a single 24 h PMSA round at 39 °C and 700 rpm, the presence of rec-PrP^res^ was evaluated through proteinase K digestion, electrophoresis, and total protein staining. Both fractions were able to propagate in PMSA, exhibiting no detectable differences and maintaining the original electrophoretic patterns of the PMSA product. Uns.: Unseeded. rec-PrP^Sc^: L-seeded-PMSA recombinant prion strain used as control [38]. rec-PrP: Untreated substrate. NBH control: Normal brain homogenate from TgVole 1x. Mw: Molecular weight

The self-propagation capacity was evaluated first through serial PMSA passages, seeding the PMSA substrate at 1:10 dilution of the spontaneously generated product, which was then subjected to a 24 h PMSA. For the following passages, the product from the previous PMSA round was again diluted 1:10 in fresh substrate and subjected to another 24 h PMSA. The results demonstrated stable and successful propagation of the spontaneously generated rec-PrP^res^, retaining its original electrophoretic migration profile after PK digestion (Fig. 2A). Similarly, when the same seed was subjected to a single PMSA round of 24 h at serial dilutions ranging from 10^–1^ to 10^–9^ in fresh PMSA substrate, detectable rec-PrP^res^ was observed up to a dilution of 10^–7^ (Fig. 2B), indicating a high efficiency on in vitro propagation.

The proteinase K resistance of the spontaneously generated seed was assessed by digesting the product of serial PMSA propagation with increasing concentrations of PK at two different temperatures. As shown in Fig. 2C, this rec-PrP^res^ displayed high resistance to PK, withstanding up to 3000 µg/ml at RT and up to 2500 µg/ml at 42 °C, thus fulfilling another characteristic shared by most of the brain-derived prions. Additionally, the same sample was digested at 100 µg/ml of PK for 24 h, further confirming the remarkable resistance of PMSA products to proteolytic digestion (Fig. 2D). In fact, when compared to other well-known brain-derived prion strains, it exhibited equal or higher resistance to protease digestion (Additional file 1: Fig. S3).

Finally, PMCA with brain homogenate as a substrate was conducted. This experimental approach not only further confirms the *bona fide* prion behavior of the recombinant preparation but also suggests its potential infectivity in vivo. This is supported by the observed correlation between the ability of the rec-PrP^res^ to induce PrP^C^ misfolding in vitro and their in vivo infectivity [30, 33]. As shown in Fig. 2E, the spontaneously generated and purified rec-PrP^res^ effectively induced misfolding of bank vole PrP^C^ in PMCA, at least up to dilution 10^–2^. Interestingly, purification through density gradient and ultracentrifugation yielded two distinct fractions (referred to as visible protein halos h1 and h2) with indistinguishable electrophoretic patterns after proteinase K digestion. These fractions exhibited similar capabilities in terms of seeding brain-derived PrP^C^ in vitro as demonstrated in Fig. 2E.

### Distinct electrophoretic migration profiles point towards the spontaneous formation of different conformers

**Fig. 3:**
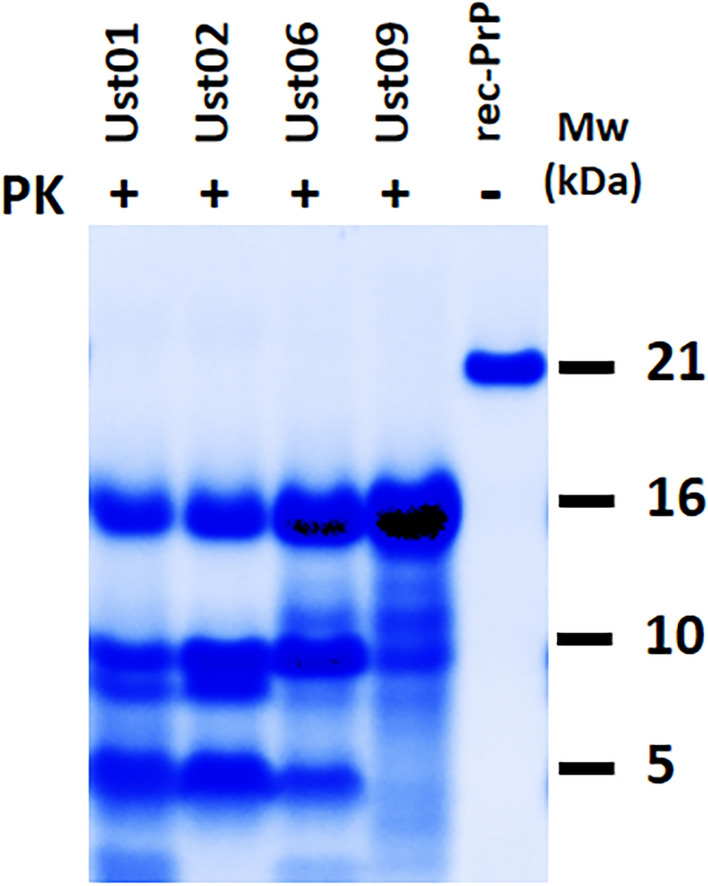
**Representative examples of distinct electrophoretic mobility patterns observed following proteinase K digestion of spontaneously misfolded recombinant PrP in PMSA.** Along multiple PMSA experiments, the spontaneous formation of rec-PrP^res^ with distinguishable electrophoretic mobility patterns was noticed after electrophoresis and total protein staining. These patterns were consistently obtained with varying frequencies. Thorough a comprehensive analysis of the PMSA products generated, we identified four distinct potentially different conformers. After proteinase K (PK) digestion, each conformer exhibited characteristic proteolytic fragments. The gel displays the potential recombinant strains Ust01 and Ust02, which both showed a 16 kDa band, two approximately 10 kDa bands with differing relative intensities (with Ust01 bands being similar and Ust02 bands predominantly displaying the higher band), and fragments of approximately 5 kDa. Additionally, the Ust06 strain exhibited the 16 kDa, 10 kDa, and 5 kDa fragments, along with a mild band over 10 kDa. Lastly, the Ust09 strain displayed an intense 16 kDa fragment and a ladder-like pattern below. rec-PrP: Undigested recombinant PrP. MW: Molecular weight marker

Distinct electrophoretic migration profiles were observed during multiple unseeded PMSA experiments. These variations in migration profiles of rec-PrP^res^, evident after proteinase K digestion, suggest the presence of different proteolytic fragments and, consequently, the existence of diverse misfolded PrP conformers. To further explore this phenomenon, a comprehensive analysis was conducted on the electrophoretic migration patterns obtained from all previous unseeded PMSA experiments. These patterns were carefully examined and classified according to the size and relative intensities of visible fragments following total protein staining. The identified distinct migration profiles are depicted in Fig. 3.

The repeated occurrence of four distinct profiles during spontaneous in vitro misfolding suggests the presence of different conformers, possibly representing distinct strains. These conformers exhibit varying levels of protease resistance and demonstrate the ability to self-propagate when used as seeds at a 1:1000 dilution in PMSA, while maintaining their characteristic electrophoretic migration profiles (Additional file 1: Fig. S4A to 4C).

To enable long-term storage at room temperature, the distinct conformers were loaded into zirconium silicate beads. These beads, after thorough washing and drying, can be used as reaction seeds to further propagation, even after years of storage. In addition, as the previously tested rec-PrP^res^, these potentially different conformers were able to induce misfolding of brain-derived PrP^C^ in PMCA, pointing towards infectivity in vivo (Additional file 1: Fig. S4D). Notably, the pre-purification of the recombinant seeds through density gradient and ultracentrifugation resulted in two fractions with indistinguishable banding pattern following proteinase PK digestion, highlighting their comparable seeding capacities in all cases.

### *Spontaneous misfolding in PMSA consistently generates bona fide prions that are infectious *in vivo* and exhibit distinct strain features*

To confirm the prion nature of the distinct conformers repeatedly obtained spontaneously in PMSA, intracerebral inoculations were performed on TgVole mice expressing 1 × the PrP^C^ from bank voles using the potentially different recombinant PMSA products.

**Fig. 4:**
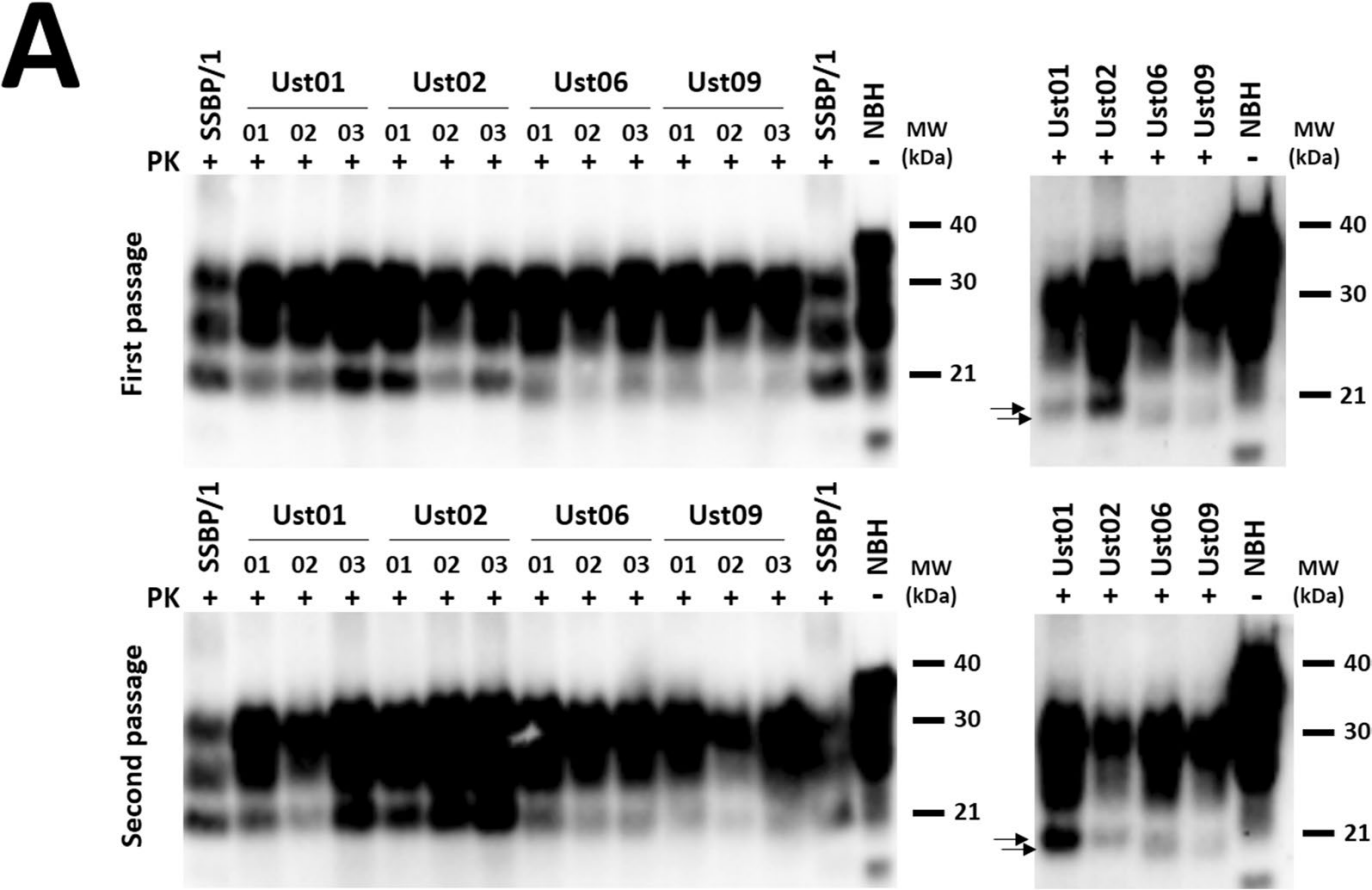

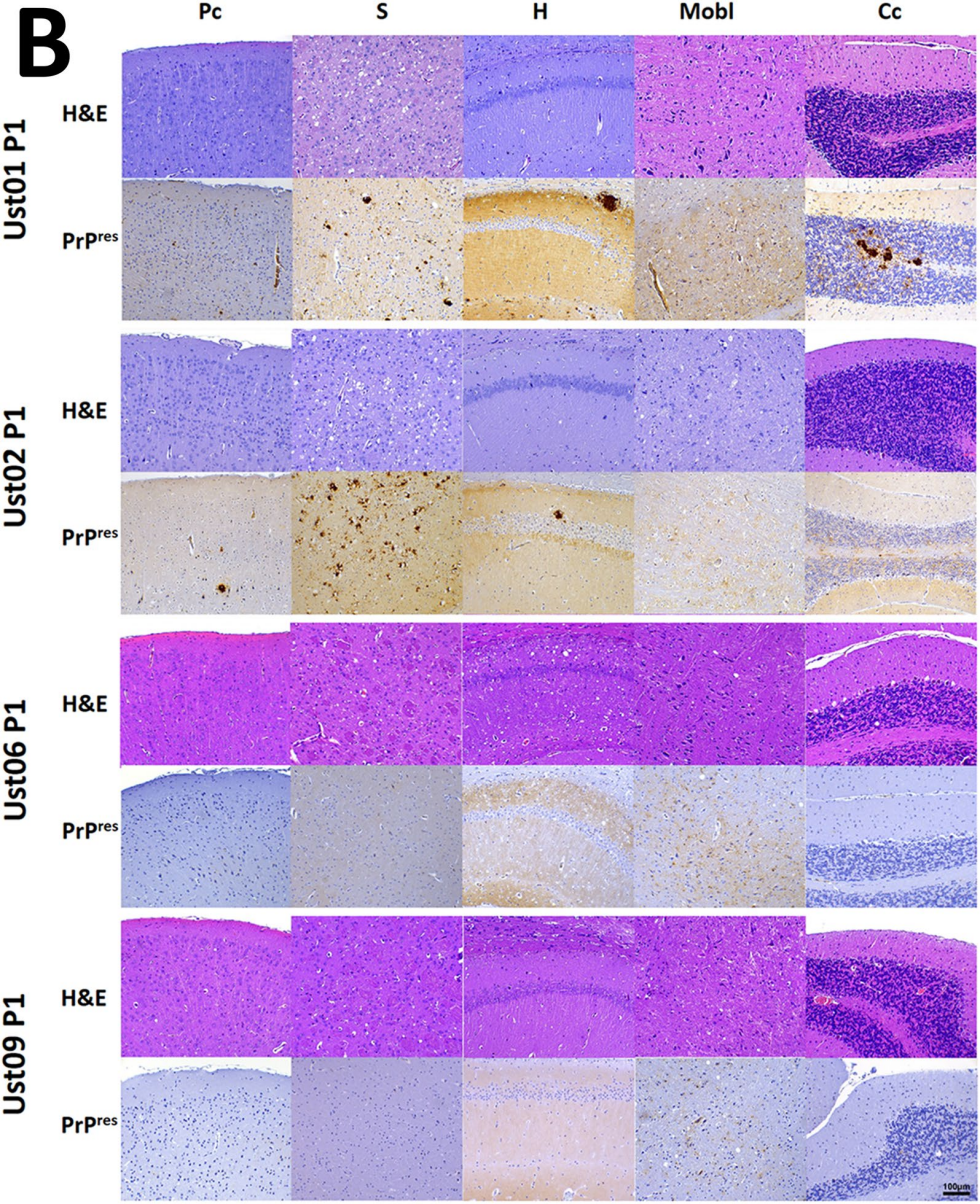

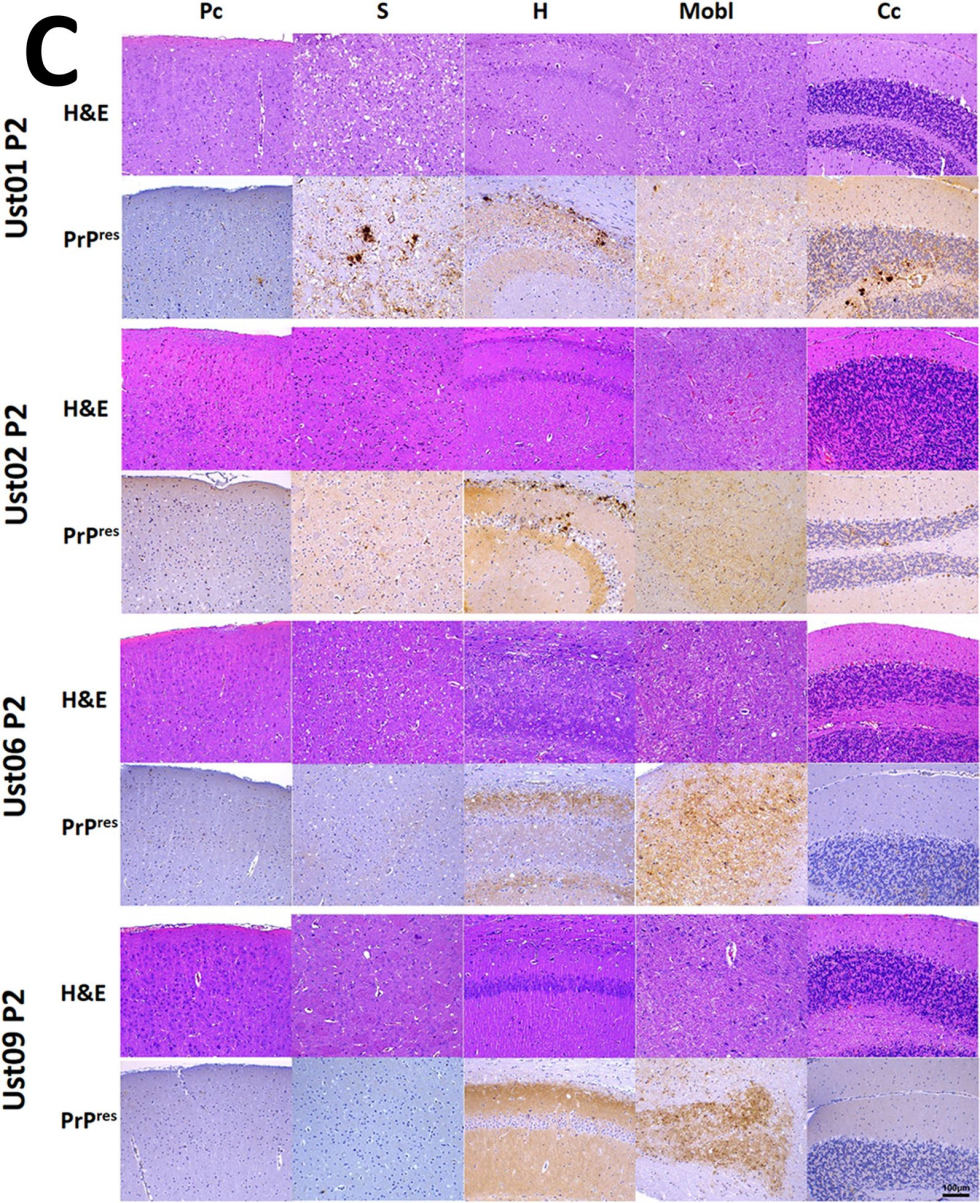
**A** **Biochemical analysis of PrP**^**Sc**^ **in the brains of diseased TgVole 1 × animals inoculated with spontaneously misfolded recombinant prions** Ust01, Ust02, Ust06, and Ust09. 10% homogenates of brains from 3 representative TgVole animals at terminal stage of disease after the intracerebral inoculation with recombinant preparations are shown after proteinase K digestion (85 µg/ml at 42 °C and 450 rpm for 1h) and Western blotting (mAb 9A2, 1:4000). Following inoculation with recombinant preparations, all TgVole 1 × animals exhibited a classical three-banded pattern of PK-resistant PrP^Sc^. Some differences in the molecular weight of the unglycosylated band, apparently lower in Ust06 and Ust09 inoculated animals in both the first and second passage. To facilitate distinction of the size differences on the band corresponding to unglycosilated PrP^res^, some of the samples were selected for a longer electrophoresis run (small membranes in the right) that allowed further separation and confirmed the strain differences (indicated by black arrows). SSBP/1 sheep scrapie isolate was included as a reference for a classical brain-derived prion strain, and undigested normal brain homogenate (NBH) from TgVole 1 × served as a size reference for full length PrP^C^. MW: Molecular weight marker. **B** **Histopathological analysis of spongiosis and PrP**^**res**^ **accumulation in the brains of diseased TgVole 1 × animals** from the first passage inoculated with spontaneously misfolded recombinant prions Ust01, Ust02, Ust06, and Ust09. Formalin-fixed paraffin embedded half-brain sections from TgVole animals at the terminal stage of disease were examined. Sections from different brain regions, including parietal cortex (Pc), striatum (S), hippocampus (H), medulla oblongata (Mobl), and cerebellar cortex (Cc) were stained with Hematoxylin–eosin staining (H&E) to assess vacuolation, and with mAb 2G11 (1:1000) to detect misfolded PrP (PrP^res^). Vacuolation and PrP^res^ accumulation, both indicative of prion disease, were observed in various brain areas with distinct patterns for each inoculum, confirming the authentic prion nature of the recombinant preparations. Supplementary figures provide detailed lesion profiles for each potentially distinct conformers, classified according to their electrophoretic mobility patterns after PK digestion. **C** Histopahtological analysis of spongiosis and PrP^res^ accumulation in the brains of diseased TgVole 1 × animals from the second passage. TgVole 1 × animals were inoculated with 1% brain homogenates from diseased TgVole animals selected based on their incubation time and PrP^Sc^ signal intensity. Similar to the first passage, histopathological analysis revealed vacuolation and PrP^res^ accumulation in all animals, demonstrating the transmissibility of the prion disease induced by the recombinant prions. Detailed lesion profiles for each of the recombinant preparations can be found in Additional file 1: Fig. S6

The fact that 100% of the inoculated animals succumbed to neurological disease (Table 1) clearly confirms the *bona fide* prion nature of the recombinant misfolded PrP^res^. This was further confirmed through the detection of PrP^Sc^ in the brains of all inoculated animals by Western blotting (Fig. 4A), and their anatomopathological analysis (Fig. 4B), which revealed the presence of vacuolation and PrP^res^ deposits. Furthermore, a second passage from TgVole 1 × animals at the terminal stage, after inoculation with each distinct recombinant preparation, demonstrated the transmissibility of the prion disease induced by all of them (Table 1).

**Table 1:** Intracerebral inoculation of spontaneously generated PMSA products with distinct electrophoretic migration patterns in TgVole 1 × and TgVole 4 × animals

Strain	Passage	Substrate	TgVole (1 × ) mice				
			Attack rate	Days post-inoculation (± SEM)	PrP^Sc^ classic pattern (WB)	Spongiosis	PrP^res^ (IHC)
Ust01^a^	1st	rec-Vole	100%	156 ± 6	8/8	8/8	8/8
Ust02	1st	rec-Vole	100%	104 ± 3	8/8	8/8	8/8
Ust06	1st	rec-Vole	100%	201 ± 7	8/8	8/8	8/8
Ust09	1st	rec-Vole	100%	169 ± 5	7/7	7/7	7/7
TgVole-Ust01^b^	2nd	Brain-TgVole	100%	94 ± 2	13/13	13/13	13/13
TgVole-Ust02	2nd	Brain-TgVole	100%	80 ± 3	6/6	6/6	6/6
TgVole-Ust06	2nd	Brain-TgVole	100%	100 ± 1	7/7	7/7	7/7
TgVole-Ust09	2nd	Brain-TgVole	100%	110 ± 2	5/5	5/5	5/5
*TgVole (4×) mice*							
Ust02^a^	1st	rec-Vole	100%	82 ± 1	9/9	9/9	9/9

^a^Recombinant inoculum was prepared diluting the PMSA product 1:10 in PBS. 20 µl of the diluted inoculum were inoculated intracerebrally in each TgVole mouse

^b^For the second passage the brain homogenate of a diseased TgVole (selected for its incubation time close to the mean, and the intensity of its PrP^Sc^ signal in WB), was homogenized at 10% (w/V) in PBS (with protease inhibitor cocktail) and afterwards diluted 1:10 in PBS. 20 µl of the diluted inoculum were injected intracerebrally in each TgVole mouse

A comprehensive histopathological analysis of TgVole 1 × animals inoculated with the recombinant preparations, both directly and upon secondary transmission, revealed distinct lesion profiles (Additional file 1: Fig. S6).

Matching their similarity in terms of proteolytic fragments after proteinase K digestion, the preparations named Ust01 and Ust02 exhibited similar lesion patterns in the first passage. Spongiform changes were predominantly observed in the striatum, accompanied by mild affectation of the medulla oblongata. However, PrP^res^ deposits were also observed in regions without spongiosis, including thalamus, hypothalamus, mesencephalon, medulla oblongata, neocortex, pyriform cortex, and particularly intense in the hippocampus and cerebellar cortex. These deposits consisted in extracellular plaques of varying sizes, as well as granular deposits in the neuropil, occasionally displaying a stellate pattern associated with glial cells. Notably, florid plaques were observed in the striatum. Despite their resemblance in the first passage, these two recombinant prions exhibited striking differences upon secondary transmission. While Ust01 displayed an irregular pattern with some animals showing a vacuolation pattern similar to the first passage, others do not show striatal lesions. Florid PrP^res^ plaques and cerebellar white matter plaques were consistently present in all animals, suggesting a potential mixture of strains or instability of the misfolded PrP in the original recombinant preparation, which may have been further selected or evolved during passages. A similar trend was observed with Ust06, which initially caused mild spongiosis in the striatum, brain stem, and cerebellar nuclei along with evident gliosis in the hippocampus. In terms of PrP^res^ deposits, animals showed a granular pattern in the neuropil and intraneuronal deposits. In the second passage, intense spongiosis was detected in the piriform cortex, hippocampus, the ventral aspect of the occipital cortex, and the neocortex, which was absent in the first passage. Mild brain stem spongiosis and affectation of cerebellar hemispheres and vermis were preserved, unlike the observations in the cerebellar nuclei. PrP^res^ deposits appeared as punctate granular deposits in the same areas as the first passages. In contrast, Ust09 showed a highly conserved lesion profile between the first and second passage. Mild spongiosis affecting the striatum, brain stem, and cerebellar nuclei was observed in both groups, along with granular PrP^res^ deposits in the neuropil and intraneuronal deposits, similar to what was seen in animals inoculated with Ust06 on first passage but without involvement of the hippocampus. In summary, all four recombinant preparations displayed distinct biological features, with potential adaptation of some strains upon secondary transmission. This suggests the possibility of strain evolution in vivo or to the presence of mixtures in the original preparation, which may have undergone further selection during in vivo transmission.

### Recombinant prions generated spontaneously in PMSA show high specific infectivity comparable to those of brain-derived prions

The generation of protein aggregates in vitro that exhibit features of *bona fide* prions, particularly their capacity to induce disease in vivo, often arises concerns about whether the entire preparation consists of infectious prions or if they are merely a minor component within a heterogeneous mixture, with the majority being non-prion amyloids. In order to address this issue, a titration experiment was conducted on one of the PMSA products, the strain named Ust02. This experiment aimed to determine the specific infectivity of the preparation intracerebrally inoculating serial dilutions of the PMSA product into a susceptible model, TgVole 1 × .

Calculations on the specific infectivity of the strain Ust02, using data from Additional file 1: Table S1 (also represented in Additional file 1: Fig. S7) in agreement with the Spearman-Karber method [59], reveal an infectious titer of 4.16·10^5^ LD50/µg of recombinant PrP. Compared to that of a brain-derived strain inoculated in the same model (data and calculation provided in Supplementary information section and Additional file 1: Table S2), this spontaneous recombinant prion preparation exhibits an 80-fold lower specific infectivity than CWD-vole strain, suggesting a high content on infectious prions, not far from that accumulated in the brain of infected animals at terminal stage of disease.

### Electron microscopy and mass spectrometry analysis of Ust02 recombinant prion strain confirms its fibrillary structure and gain insights into the structural arrangement of recombinant prions

To elucidate the ultrastructural features and potential differences between spontaneously formed recombinant prions and brain-derived prions, the Ust02 strain was imaged with cryo-electron microscopy after proteinase K digestion and purification through density gradient and ultracentrifugation. This analysis aimed to assess if the recombinant prions generated in PMSA acquire the expected fibrillary architecture and arrange themselves as prion rods. In addition, mass spectrometry analysis was employed to examine proteolytic rec-PrP^res^ fragments from Ust02 providing valuable information about the structural arrangement of these prions through the identification of sites exposed to protease digestion.

**Fig. 5:**
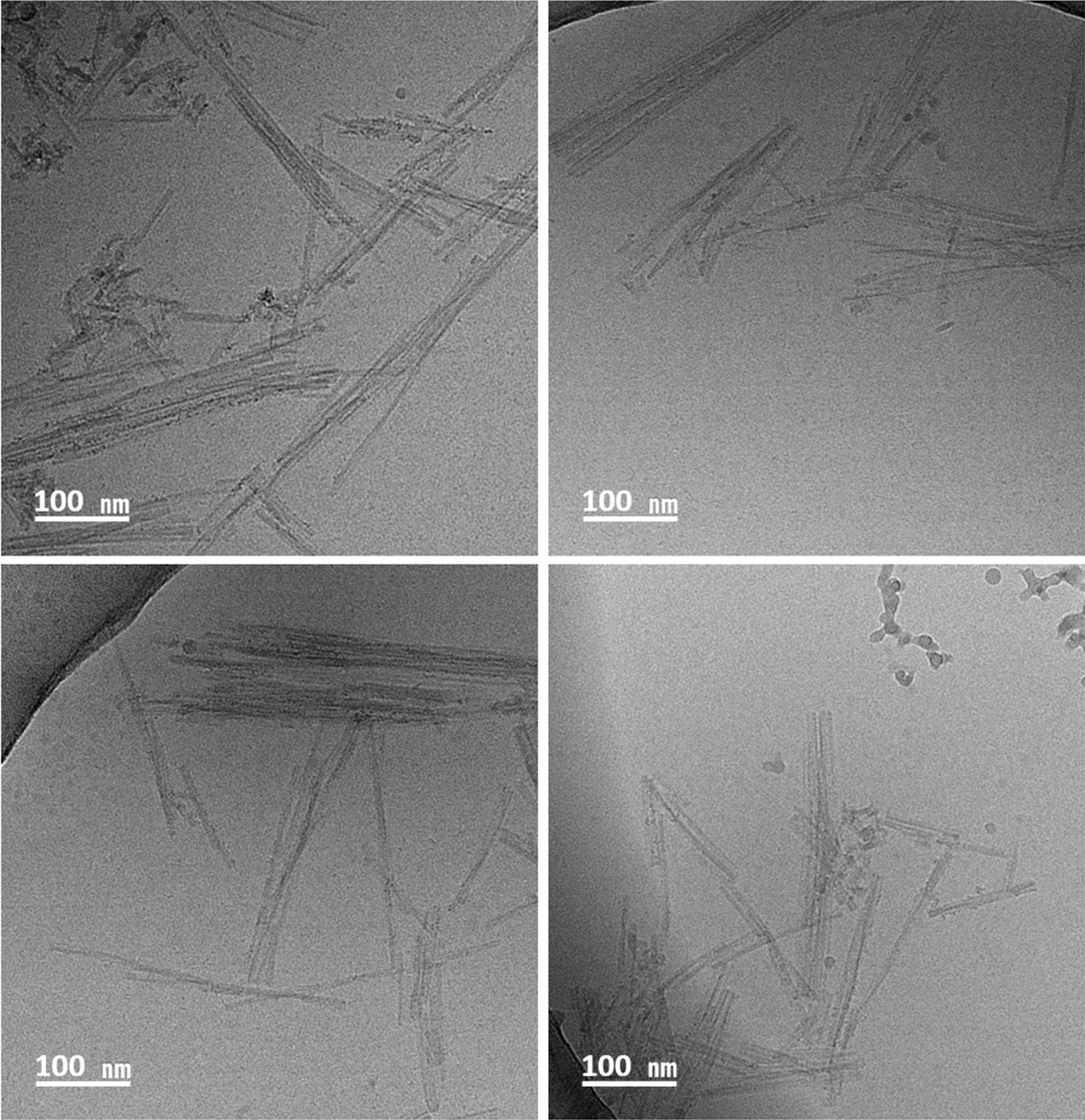
**Cryo-electron microscopy (cryo-EM) micrographs showing the Ust02 recombinant prions. **The images display four representative micrographs capturing the Ust02 prion rods. Utilizing ImageJ software, measurements were conducted to assess the length of the fibers, which ranged approximately from 38 to 450 nm. Similar to brain-derived prions, the observed rods exhibited variable lengths, demonstrating ultrastructural similarities to other prion rods derived from the brain

Upon purification through a density gradient, the PK-digested PMSA preparation was found to be present in a single fraction, presenting as a discernible halo. This fraction was collected, washed, concentrated, and subsequently visualized using cryo-electron microscopy. As shown in Fig. 5, PK-digested and purified Ust02 strain forms elongated, straight fibrils that tend to cluster together in laterally associated fibril bundles. These observations closely resembled the fibrillary structures observed in other prions purified from brains or obtained from field isolates.

Regarding proteolytic fragments identified by mass-spectrometry, the main bands visible in acrylamide electrophoretic gels through total protein staining were identified (Additional file 1: Fig. S8). The upper band, migrating at approximately 16 kDa, corresponds to Q_98_-S_231_ residues of the bank vole PrP. The central band, which is the most intense at around 10 kDa corresponds to the N_153_/M_154_-S_231_ C-terminal fragment. Immediately bellow, another band corresponds to the fragment spanning from residues V_161_/Y_163_ to residue S_231_. Furthermore, several low molecular weight fragments were also identified, aligning with the signal observed in the gels around 5 kDa, mainly corresponding to N-terminal fragments spanning from 96,97,98,99 to 150,153. The most parsimonious interpretation of this patterns is a main cleavage around position 97/98, a secondary “internal cleavage” around position 153, and second, minor internal cleavage around position 161/163.

### The extension of the glass surface is critical for the spontaneous misfolding

Once developed, this simple and robust method provides the opportunity to potentially unravel the key physicochemical factors that promote the spontaneous misfolding of PrP into *bona fide* prions. The changes applied to the previously developed PMSA were analyzed, resulting in highly efficient prion propagation but rare spontaneous misfolding. Previous results using different bead materials clearly demonstrated the critical role of glass beads in promoting spontaneous misfolding. However, it remained unclear whether this effect was determined by bead size, shape, quantity, or their movement. To further investigate the contribution of glass beads to the spontaneous misfolding event, we conducted tests using different amounts of beads with distinct sizes to assess the propensity for spontaneous prion formation.

**Fig. 6:**
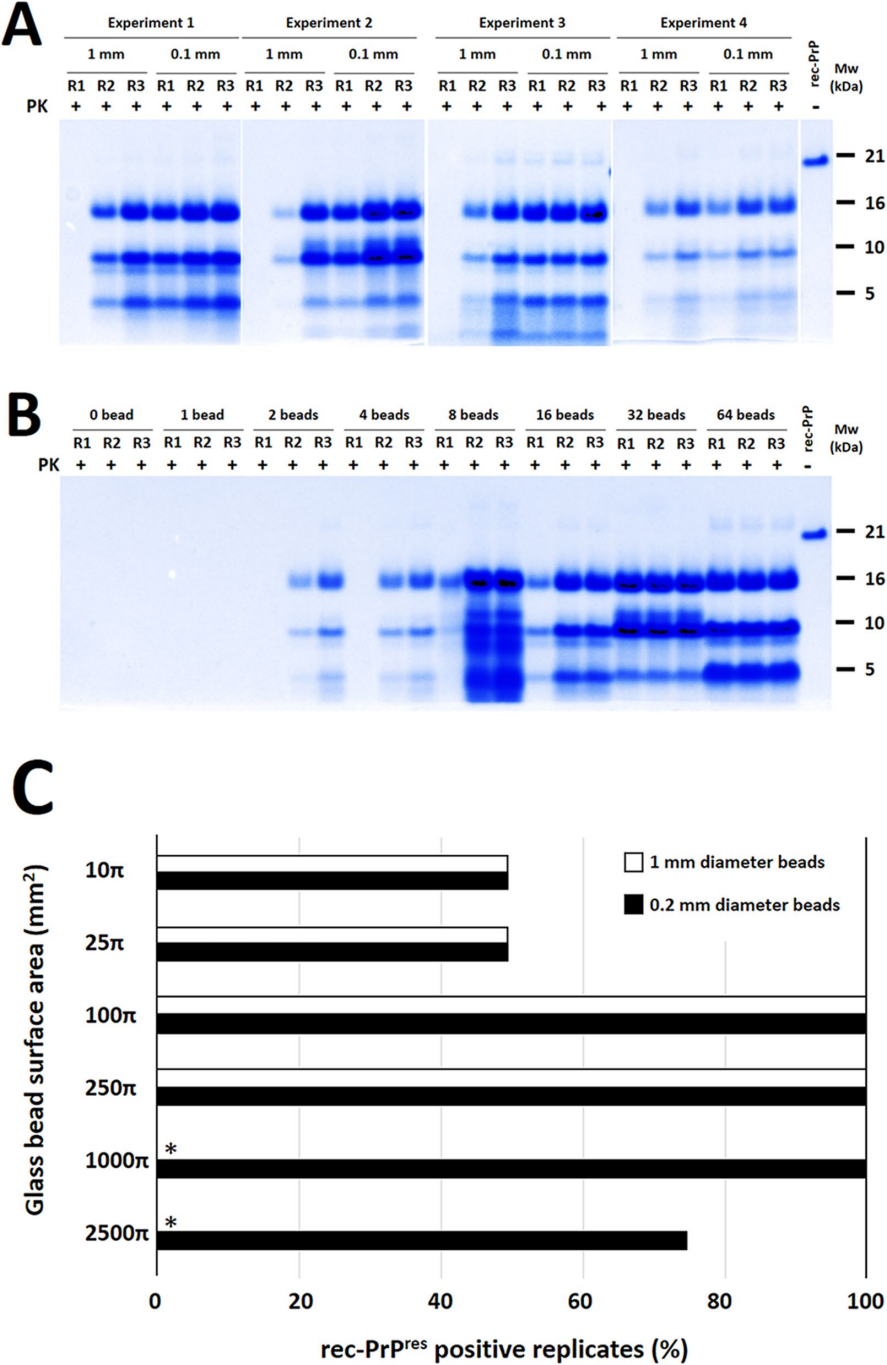
**Evaluation of the effect of glass bead size, amount, and surface on spontaneous misfolding efficiency by PMSA.** **A** The effect of bead size on spontaneous misfolding was first evaluated using glass beads of 0.1 mm and 1 mm diameters, with the same weight (approximately 30 mg of beads per tube) for both types. Four independent experiments were set with each bead type, and three serial 24h PMSA rounds (R1 to R3) were performed. The product from the previous round was used to seed subsequent passages at a 1:10 dilution. Spontaneous rec-PrP^res^ formation was evaluated through proteinase K digestion and electrophoresis, followed by total protein staining. rec-PrP^res^ could be detected from the first round when 0.1 mm beads were used, whereas 1 mm diameter beads required at least two serial rounds to induce detectable levels of misfolding. This suggests that the amount of glass surface area could be a relevant parameter for efficient misfolding occurrence. **B** The effect of bead amount on spontaneous misfolding was examined by using exact numbers of 1 mm diameter glass beads in each reaction tube. PMSA substrate was supplemented with 0, 1, 2, 4, 8, 16, 32 and 64 beads, and each tube was subjected to three serial 24 h PMSA rounds (R1 to R3), with a 1:10 dilution between rounds. Results obtained by proteinase K digestion, electrophoresis, and total protein staining indicate that a minimum of 8 beads is required to give rise to rec-PrP^res^ in the first PMSA round, highlighting the necessity of a sufficient number of beads for efficient spontaneous misfolding in PMSA. **C** To definitively demonstrate the critical parameter for highly efficient spontaneous misfolding of rec-PrP, we conducted experiments using glass beads of two different sizes: 0.2 and 1 mm diameter. These beads were used in quantities that achieved equivalent surfaces areas of 10π, 25π, 100π, 250π, 1000π, and 2500π mm^2^. To ensure robustness, four replicate tubes were used for each surface and bead size. All tubes underwent a single 24 h PMSA round. Subsequently, the formation of rec-PrP^res^ in each tube was determined through proteinase K digestion, electrophoresis and total protein staining. The resulting data was plotted, representing the percentage of rec-PrP^res^ positive replicates for each condition. It is worth noting that the inclusion of the required amount of 1 mm diameter beads to achieve surfaces areas of 1000π and 2500π mm^2^ hindered the free movement of beads inside the tubes. Consequently, the negative results obtained from these conditions were considered inconclusive and were omitted from the plot denoted by an asterisk (*). Our analysis revealed an optimal range of glass surface area between 100π mm^2^ and 250π mm^2^, irrespective of the bead size. This range could potentially extend up to 1000π mm^2^. Surfaces below and above this range showed reduced efficiency in rec-PrP misfolding indicating the existence of a minimum glass surface area that negatively affects spontaneous rec-PrP misfolding efficiency. This reduction may be attributed to the depletion of soluble rec-PrP from the substrate

The comparison between 1 and 0.1 mm diameter beads, with approximately 30 mg of each type per tube, showed differences in the emergence of spontaneously misfolded PrP over three serial PMSA rounds. Tubes containing 1 mm beads (with a surface area of 10-20π mm^2^) required at least two serial PMSA rounds for the formation of rec-PrP^res^, while 0.1 mm beads (with a surface area of around 1000–2000π mm^2^) induced spontaneous rec-PrP^res^ formation in 100% of the tubes from the first round (Fig. 6A). This result contrasts with previous experiments where 1 mm glass beads consistently induced misfolding from the first PMSA round. However, this discrepancy led us to pay attention to the number of beads within the reaction tube, as the higher amount used in the initial experimental settings (e.g. Fig. 1 and Additional file 1: Fig. S2, where 0.1–0.15 g of 1 mm glass beads were used) may play a critical role in the available glass surface. To definitively assess if the critical parameter related to glass beads is the amount or the available surface, PMSA reactions with 0, 1, 2, 4, 8, 16, 32 and 64 beads of 1 mm diameter were performed. As shown in Fig. 6B, maximum induction of spontaneous rec-PrP misfolding was achieved with 8 or more beads, and a *plateau* of maximum frequency was observed at the first PMSA round with 8–16 beads (approximately 8–16π mm^2^). Lower amounts of beads resulted in reduced spontaneous misfolding frequency. This experiment confirmed the requirement of a minimum amount of glass beads or minimum available glass surface for the spontaneous misfolding in our system. In tubes without glass beads or with only one bead, no rec-PrP^res^ was observed even after three serial PMSA rounds. In addition, to further demonstrate that glass size is not critical as long as a minimum amount or, more likely, a minimum surface is used, two different batches of 4 types of glass beads ranging from 0.15–0.21 to 0.7–1.18 mm diameter were added in similar volumes (with surfaces ranging approximately from 50 to 100π mm^2^) to the same PMSA substrate and subjected to three serial 24 h PMSA rounds. As shown in Additional file 1: Fig. S9, regardless of bead size all PMSA products exhibited rec-PrP^res^ from the first round, indicating that as long as the bead surface exceeded 20π mm^2^, specific size or number of beads did not play a critical role. These results suggest that the available glass surface is likely the determining factor for the spontaneous misfolding of rec-PrP.

This was convincingly demonstrated by using glass beads of two different sizes (0.2 and 1 mm diameter) to achieve equivalent surface areas, regardless of the quantity of each bead type. Samples with varying glass surfaces were subjected to PMSA, and it was observed that the maximum efficacy of spontaneous misfolding occurred in tubes with surfaces ranging from 100π to at least 250π mm^2^, irrespective of bead size (Fig. 6C). However, due to technical limitations—inclusion of required 1 mm diameter beads to achieve 1000π or 2500π mm^2^ surface precluded their free movement inside the reaction tube—no conclusive results could be obtained with the largest beads at the higher surface areas. However, the use of 0.2 mm beads allowed evaluation of the effect of largest surfaces, revealing an optimal surface range from 100π to 1000π mm^2^, with a reduction in efficiency detected above 1000π mm^2^. These findings indicate that while a minimum surface is required for efficient induction of spontaneous misfolding, there may also be an upper limit, suggesting that excessive available surface is detrimental, likely due to the depletion of all soluble rec-PrP from the substrate.

The significance of available glass surface in promoting spontaneous prion misfolding was further demonstrated by observing different efficiencies with distinct glass bead batches, some of which were acid washed while others were not. Visually observable differences in the appearance and color of the beads raised suspicions regarding potential variations in the bead fabrication processes, which could explain the divergent results obtained with different batches. Information provided by manufacturer indicated that acid was part of the production process. In light of this, we decided to investigate the effect of acid-washed glass beads versus no acid-washed beads in PMSA (Additional file 1: Fig. S10A). The results showed that the rec-PrP^res^ formation occurred only with beads treated with acid, indicating that surface impurities can also impact the efficiency of spontaneous misfolding. Furthermore, when the glass bead surface was coated with dimethyldichlorosilane (a process known as siliconization), the same beads that previously induced misfolding lost their capacity to promote spontaneous misfolding (Additional file 1: Fig. S10B). Hence, in addition to the amount or surface area of glass beads, the quality and purity of their surface are determinant to induce consistent spontaneous PrP misfolding in vitro.

### The highly efficient spontaneous misfolding also requires the movement or interaction of the glass beads

**Fig. 7:**
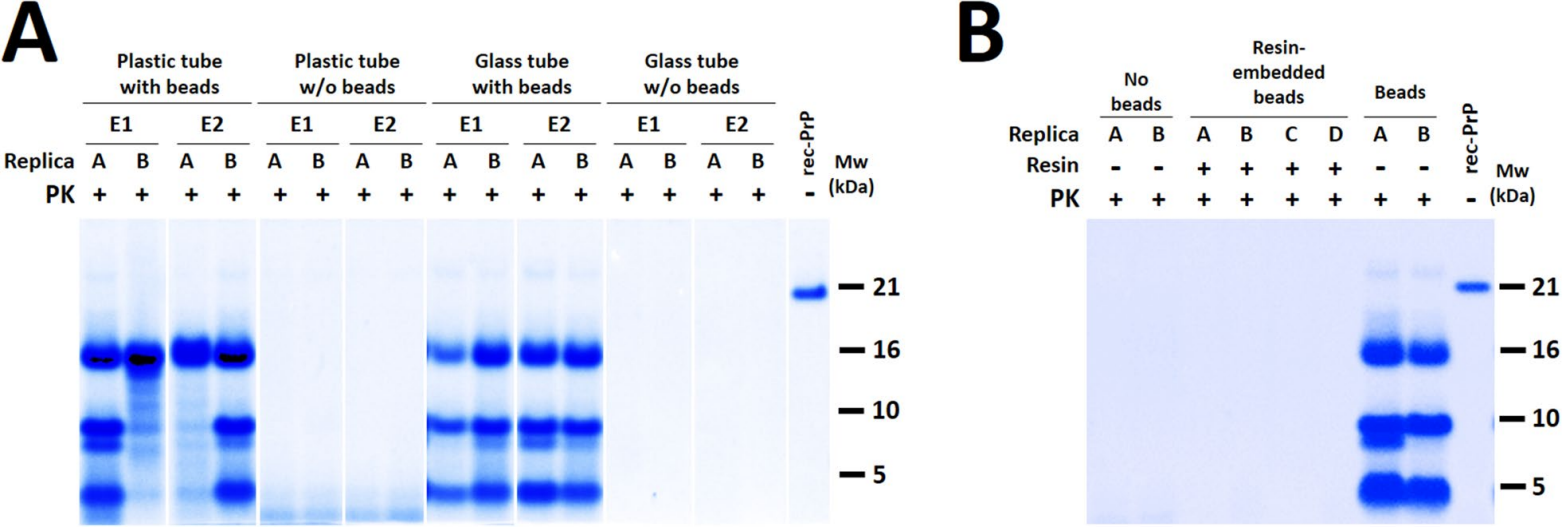
**Evaluating the impact of bead movement on spontaneous misfolding efficiency of recombinant PrP by PMSA.** **A** In order to assess whether bead movement is required along with a minimum available glass surface, glass tubes without beads were used instead of regular plastic tubes in the PMSA reaction. The internal surface of the glass tube is approximately 125π mm^2^, equivalent to the surface of glass beads previously shown to induce spontaneous misfolding from the first PMSA round. The standard plastic tubes complemented with glass beads to achieve a surface area of 125π mm^2^ were used as a positive control, while the glass tubes without beads served as negative control. Two independent experiments (E1 and E2) were performed for each condition, with two replicate tubes each, all subjected to a single 24 h PMSA round. Detection of rec-PrP^res^ through proteinase K digestion, electrophoresis and total protein staining revealed that the presence of glass beads, even within the glass tube, was necessary for efficient misfolding induction. **B** To further investigate the potential significance of bead movement in spontaneous misfolding, 1mm diameter glass beads were embedded in a plastic resin to immobilize them while exposing most of their surface to the PMSA substrate. Four reaction tubes were prepared with resin-embedded beads, accompanied by two tubes without beads and two tubes with the same amount of non-embedded glass beads. All tubes were subjected to a single 24 h PMSA round. Analysis following proteinase K digestion, electrophoresis, and total protein staining, showed no detectable rec-PrP^res^ in the tubes without beads or in the tubes with beads embedded in resin. This demonstrates that bead movement is also critical for efficient spontaneous misfolding in PMSA

While the critical role of a minimum available glass bead surface on spontaneous PrP misfolding in PMSA has been demonstrated, the contribution of continuous and vigorous shaking remains unknown. Since PMSA with other bead types does not give raise to consistent spontaneous PrP misfolding, it is clear that shaking and bead movement alone are not sufficient. However, whether it is necessary in conjunction with an appropriate glass surface needed to be assessed to better understand the critical factors inducing spontaneous prion misfolding. For that, we conducted an experiment replacing the glass surface provided by beads with the internal surface of a glass tube. The positive control consisted of a plastic tube with 1 mm glass beads (total surface of approximately 125π mm^2^), while additional controls included a plastic tube without beads and a glass tube with the same number of beads. All tubes were submitted to a single 24 h PMSA round. The results shown in Fig. 7A clearly demonstrate that rec-PrP^res^ was formed spontaneously only in the presence of glass beads, even though the internal glass surface of the tube was equivalent to that provided by glass beads in the plastic tube. This indicates that both, the availability of a minimum glass surface and the presence of shaking, bead movement, or clashes are necessary for spontaneous prion formation in PMSA. It suggests that the surface energy of glass and other physical factor related to glass bead movement or clashes may play a critical role [6, 7, 16]. Furthermore, the immobilization of glass beads in a plastic resin also resulted in a lack of spontaneous rec-PrP^res^ formation (Fig. 7B), confirming the importance of bead movement in conjunction with the minimum glass surface.

### The interaction between recombinant PrP and the surface of glass beads is essential for inducing spontaneous misfolding

**Fig. 8:**
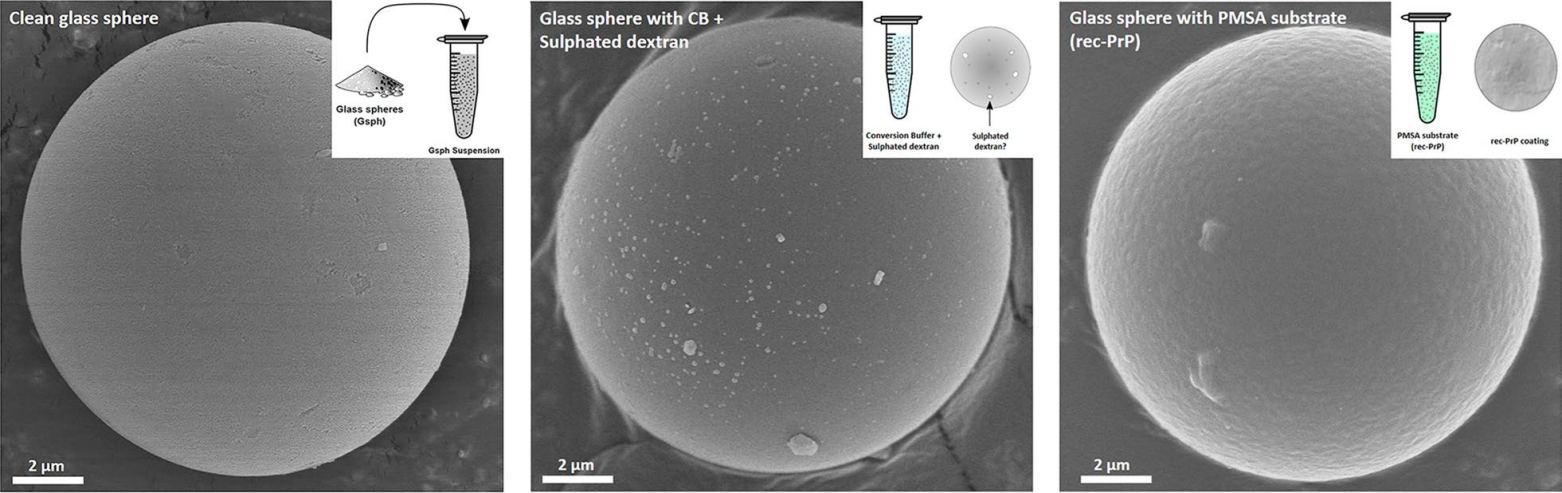
**Scanning electron microscopy (SEM) images of glass spheres incubated with PMSA substrate.** In order to confirm the adsorption of rec-PrP from PMSA substrates onto the surface of glass beads, glass spheres with a diameter of 9 to 13 µm were incubated with PMSA substrate containing bank vole rec-PrP for 2 h. The spheres were then thoroughly washed with PBS and coated with gold for visualization using SEM. Control experiments included glass spheres incubated in PBS (clean glass sphere) and in PMSA substrate devoid of rec-PrP (glass sphere with CB + sulphated dextran), which underwent the same treatment and visualization process. The SEM micrographs clearly show a smooth coating on the spheres incubated with complete PMSA substrate, likely corresponding to rec-PrP, while the spheres incubated in solutions without rec-PrP display a distinct appearance, confirming that rec-PrP is being adsorbed to glass surfaces

If a glass surface and some form of bead movement or clashing are necessary, some interaction between the protein and the glass is assumable. Nonetheless, to gain insight on the mechanisms underlying the spontaneous prion misfolding in PMSA, the binding of rec-PrP to the glass beads needed to be demonstrated. For that, the amount of soluble rec-PrP in the substrate was monitored during a 24 h incubation with 1 mm diameter glass beads (providing a total surface of 500π mm^2^) compared to a control without beads. Electrophoresis and total protein staining of the supernatant revealed a gradual decrease in soluble or free rec-PrP over time in the presence of beads, confirming the binding of rec-PrP to the glass surface, likely through electrostatic interactions (Additional file 1: Fig. S11). This binding was further confirmed by scanning electron microscopy (SEM), which showed a uniform coating of the glass spheres surface that resisted extensive washing, indicating a strong and likely long-lasting binding (Fig. 8).

### *Spontaneously formed rec-PrP*^*res*^* is strongly associated to glass beads, which can act as seeds *in vitro* and are highly infectious *in vivo

**Fig. 9:**
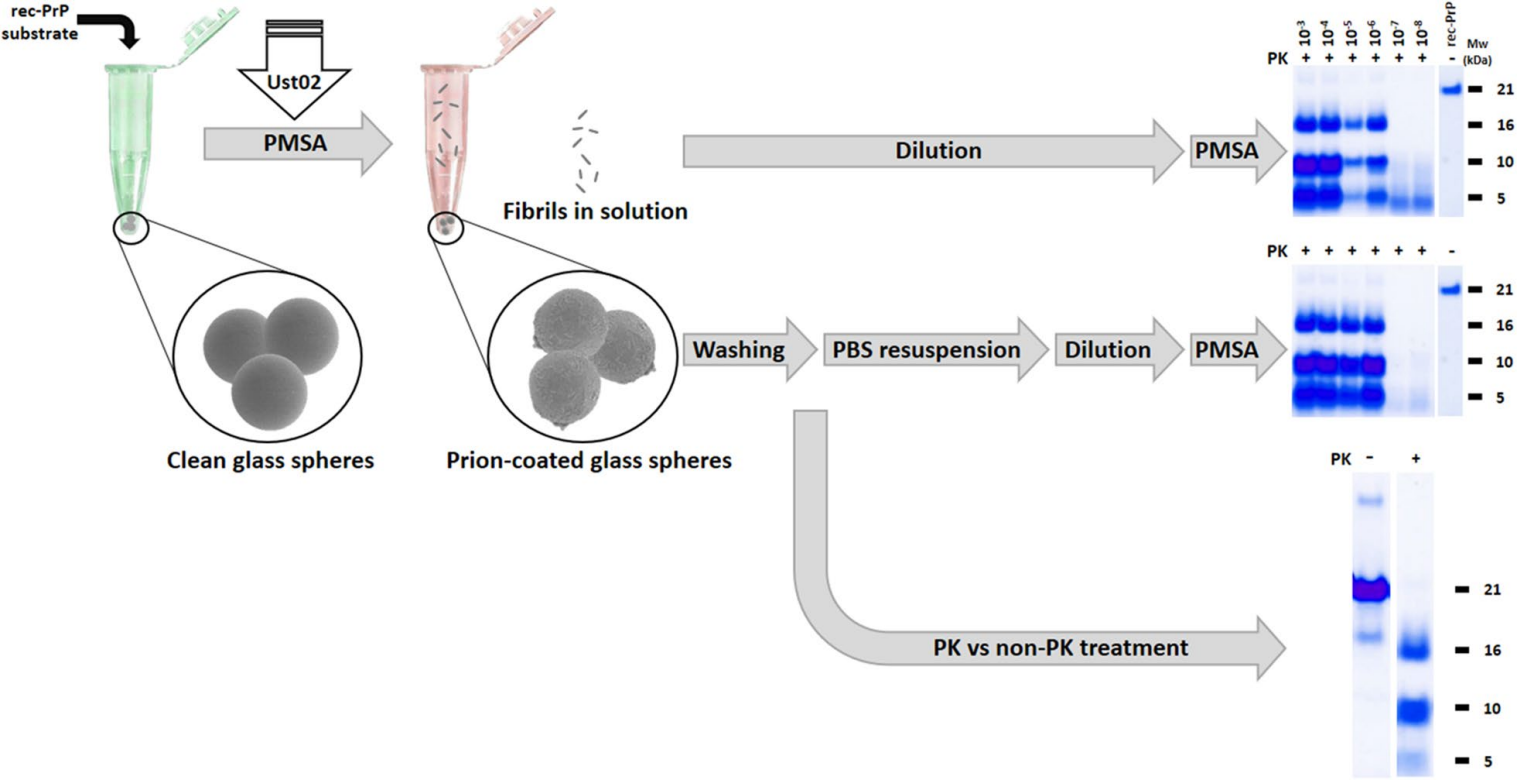
**Assessment of the capacity of prion-coated glass spheres to seed PMSA reactions and confirmation of the adsorption of recombinant prions onto the glass surface.** After a 24 h PMSA reaction, clean glass spheres of 9–13 µm diameter were seeded with Ust02 recombinant prions. The spheres potentially coated with recombinant prions were allowed to settle and separated from the PMSA-product supernatant. While the supernatant was directly used as seed, serially diluted (from 10^–1^ to 10^–8^) in new PMSA substrate complemented with 1 mm zirconia silicate beads, submitting the tubes to a single 24 h PMSA round, the sediment of glass spheres was thoroughly washed with PBS and finally re-suspended in the same volume of Conversion buffer (devoid of rec-PrP) as the original reaction. These solutions with re-suspended glass spheres were serially diluted in the same way as done previously with the PMSA product supernatant. The serial dilutions of the glass sphere suspension were used as seed in the new PMSA substrate supplemented with zirconia silicate beads, and a single 24 h PMSA round was conducted. Results of the PMSA comparing the seeding capacity of PMSA supernatant and the suspension of prion-coated glass spheres, were monitored by proteinase K (PK) digestion, electrophoresis, and total protein staining. In both cases, rec-PrP^res^ was detected in tubes seeded with dilutions 10^–6^ of PMSA product or sphere suspension, showing similar seeding abilities and demonstrating that glass spheres are indeed coated with recombinant prions that conserve their seeding capacity. Additionally, the prion-coated glass sphere suspension used as seed was also digested directly with proteinase K after extensive washing. Once digested, the glass spheres were re-suspended in loading buffer (NuPage 4 × ) and the resulting supernatant, excluding the glass spheres, was loaded into an acrylamide gel and stained for total protein. As shown in the gel below, the prions released from the glass spheres after digestion show the expected electrophoretic pattern for Ust02 prions, confirming their adsorption to the glass surface. MW: Molecular weight marker

**Fig. 10:**
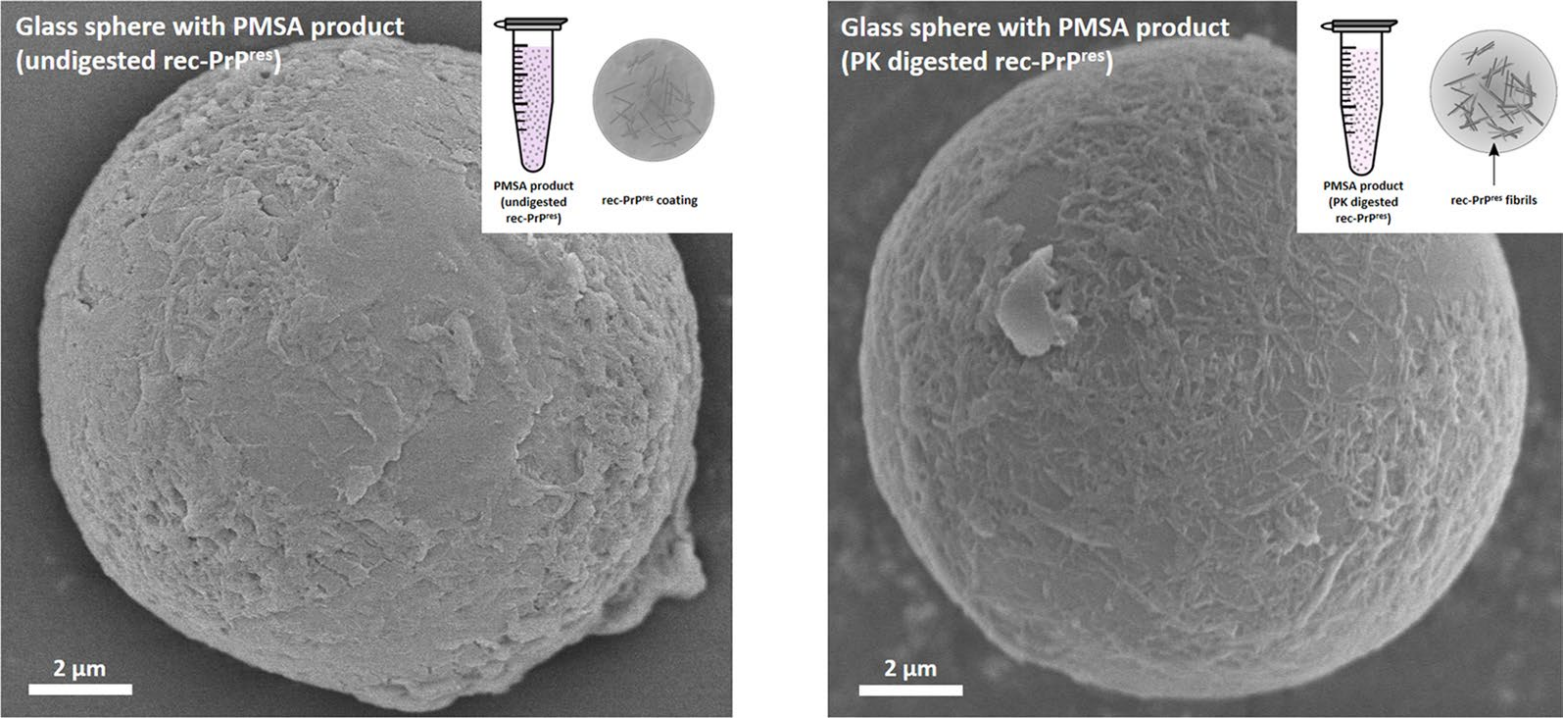
**Scanning electron microscopy (SEM) images of glass spheres recovered after a PMSA round.** To definitively address if rec-PrP^res^ generated during the PMSA process was being adsorbed to the surface of glass beads, we utilized glass spheres with a diameter of 9 to 13 µm in an unseeded 24 h PMSA reaction. Afterwards, the spheres were let to settle, the supernatant was discarded, and the spheres were thoroughly washed with PBS before being completely dried in an oven at 42 °C. Upon resuspension, the sphere suspension was divided in two portions. One half underwent digestion with proteinase K while the other half was simply incubated in the same conditions without digestion. Finally, both PK-digested and undigested spheres were thoroughly washed and air dried before sputtering with gold for visualization using SEM. The micrographs reveal that both digested and undigested spheres exhibit a substantial coating, which diminished upon digestion, and shows fibrillary structures attached to the surface of the spheres. This observation confirms the strong adsorption of rec-PrP^res^ to glass surfaces

In the light of the binding of rec-PrP to the glass surface, we wondered if rec-PrP^res^ could also be localized in the surface of the beads. For that, we first recovered the glass beads from a PMSA tube in which spontaneous rec-PrP^res^ formation had occurred in a 24 h PMSA reaction. The beads obtained from this tube were extensively washed to eliminate rec-PrP^res^ from the supernatant that could be weakly bound to the bead surface and afterwards used as seed in a new PMSA reaction with fresh substrate. Negative controls consisted of the same number of beads that were not subjected to PMSA, ensuring that any detected rec-PrP^res^ could be attributed to seeding rather than newly generated rec-PrP^res^. As shown in Fig. 9, beads presumably coated with rec-PrP^res^ exhibited seeding activity in PMSA, confirming their rec-PrP^res^ coating. Moreover, SEM images revealed a fibrillary material coating the glass beads after an unseeded PMSA reaction. This material resisted proteinase K treatment and remained firmly attached to the beads even after extensive washing (Fig. 10), suggesting a strong association between spontaneous prion formation and the glass surface. The presence of rec-PrP^res^ coating of beads was further proven in vivo by inoculation of glass spheres that had previously undergone PMSA. Immunohistochemical analysis of the mouse encephalon 72 h after intracerebral inoculation showed clearly that there is PrP^res^ surrounding the spheres (Additional file 1: Fig. S12). In addition, to demonstrate the capacity of beads to harbor a high load of recombinant prions, glass spheres (9–13 µm diameter) previously used in PMSA were serially diluted and intracerebrally inoculated in TgVole mice. This experiment allowed for the calculation of specific infectivity in glass spheres (data provided in Supplementary material section and Additional file 1: Table S3) compared to direct inoculation of the PMSA supernatant. The specific infectivity is glass spheres was determined to be 3.23·10^3^ LD50/µg of PrP, lower than that of the liquid inoculum, suggesting that prion attachment to glass spheres could reduce their spreading capacity or, alternatively, that adsorption is limited and a majority of infectious PrP^res^ is released into the supernatant during PMSA.

### Exploring the dynamics of spontaneous misfolding and prion propagation in PMSA

The efficiency and consistent formation of spontaneous recombinant prions in PMSA within 24 h or less, along with the high yield of seeded protein misfolding using zirconia-silica beads [30], poses relevant questions regarding the frequency of spontaneous misfolding events. It raises the important question of whether prion propagation is the primary event following the unusual formation of a single or a few misfolding nuclei, or if spontaneous misfolding occurs frequently and propagation is merely a secondary event resulting from the formation of multiple spontaneously misfolded nuclei.

In the context of PMSA, two distinct time intervals might be considered: **t**_**s**_**,** which represents the time required for the spontaneous misfolding of a single propagation nucleus or a *bona fide* misfolded rec-PrP particle; and **t**_**p**_**,** which denotes the time needed for the propagation of rec-PrP^res^. Given that the presence of glass beads facilitates spontaneous misfolding, the minimum duration of PMSA necessary for detectable rec-PrP^res^ formation through electrophoresis and total protein staining, referred to as **t**_**PMSA**_, can be defined as **t**_**PMSA**_** = t**_**s**_** + t**_**p**_ for a fixed number of beads. Based on these considerations, two scenarios are possible during an unseeded PMSA, as illustrated in Additional file 1: Fig. S13A. The first scenario suggests that the spontaneous misfolding event occurs rapidly and frequently, resulting in a short **t**_**s**_**.** These misfolding events would happen predominantly in the initial stages of the PMSA reaction, overlapping with the propagation process or **t**_**p**_. Consequently, there would be a slow and initially undetectable increase in the amount of rec-PrP^res^ until reaching **t**_**PMSA**_, at which point the rec-PrP^res^ amount turns sufficient to be detectable by total protein staining. Alternatively, in the second scenario represented, spontaneous misfolding would be infrequent and occur at a slower rate resulting in a longer **t**_**s**_. Unlike the first scenario, these would not be a steady increase in rec-PrP^res^ during **t**_**s**_. However, once the initial nuclei are formed (after **t**_**s**_), the propagation process would be very rapid, with a short **t**_**p**_. This phase would involve a rapid and significant increase in rec-PrP^res^ until reaching detectable amounts.

If we consider that one prion-loaded bead represents the minimum amount of spontaneously misfolded rec-PrP^res^ that can be retrieved from a PMSA reaction, we can define the following parameters: **t**_**s**_ as the time required to generate one spontaneously loaded bead; **t**_**p1**_ as the time needed for that single rec-PrP^res^-containing bead to propagate in PMSA until it reaches detectable amounts by total protein staining, and **t**_**pn**_ as the time required for *n* rec-PrP^res^-loaded beads to propagate until they reach detectable amounts. In this case, if **t**_**p1**_** < t**_**PMSA**_, we could conclude that spontaneous misfolding is an infrequent event. When the time required for the propagation of a single nucleus (or single rec-PrP^res^-loaded bead) is shorter than the time required to obtain detectable rec-PrP^res^ in the reaction, it suggests that all the detectable product at the end of the reaction originates from the propagation of that single seed rather than from multiple nuclei. On the contrary, if **t**_**p1**_ or **t**_**pn**_** > tPMSA**, we could conclude that the spontaneous misfolding is a fast and frequent event in PMSA. When the time required for propagation, either from a single or multiple nuclei, is longer than the time needed to obtain detectable rec-PrP^res^ amounts in an unseeded PMSA reaction, it implies that the rec-PrP^res^ observed at the end of the PMSA primarily originates from spontaneous formation and undergoes a subsequent, much slower propagation from each of the generated nuclei.

To assess this, we analyzed the propagation times of 1 versus 30 rec-PrP^res^-loaded 1 mm glass beads in PMSA until detectable rec-PrP^res^ was obtained through PK digestion, electrophoresis, and total protein staining (**t**_**p1**_ and **t**_**p30**_). In parallel, we evaluated the time necessary to detect rec-PrP^res^ in PMSA when using 30 non rec-PrP^res^-loaded 1 mm diameter glass beads (**t**_**PMSA**_). As shown in Additional file 1: Fig. S13B, the experimental procedure involved sampling at 30 min intervals for the propagation PMSA assay and at 90 min intervals for the unseeded PMSA. The presence of rec-PrP^res^ was analyzed by PK digestion, electrophoresis, and total protein staining. According to the study, **t**_**p1**_ and **t**_**p30**_ were both ≤ 0.5 h, indicating that the propagation of recombinant prion from rec-PrP^res^ loaded beads is an extraordinarily rapid process. In contrast, in the unseeded PMSA using the same 30 unloaded glass beads, the minimum reaction time required to detect rec-PrP^res^ (**t**_**PMSA**_) was ≤ 3 h. From these findings, we can infer that the spontaneous formation of rec-PrP^res^ in the presence of 30 glass beads takes approximately 2.5 h. This suggests that the event of spontaneous rec-PrP misfolding leading to the formation of propagation nuclei is relatively infrequent and slow, followed by a very rapid propagation phase.

## Discussion

Despite the fact that the causal agent of TSEs and its pathogenic mechanism was described four decades ago [57], the main event of PrP^C^ to PrP^Sc^ conversion or misfolding, which remains mostly unknown at the molecular level, continues to pose significant challenges. Even after the atomic-level resolution of the three dimensional structures of few prion strains [15, 48, 51], the detailed mechanism by which the globular, natively folded cellular protein transitions into the β-sheet rich structure that ends up forming large amyloid fibrils is yet to be fully described. However, spontaneous misfolding of wild-type PrP^C^ is an extremely rare event, occurring apparently randomly in approximately one individual per million per year [27]. Consequently, it presents extraordinary challenges for in vivo and in vitro modeling. Therefore, there is a pressing need for model systems allowing the systematic study of this pivotal event, which underlies most forms of TSEs. These systems can help address several questions: is spontaneous misfolding a completely stochastic event, or could it be favored/induced/triggered by yet unknown factors? Are cofactors necessary for the event to occur, and if so, how do they interact with PrP? How can different strains arise from the spontaneous misfolding of the same PrP? Are there preferred conformers for a determined PrP sequence? Can the spontaneous formation of such conformations be controlled to obtain a specific strain?

Modelling sporadic, or more precisely, spontaneous idiopathic prion diseases in animal models has been attempted through introduction of *PRNP* mutations associated with human genetic prion diseases, potentially able to increase the proneness of spontaneous misfolding. However, the diseases developed in such transgenic animals have proven to be poorly transmissible, if at all, in wild-type animals [9, 35, 43, 76]. Consequently, they do not faithfully replicate the characteristics of a truly sporadic prion disease. Fortunately, the discovery of bank voles (*Myodes glareolus*) and their high susceptibility to prion diseases led to the first model of sporadic disorders. Although it relies on overexpression, this model consistently exhibits the spontaneous development of a transmissible prion disease. The susceptibility is determined by a specific polymorphic variant that seems to favor the disease when overexpressed [73]. Interestingly, this variant is also naturally occurring in some sheep breeds and has been used to develop a new model for sporadic disease [70]. This is one of the reasons why recombinant bank vole PrP with this variant was chosen for the development of an in vitro system to facilitate the systematic study of spontaneous protein misfolding. Furthermore, we aimed to establish a simple model for investigating such a complex phenomenon, being in vitro prion misfolding or propagation systems the most suitable for this purpose. In this regard, we built upon previous work that initiated with the development of PMCA based on brain homogenates as a source of PrP^C^ [12, 60], which has since evolved over the past decade to incorporate bacterially expressed recombinant PrP and chemically defined cofactors [25, 33, 71]. Therefore, we decided to pursue a similar approach but with an even simpler methodology that would be as robust as PMCA in terms of recombinant prion propagation, namely PMSA [30].

In the light of the infrequent generation of rec-PrP^res^ in unseeded controls or tubes with highly diluted seeds in PMSA, and taking advantage of the simplicity of the system in terms of component number, we envisaged a unique opportunity to develop a robust method for consistent spontaneous prion misfolding and to explore the contributing factors. PMSA for prion propagation is roughly composed by six elements: the recombinant protein, conversion buffer (with salts and detergent), a polyanionic cofactor (dextran sulfate), zirconia silicate beads, shaking, and temperature. Together, these components support efficient prion propagation and occasionally lead to spontaneous rec-PrP misfolding. Hence, our aim was to analyze the contribution of each component to this phenomenon in order to enhance spontaneous misfolding. Initially, we considered increasing the concentration of rec-PrP in the substrate, as observed in overexpressing animals such as transgenic mice overexpressing bank vole PrP, which spontaneously developed a prion disease [73]. However, previous studies investigating the effect of rec-PrP concentration in the PMSA substrate for prion propagation revealed no significant change in the efficiency of the process for concentrations above 1 µM [30]. Thus, and taking into account that with few exceptions such as bank vole PrP, animals overexpressing other wild-type prion proteins do not develop spontaneous *bona fide* prion disease [74], the role of rec-PrP concentration as a critical parameter for enhancing spontaneous misfolding was discarded. The presence of polyanionic and lipidic cofactors in prion propagation substrates has been demonstrated to improve prion propagation efficiency [4, 24] and is considered crucial to obtain infectious recombinant prions [10]. These cofactors have also been proposed to influence strain properties [11, 26, 33], yet their potential contribution to spontaneous misfolding remains uncertain. It is noteworthy that *bona fide* recombinant prions have been generated spontaneously both in the presence [33, 66, 71] and absence [18, 44] of non-protein cofactors, suggesting that their presence is not strictly necessary for the event to occur in vitro. Nonetheless, including these cofactors in substrates aimed to in vitro prion propagation has been shown to enhance propagation efficiency, although this effect could be strain dependent, with each specific cofactor favoring distinct conformers [11]. The likelihood of any polyanionic or lipidic cofactor, among those known to be useful in prion propagation, specifically stimulate spontaneous misfolding is highly unlikely. In a previous work, we used recombinant bank vole protein as a substrate in PMCA, along with various polyanionic cofactors and performed serial unseeded PMCA rounds to generate spontaneously misfolded rec-PrP^res^ with distinct efficiencies. In the absence of cofactors, a minimum of 40 rounds was required for spontaneous rec-PrP^res^ formation. However, the addition of dextran sulfate, RNA or DNA to the substrate significantly accelerated the event reducing the number of required PMCA rounds by at least 12 [33]. This observation indicates that cofactors could overall enhance both spontaneous and induced PrP misfolding. Furthermore, it is worth noting that the electrophoretic patterns of spontaneously generated rec-PrP^res^ were specific for each tested cofactor. These patterns coincided with those resulting from prion propagation when using the same substrates, suggesting that distinct cofactors may specifically promote misfolding into different conformers [33]. This, in turn, could influence the specific promoter effect of each cofactor on the spontaneous misfolding of PrP with different sequences. An illustrative example is the case of dextran, which acts as an enhancer for bovine spongiform encephalopathy prion propagation [53] but as an inhibitor for scrapie prions [29]. In any case, and despite the possibility of introducing a bias in our system due to the potential strain selectivity of a determined cofactor, sulfated dextran was chosen as an overall enhancer for misfolding in the presented PMSA methodology. This choice was made with the objective of developing a system for rapid and consistent spontaneous prion formation *in* vitro, and sulfated dextran was found to be the most consistent cofactor among those previously utilized [33]. The physical reaction parameters, such as temperature and shaking speed, were also evaluated during the optimization of PMSA for prion propagation. Given that a range of temperatures did not show any significant effect on efficiency, it was discarded as a potential major contributor to spontaneous misfolding. In contrast, variations in shaking speed had a profound effect on the efficiency of recombinant prion propagation. Shaking speeds below 700 rpm and above 1000 rpm demonstrated a reduction in propagation efficiency of a serially diluted seed [30]. Since highly efficient in vitro prion propagation methods typically involve sonication or shaking, one might speculate that external energy input is necessary for in vitro PrP misfolding. However, our previous observation of reduced efficiency at high shaking speeds led us to the conclusion that increased energy input is not directly correlated with an enhanced misfolding efficiency. Lastly, it was essential to investigate the role of the beads considering their known contribution to enhanced prion propagation in vitro [32, 39] and the previous observations on recombinant prion propagation in PMSA [30]. Indeed, as shown in Results section, changing the material of beads was the pivotal factor for the transition from a method that exhibited high efficiency in prion propagation but rarely induced spontaneous misfolded rec-PrP^res^ to a method that systematically and remarkably induced spontaneous misfolding with unprecedented efficiency.

Therefore, one of the main inquiries arising from our results is the underlying distinction between glass beads and the other tested materials. Here, we have demonstrated that both native rec-PrP and rec-PrP^Sc^ are adsorbed onto the glass surface of the beads. This leads us to theorize about the potential role of rec-PrP concentration on the glass surface, wherein an increased local concentration of the substrate may enhance the frequency of spontaneous misfolding. Additionally, glass beads could facilitate the association of the few rec-PrP^Sc^ seeds formed spontaneously with rec-PrP or even with the polyanionic cofactor, thereby promoting misfolding. This role can be likened to the proposed function of lipid rafts in vivo [54]. Nonetheless, the involvement of lipid rafts in the pathobiology of prion diseases appears to be more complex, with a closer relationship to their lipidic content rather than solely providing a gathering point for PrP^C^ and PrP^Sc^ [1]. Furthermore, other materials that have been shown to adsorb PrP or prions [31] may do so even more efficiently than glass beads without promoting spontaneous misfolding. An example of this is seen with zirconium silicate beads, which can effectively store recombinant prion seeds adsorbed on their surface but lack the dramatic effect of inducing spontaneous misfolding observed with glass beads. The binding of proteins to different materials, such as plastic tubes compared to glass, has been assessed as early as 1978. These studies demonstrated that proteins exhibit a greater affinity for most of the plastic surfaces than for glass. However, the presence of detergents, such as Triton-X-100 in our conversion buffer, significantly reduces the adherence of proteins to plastics compared to glass [19], which may be relevant to our system. In the specific case of prions, it has been shown that stainless steel wires can efficiently bind PrP^Sc^ [80]. Moreover, when incubated with healthy brain homogenates, stainless steel wires can also to give rise to apparently spontaneously misfolded PrP^Sc^, albeit with a low frequency [28]. This further highlights the potential influence of different materials on prion behavior and misfolding dynamics. However, the use of stainless steel beads in PMSA did not promote spontaneous misfolding to the same extent as observed with glass beads, if at all. The potential adsorption of natively folded PrP to steel surfaces remains to be assessed, and therefore, it is unknown whether steel-induced spontaneous misfolding operates through the same mechanisms as glass-induced misfolding. It is possible that some physicochemical properties unique to glass surfaces are responsible for the highly efficient induction of spontaneous recombinant prion misfolding. Further investigation is needed to elucidate the exact mechanisms underlying the differential effects of glass and steel beads on spontaneous misfolding. The negative surface charge density acquired by glass in aqueous solutions, resulting from the deprotonation of terminal silanol groups [6, 7, 16], suggests the formation of electrostatic interactions between rec-PrP and the glass surface. These interactions not only driving the adsorption process but also having the potential to affect the native structure of PrP. As salt bridges may play an important role in stabilizing the structure of PrP^C^, the disruption of at least a proportion of these salt bridges is likely required for misfolding to occur [40]. Thus, it is plausible that the contribution of the glass surface to the event consists of a partial destabilization of the native PrP conformation, thereby promoting spontaneous misfolding. However, this hypothesis alone does not fully explain the observed results. It should be noted that, in addition to the glass surface, bead movement was also found to be necessary for highly efficient spontaneous rec-PrP misfolding. Assuming the existence of a significant energy barrier that prevents the misfolding of PrP^C^ to PrP^Sc^ [79], the destabilization of the native structure of PrP^C^ through its interaction with the glass surface may serve to lower this barrier by increasing the free energy of PrP^C^ to some extent. Consequently, the glass-bound PrP would become an energetically unstable intermediate along the misfolding pathway, resembling the proposed concept of PrP* [20]. However, an additional energy input would still be required to overcome the energetic barrier from PrP^C^ (or PrP*) to PrP^Sc^, and this energy could potentially be provided by the movement of the beads. A speculative possibility would be that the kinetic energy generated by the movement of the beads could be transferred to the tightly bound rec-PrP on their surface. As the mass of the bead combines with the mass of the rec-PrP molecule adsorbed to its surface, the additional kinetic energy could facilitate overcoming the energetic barrier. Further studies aimed at elucidating the molecular mechanisms driving this phenomenon in vitro are of great interest, since they could potentially provide insights into equivalent phenomena occurring in vivo. Such understanding would be crucial in unraveling the triggers of rare sporadic prion disorders.

Another interesting observation worth discussing and potentially related to the essential components for spontaneous PrP misfolding is the possible requirement of rec-PrP presence in solution. While analyzing the impact of glass surface area on spontaneous rec-PrP^Sc^ formation, the need of a minimum surface area was clearly established. However, due to technical limitations, whether a maximum surface area exists could not be precisely determined. However, in experiments performed with an excess of glass beads (with estimated surface areas of 2500π mm^2^), no spontaneous rec-PrP^res^ formation was observed. This suggests the existence of an upper limit to glass surface. In the light of this observation and the remarkable capacity of glass surfaces to adsorb rec-PrP, we hypothesize that with extremely high glass surfaces available during the reaction, there could be a rapid and complete depletion of rec-PrP in solution, with all the rec-PrP adsorbed onto the surface. The requirement for soluble or non-bead surface-bound rec-PrP for highly efficient spontaneous conversion is reminiscent of the higher efficiency of GPI-less PrP in propagating prions in vivo compared to membrane-bound PrP. This GPI-less PrP has shown higher levels of PrP^res^ despite a reduced neurotoxicity [17]. Furthermore, studies on transgenic mice expressing anchorless PrP have demonstrated the development of spontaneous disease in approximately 50% of the cases, with long incubation periods. However, when GPI-anchored PrP was co-expressed at low levels (0.5 x) with the GPI-less protein, spontaneous disease onset occurred in 100% of the animals with much shorter incubation periods [64]. These findings further suggest that the presence of both membrane-bound and anchorless PrP somehow benefits spontaneous misfolding in vivo, similar to the potential interplay between glass bead-bound and soluble rec-PrP in PMSA. Taking this into account, and the evidences indicating that spontaneous misfolding is an infrequent event that might be associated to the glass surface, we propose a model of this highly spontaneous conversion system, shown in a short animation included as Additional file 2.

Apart from the system developed, which holds invaluable potential for gaining insights into the mechanisms triggering spontaneous PrP misfolding and the influence of cofactors, PrP sequence variations, or other physicochemical factors in this process, the spontaneously generated products in this system are of significant importance. Although some of our previous work suggests that distinct cofactors could restrict or modulate the formation or selective propagation of specific strains [33], even in the presence of different seeds [26], we now report the generation of at least four different strains through the same procedure and with the use of the same polyanionic cofactor. This occurrence is not unprecedented, as the arousal of different prion conformers from the same procedure, employing the same cofactor, has been demonstrated previously [67, 77]. This phenomenon could also be happening in vivo, as coexistence of different strains within the same patient have been estimated to occur in 35% of sCJD cases [56]. Although the cofactor selection model [65] suggests that this may be due to the presence of distinct cofactors in different brain areas, which determine the occurrence of specific strains, the discovery of mixed strains within a single brain area [45], implies the involvement of other mechanisms in the formation of multiple strains. In this study, we show that even in a simple experimental system, multiple strains can emerge from the same procedure, although differences in their formation frequencies suggest the existence of a preferred conformer for the specific combination of rec-PrP and cofactor. Such method, able to reproduce in vitro the spontaneous formation of prions with accelerated kinetics and giving rise to multiple strains, offers several possibilities to address questions underlying idiopathic prion disorders. Among others, we are currently expanding the potential of the system to analyze the spontaneous misfolding proneness of hundreds of distinct PrP variants in order to decipher the motifs or residues facilitating this event. Another example of the potential of the method is the investigation of PrP-cofactor interactions driving this process. Distinct, ad hoc designed polyanionic cofactors promoting spontaneous PrP misfoding can be easily screened through PMSA, what could help unveil the necessary cofactor-PrP interactions. Given the potential role of polyanionic cofactors in prion disease, in which for example, shortening of heparan sulfate chains from brain prolongs survival in prion infected animals [2], these kind of compounds from brain could have also an effect on the pathobiology of sporadic prion disorders. The method presented here could help unveil any potential PrP-cofactor interactions potentially leading to the development of a sporadic prion disease.

Altogether, we present here the first method that consistently induces spontaneous misfolding of *bona fide* recombinant prions. This misfolding is primarily facilitated by the addition of glass beads to the PMSA reaction, which, likely through electrostatic interactions with the rec-PrP, significantly enhance their proneness to spontaneous conversion into highly infectious and transmissible prions. Furthermore, our method generates multiple prion strains under identical conditions, including the use of the same cofactor molecule. These findings challenge existing theories regarding the role of cofactors in prion strain selection or emergence, highlighting the potential of our method to explore complex aspects such as the triggers for spontaneous prion misfolding and the involvement of cofactors and other elements in this process.

### Supplementary Information

**Additional file 1.** Supplementary figures, tables and methods.

**Additional file 2.** Descriptive video of the PMSA procedure developed for the generation of spontaneous protein misfolding
