# Supplementary material for: Understanding the key features of the spontaneous formation of bona fide prions through a novel methodology that enables their swift and consistent generation
Source: Acta Neuropathol Commun. 2023 Sep 7;11:145. doi: 10.1186/s40478-023-01640-8 (PMC10486007; doi:10.1186/s40478-023-01640-8)
Supplement: Supplementary file 1 — Additional file 1. Supplementary figures, tables and methods. [file 40478_2023_1640_MOESM1_ESM.docx]

**Supplementary material**

**Understanding the key features of the spontaneous formation of *bona fide* prions through a novel methodology that enables their swift and consistent generation**

Hasier Eraña^1,2,3^, Carlos M. Díaz-Domínguez^1,3^, Jorge M. Charco^1,2,3^, Enric Vidal^4^, Ezequiel González-Miranda^1^, Miguel A. Pérez-Castro^1^, Patricia Piñeiro^1^, Rafael López-Moreno^1^, Cristina Sampedro-Torres-Quevedo^1^, Leire Fernández-Veiga^1^, Juan Tasis-Galarza^1^, Nuria L. Lorenzo^5^, Aileen Santini-Santiago^1^, Melisa Lázaro^1^, Sandra García-Martínez^1^, Nuno Gonçalves-Anjo^1^, Maitena San-Juan-Ansoleaga^1^, Josu Galarza-Ahumada^1^, Eva Fernández-Muñoz^1^, Samanta Giler^4^, Mikel Valle^1^, Glenn C. Telling^6^, Mariví Geijó^7^, Jesús R. Requena^5^ and Joaquín Castilla^1,3,8^

**^1^** Center for Cooperative Research in Biosciences (CIC bioGUNE), Basque Research and Technology Alliance (BRTA), Bizkaia Technology Park, 48160 Derio, Spain.

**^2^** ATLAS Molecular Pharma S. L. Bizkaia Technology Park, 48160 Derio, Spain.

**^3^** Centro de Investigación Biomédica en Red de Enfermedades infecciosas (CIBERINFEC), Carlos III National Health Institute, 28029 Madrid, Spain.

**^4^** IRTA. Programa de Sanitat Animal. Centre de Recerca en Sanitat Animal (CReSA). Campus de la Universitat Autònoma de Barcelona (UAB), Bellaterra, Catalonia. Spain.

**^5^** CIMUS Biomedical Research Institute & Department of MedicalSciences, University of Santiago de Compostela-IDIS, 15782 Santiago de Compostela, Spain.

**^6^** Prion Research Center (PRC), Colorado State University, Fort Collins, Colorado 80523, USA.

**^7^** Animal Health Department, NEIKER-Basque Institute for Agricultural Research and Development. Basque Research and Technology Alliance (BRTA), Bizkaia Technology Park, 48160 Derio, Spain.

**^8^** IKERBASQUE, Basque Foundation for Science, 48011 Bilbao, Spain.

* To whom correspondence should be addressed:

Joaquín Castilla

CIC bioGUNE

Parque tecnológico de Bizkaia

Derio 48160, Bizkaia, Spain

E-mail: [jcastilla@cicbiogune.es](mailto:jcastilla@cicbiogune.es)


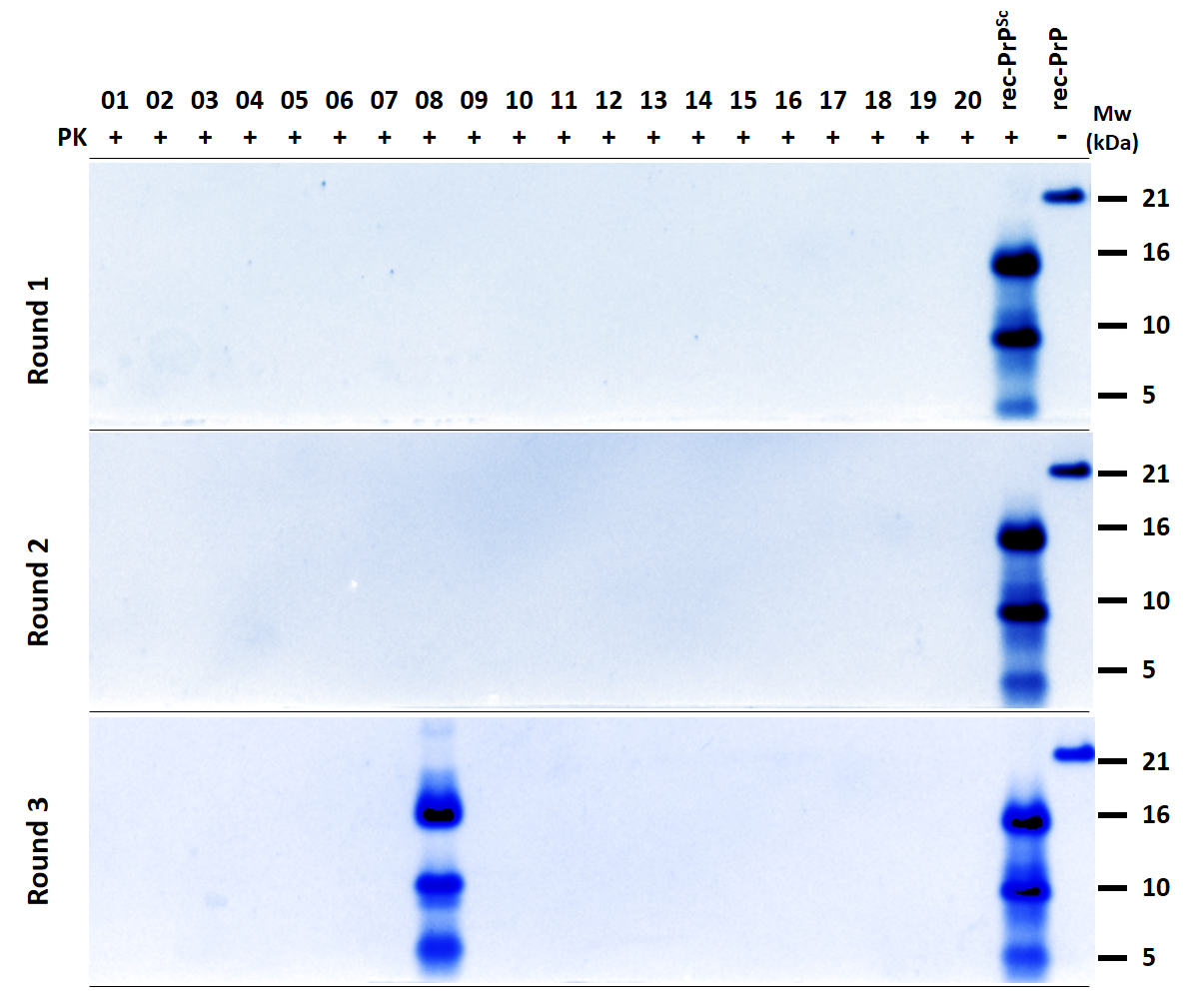


**Supplementary figure 1. Assessing the frequency of spontaneous bank vole rec-PrP^res^ formation by PMSA in the presence of zirconia silica beads.** Twenty independent unseeded PMSA reaction tubes were prepared, supplemented with 1 mm zirconia silicate beads, and subjected to three consecutive 24 h rounds of serial dilutions (1:10 between rounds). PMSA products from each round were treated with proteinase K (PK) at 25 µg/ml for 1 h at 42 ˚C. To enhance detection sensitivity, the products were concentrated approximately 50 times by centrifugation and visualized through electrophoresis and total protein staining. Bank vole rec-PrP^res^ was detected in only one tube during the third round of PMSA (5%). rec-PrP^Sc^: positive control, seeded with L-seeded-PMSA recombinant prion strain, previously adapted to PMSA [1]. rec-PrP: Undigested substrate. Mw: Molecular weight marker.


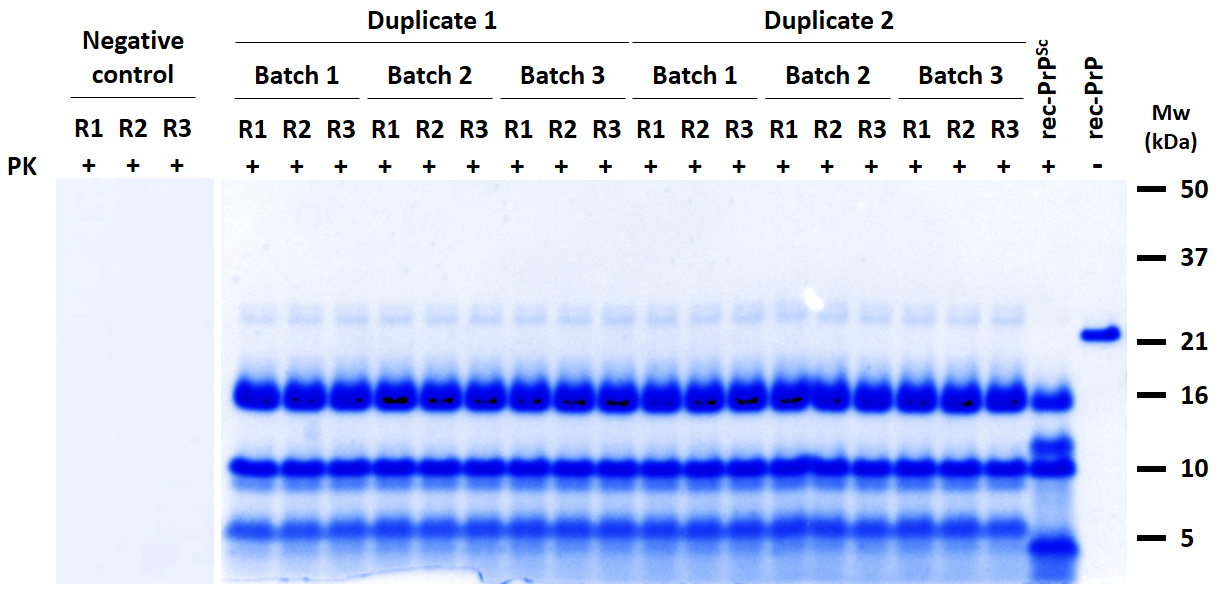


**Supplementary figure 2.** **Assessing the spontaneous formation of prions by PMSA using glass beads and rec-PrP from different batches in a prion-free laboratory.** Three different rec-PrP batches were used to prepare PMSA substrates, each supplemented with newly opened 1 mm glass beads, to evaluate the reproducibility of the spontaneous misfolding in PMSA and rule out cross-contamination. The experiment was conducted in duplicate within a prion-free laboratory using prion-free equipment. Additionally, substrate done with batch 1 protein was used also as negative control, using 1 mm zirconia silicate beads in those tubes instead of glass beads. The substrates, based on bank vole rec-PrP, were subjected to three consecutive 24 h rounds of PMSA (R1 to R3, 1:10 dilutions). PMSA products from each round were treated with proteinase K (PK) at 25 µg/ml for 1 h at 42 ˚C. All samples were concentrated approximately 50 times to improve the detection limit and were visualized by total protein staining. Bank vole PrP^res^ was detected in all batches starting from the first round of PMSA. rec-PrP^Sc^: L-seeded-PMSA recombinant prion strain used as control [1]. rec-PrP: Untreated substrate. Mw: Molecular weight.


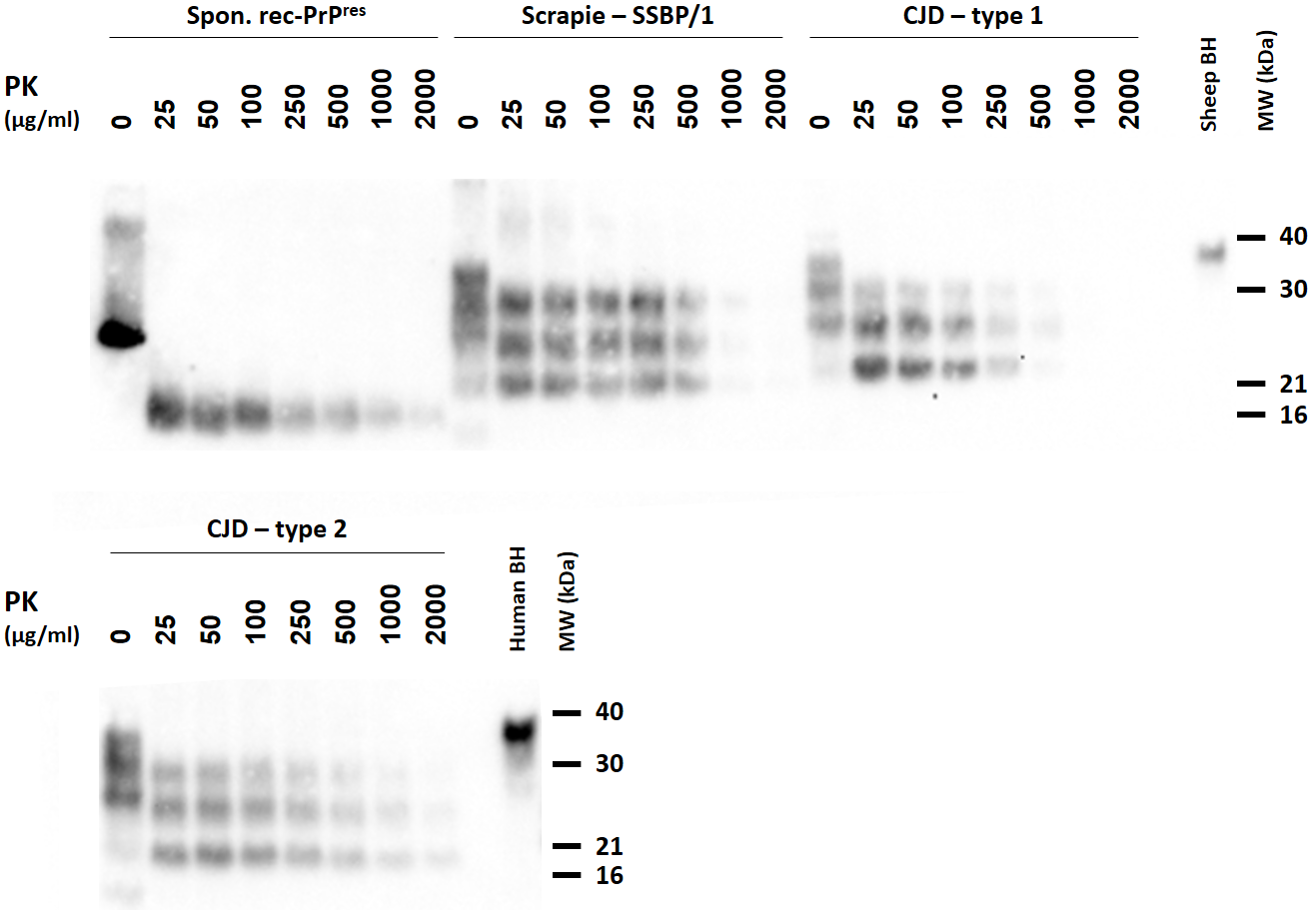


**Supplementary figure 3. Relative proteinase K resistance of spontaneously generated rec-PrP^res^ compared to three brain-derived prion strains.** The spontaneously generated rec-PrP^res^ from PMSA was mixed with 10 % PrP-KO transgenic mouse brain homogenate to mimic the sample environment, along with 10 % homogenates from prion-affected sheep brains (SSBP/1 strain), and humans (two different subtypes of Creutzfeldt-Jakob Disease, CJD, types MM1 and VV2). Total protein amount in all samples was measured using the BCA assay and adjusted to 1.9 mg/ml by diluting more concentrated samples with PBS. Following the concentration adjustment, samples were digested proteinase K (PK) concentrations ranging from 25 to 2000 µg/ml for 1 h at 42 ˚C and 450 rpm shaking. Subsequently, the digested samples were visualized by Western blotting using the 9A2 monoclonal antibody (dilution 1:4000). Undigested sheep and human brain homogenates (BH) were included as size references. The PMSA product demonstrated resistance to digestion with up to 2000 µg/ml of PK, similar to subtype 2 Creutzfeldt-Jakob disease causing prions (CJD-type 2) and slightly higher than subtype 1 Creutzfeldt-Jakob disease causing prions (CJD-type 1), as well as the sheep scrapie SSBP/1 strain. MW: Molecular weight marker.


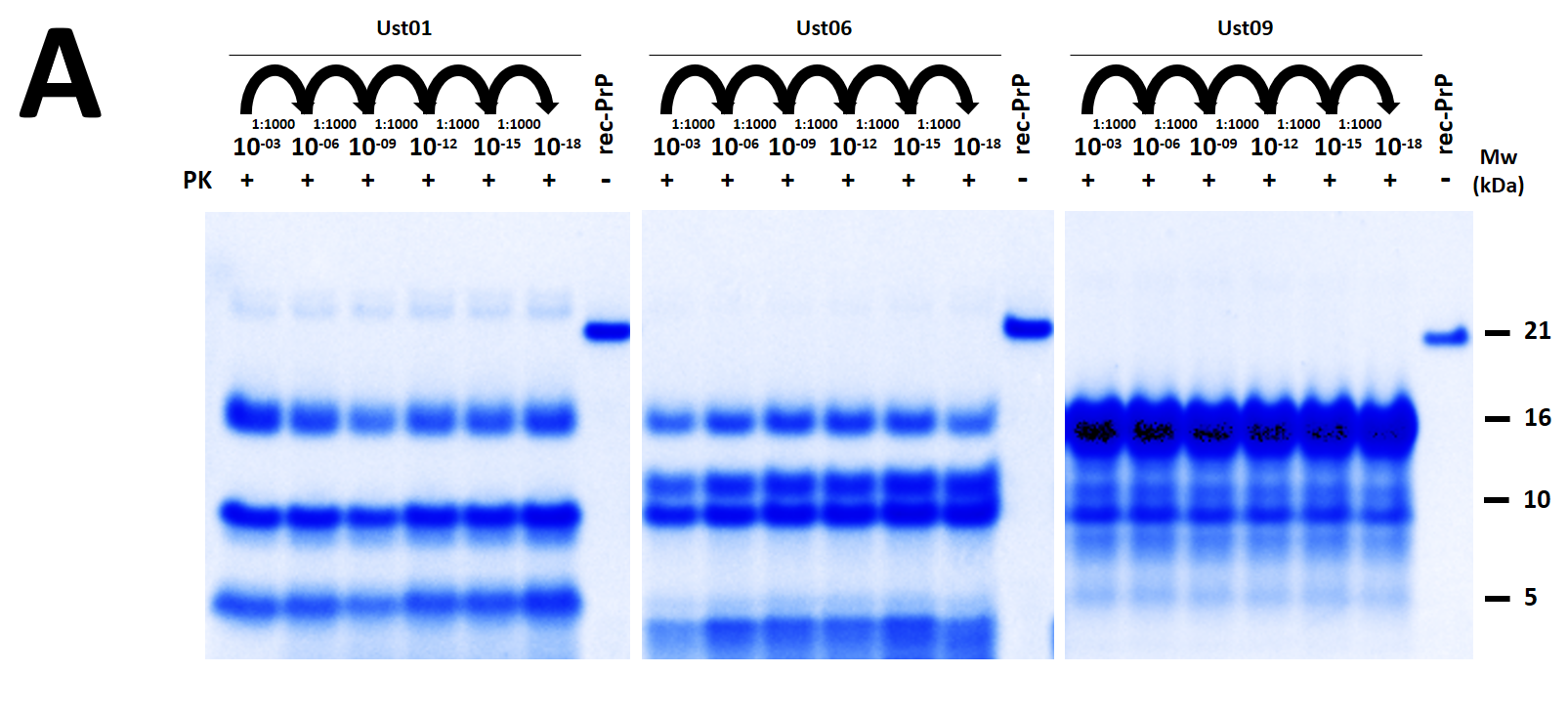


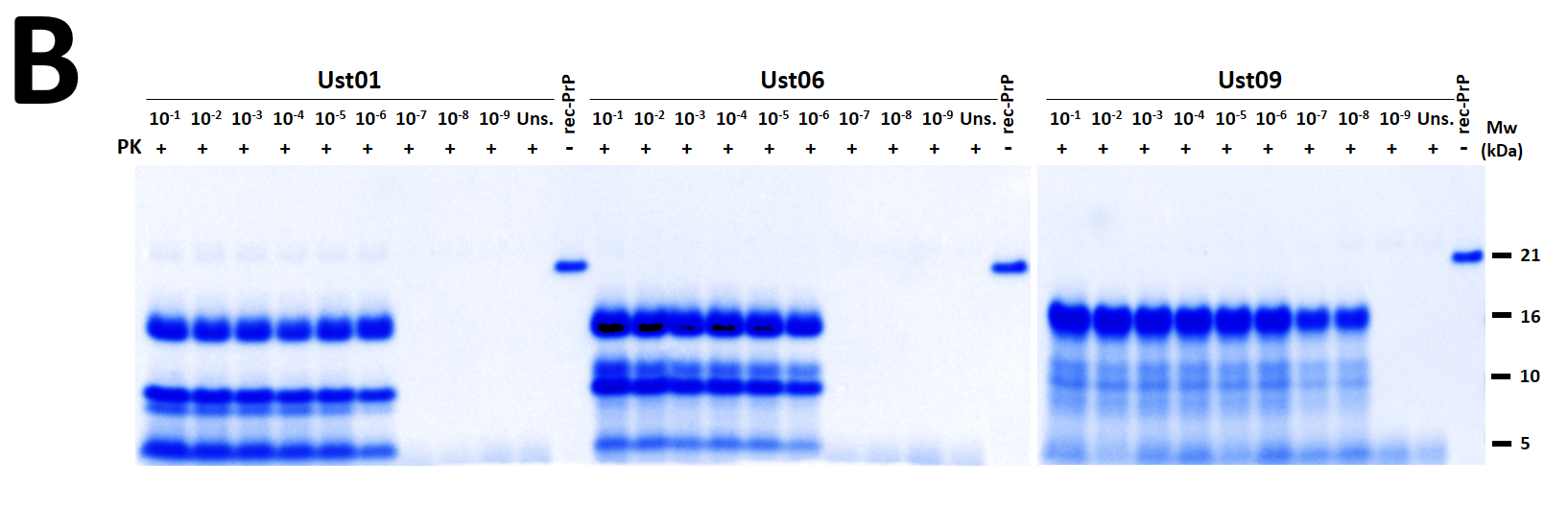


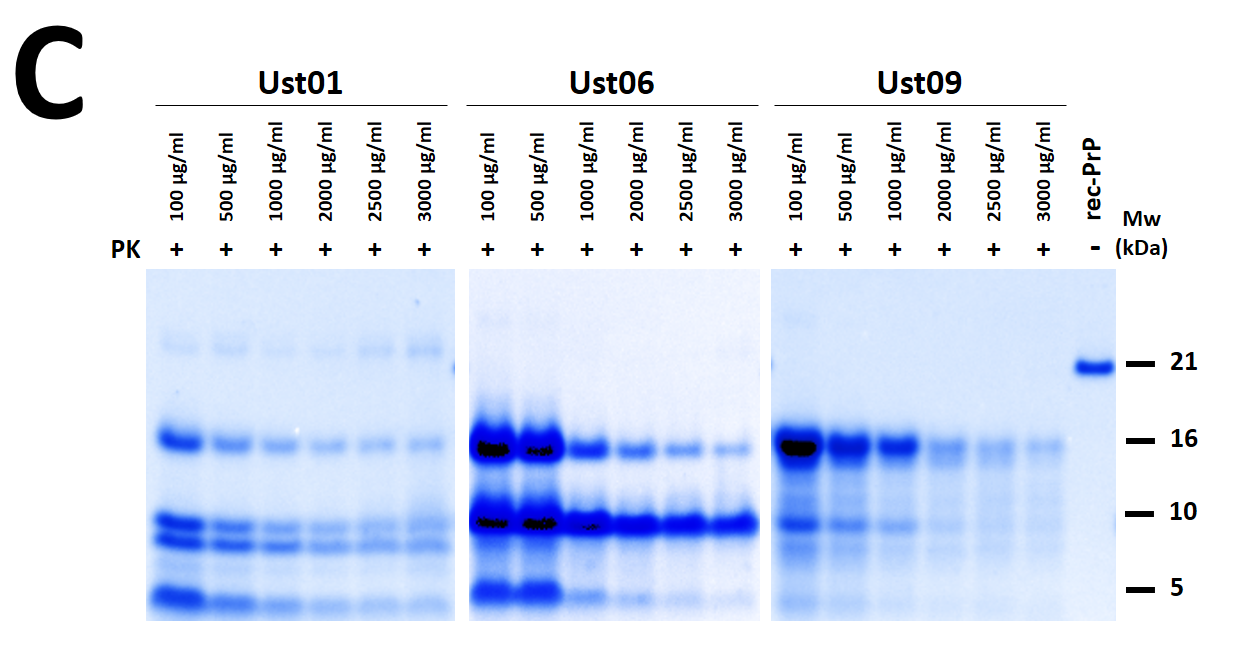


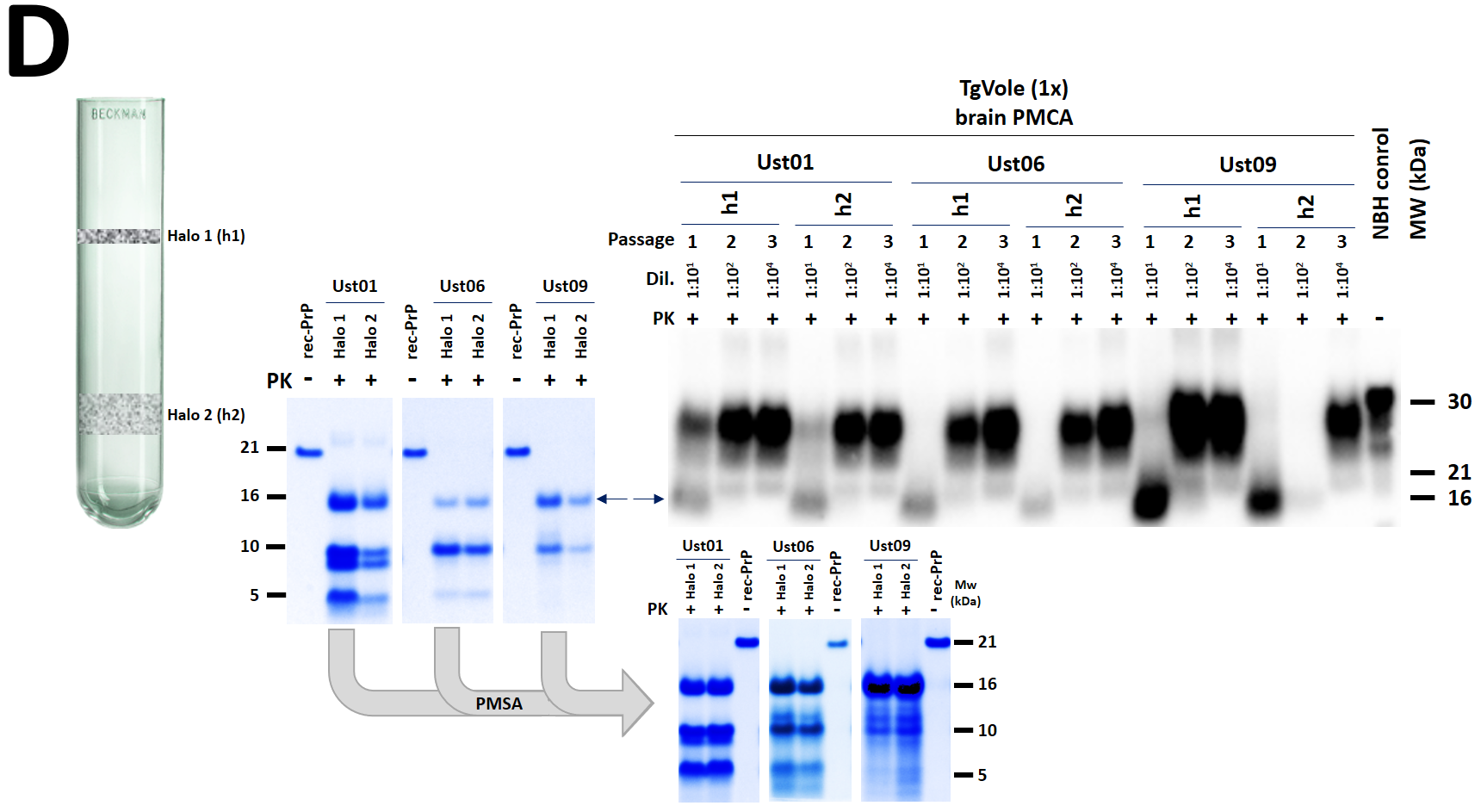


**Supplementary figure 4. Biochemical analysis of spontaneously generated misfolded rec-PrP^res^ with distinctive electrophoretic patterns to determine the fulfillment of key characteristics expected for *bona fide* prions. A) Evaluation of self-propagation ability by serial PMSA passages.** Spontaneously generated PMSA products with distinct electrophoretic patterns, different from the previously analyzed Ust02, were stabilized and loaded into zirconium silicate beads for storage. These products were used to seed a fresh PMSA substrate at a 1:10 dilution, supplemented with 1 mm zirconia silicate beads, and subjected to a 24 h PMSA round. A total of 10 PMSA rounds, using a 1:10 dilution of the previous round as the seed, were performed. All reactions included 1 mm zirconia silicate beads to promote propagation without spontaneous misfolding. The samples were concentrated approximately 50 times to improve detection and visualized by total protein staining. Stable and successful propagation was indicated by the preservation of the original electrophoretic migration profiles after PK digestion. **B)** **Evaluation of the self-propagating ability by serial dilutions in PMSA.** The spontaneously generated PMSA products (before loading into zirconium silicate beads for storage) were serially diluted from 10^-1^ to 10^-9^ in fresh substrates, supplemented with 1 mm zirconia silicate beads, and subjected to a single 24 h PMSA round. An unseeded sample served as a negative control. The samples were concentrated approximately 50 times for improved detection and visualized through total protein staining. After PK digestion, PrP^res^ was detectable up to a dilution of 10^-6^ for conformers Ust01 and Ust06, and 10^-8^ for Ust09, indicating high efficiency in *in vitro* propagation. **C) Evaluation of the proteinase K resistance of the spontaneously generated rec-PrP^res^.** The proteinase K resistance of the spontaneously generated misfolded rec-PrP, with distinct electrophoretic patterns, was assessed using the products obtained from the serial PMSA propagations mentioned above. The samples were digested with increasing concentrations of PK, ranging from 100 µg/ml to 3000 µg/ml at 42 ˚C for 1 h. The rec-PrP^res^ propagated from the spontaneously generated seeds exhibited high resistance, withstanding up to 3000 µg/ml at 42 ˚C, fulfilling another characteristic commonly observed in brain-derived prions and showing similar resistance to the previously analyzed Ust02 conformer. **D)** **Assessment of the capacity of distinct recombinant misfolded PrP to induce misfolding of PrP^C^ from brain *in vitro*.** To predict the potential infectivity *in vivo* of the distinct recombinant misfolded PrP spontaneously generated in PMSA (referred to as Ust01, Ust06, and Ust09), their capacity to induce misfolding of PrP^C^ in brain homogenates of TgVole 1x animals was evaluated using PMCA. Aggregates were purified by ultracentrifugation through a density gradient, resulting in two visible halos of proteic aggregate (Halo 1, h1, and Halo 2, h2) in all three samples. These purified fractions exhibited indistinguishable biochemical properties after proteinase K digestion and retained the same electrophoretic pattern as the original product. These purified fractions were used to seed a PMCA substrate based on TgVole 1x brain homogenate at 1:10 dilutions, and a 24 h PMCA reaction was performed. Two additional serial PMCA rounds were conducted, with the second round seeded at 1:10 dilution using the product from the first round, and the third round utilizing a 1:100 dilution of the product form the second one. After the three serial PMCA rounds of 24 h, PrP^Sc^ detection was carried out by proteinase K digestion (85 µg/ml, for 1 h at 42 ˚C) and Western blotting (using mAb Sha31 at a dilution of 1:4000). Whereas both fractions from the Ust01 seed yielded PrP^Sc^ from the first PMCA round, the Ust06 fractions, required up to two rounds to induce misfolding of brain PrP^C^. The halo 1 and halo 2 fractions from Ust09 exhibited slightly different behavior, with h1 inducing PrP^C^ misfolding from the first PMCA round and the h2 requiring three rounds, although the difference may be attributed to the varying amounts of rec-PrP^res^ detected in each fraction. Nonetheless, all seeds demonstrated the capacity to induce PrP^C^ misfolding, resulting in the classical three-banded PrP^Sc^ pattern, suggesting the potential infectivity of these preparations *in vivo*. Furthermore, the capacity of the same fractions to propagate on rec-PrP-based PMSA substrate was assessed by PMSA. Both halos of each product were used as seeds at a 1:10 dilution on a substrate supplemented with zirconium silicate beads. Following a single 24 h PMSA round at 39˚ C and 700 rpm, the presence of rec-PrP^res^ was evaluated through proteinase K digestion, electrophoresis, and total protein staining. All fractions were able to propagate in PMSA, with no detectable differences and maintaining the original electrophoretic patterns of the PMSA products. Uns.: Unseeded. rec-PrP: Untreated substrate. NBH control: Normal brain homogenate from TgVole 1X. Mw: Molecular weight.


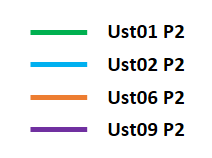

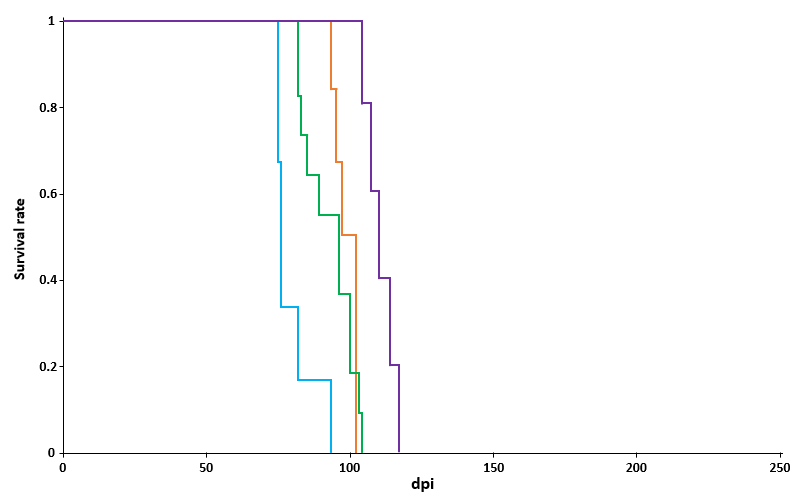

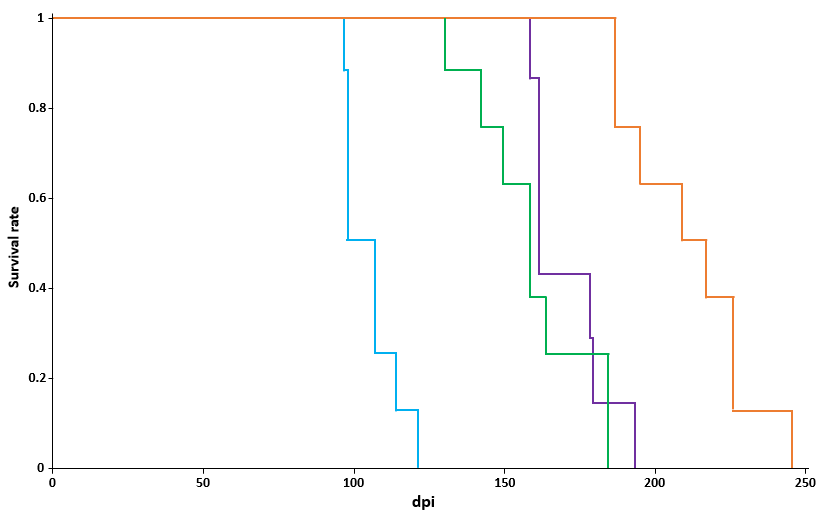

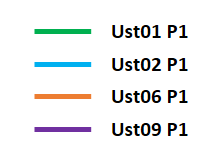


**B**

**A**

**Supplementary figure 5. Kaplan-Meier plots depicting the survival rates of TgVole 1x animals following intracerebral inoculation with spontaneously misfolded recombinant PrP preparations. A) Survival rates of TgVole 1x animals after intracerebral inoculation Ust01, Ust02, Ust06, and Ust09 recombinant preparations.** The PMSA products, characterized by distinct electrophoretic migration profiles, were diluted 1:10 in PBS and intracerebrally inoculated into groups of 6-8 TgVole 1x animals. All preparations resulted in a 100 % attack rate, but they exhibited different incubation periods upon the first passage. The Ust02 preparation had the shortest incubation period, while Ust01 and Ust09 had intermediate periods, and Ust06 had the longest. **B) Survival rates of TgVole 1x animals during secondary transmission of Ust01, Ust02, Ust06, and Ust09 recombinant prions.** For the second passage, brain homogenates from diseased TgVole animals in each of the previous groups were selected based on their incubation time close to the mean and the intensity of their PrP^Sc^ signal in Western blotting. The brain homogenates were prepared at 10 % (w/V) in PBS with protease inhibitor cocktail and diluted 1:10 in PBS before intracerebral inoculation. Once again, all isolates resulted in 100 % attack rate. As expected, the incubation times were shortened compared to the first passage, but the overall trends remained consistent, with Ust02 exhibiting the shortest incubation time, followed by Ust01, Ust06, and Ust09. Dpi: days post-inoculation.


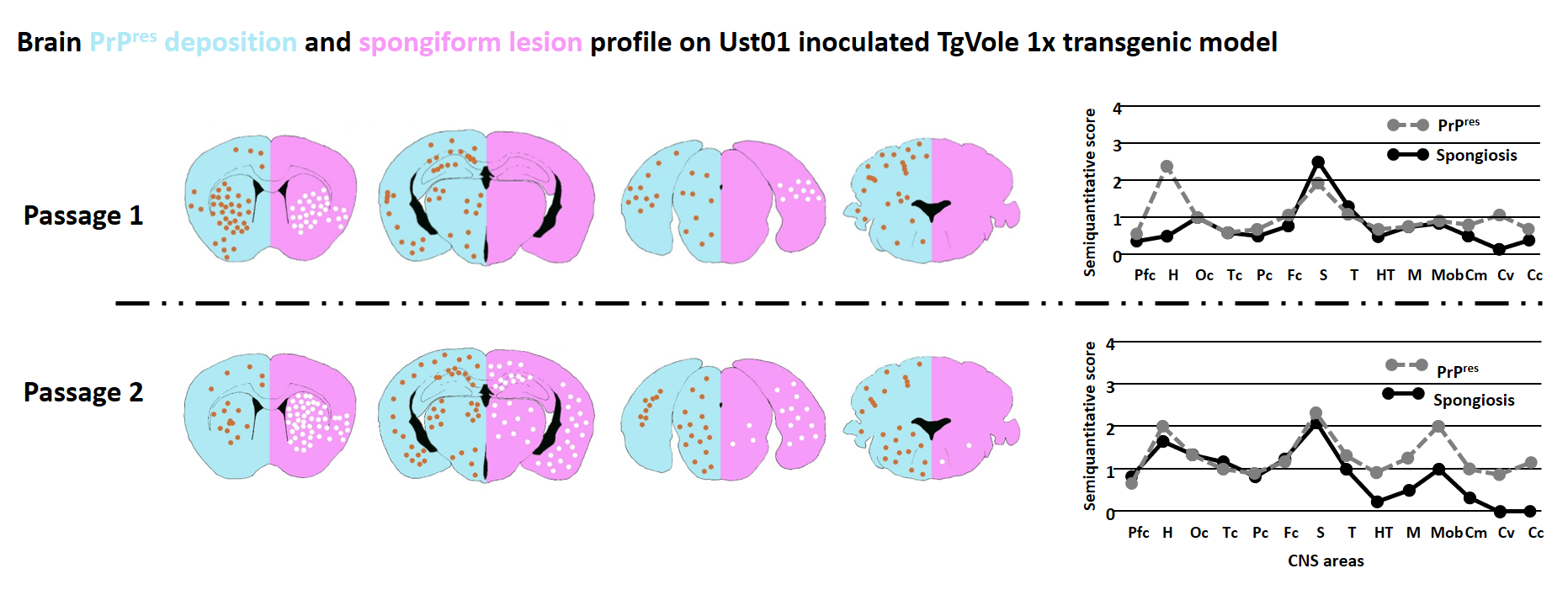


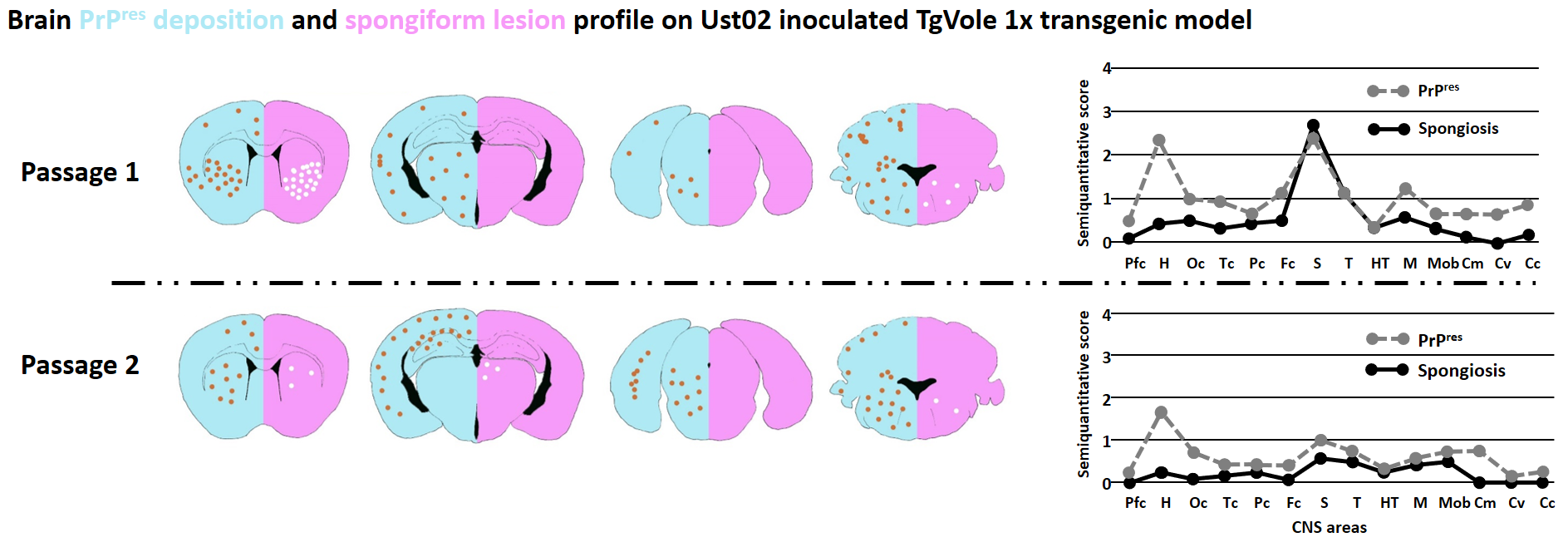


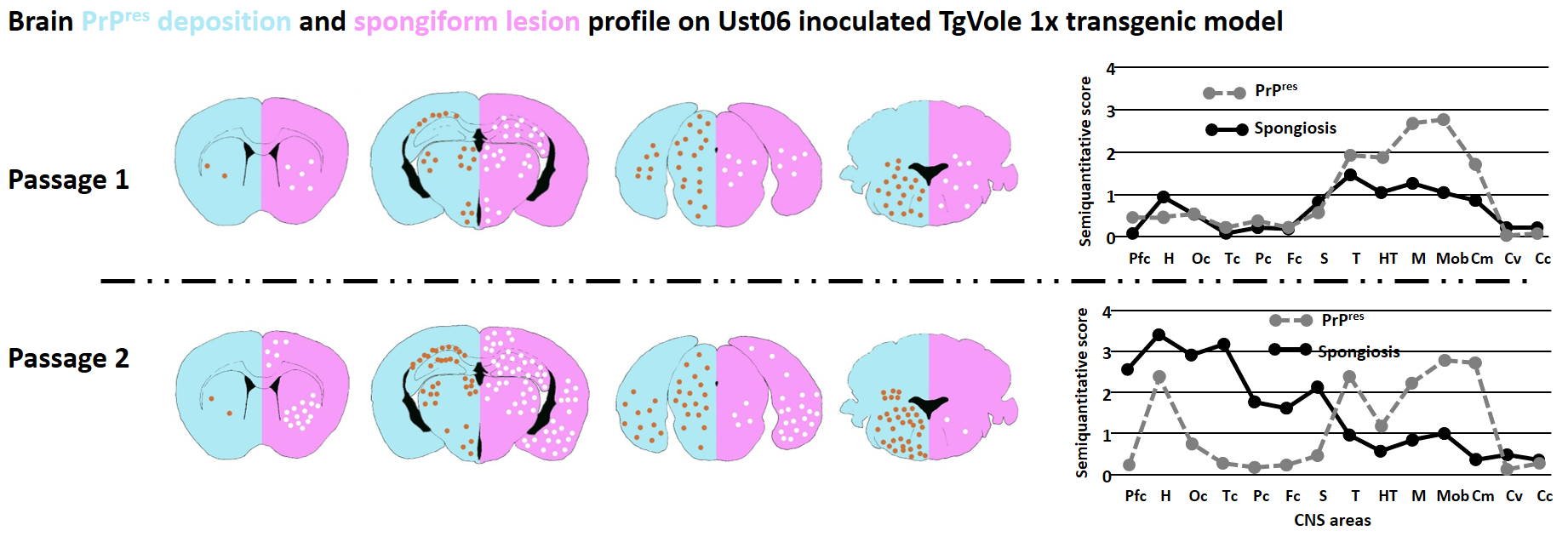


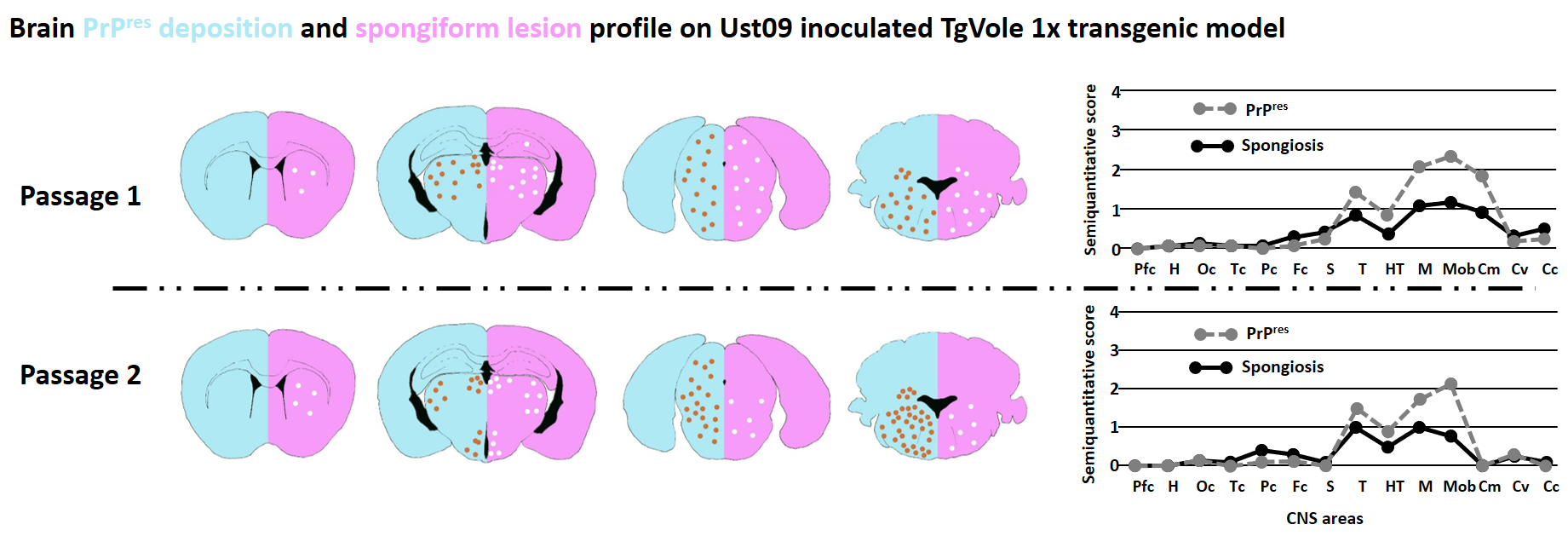


**Supplementary figure 6. Lesion profiles from the first and second passages of TgVole 1x animals inoculated with recombinant prions Ust01, Ust02, Ust06, and Ust09.** An exhaustive histopathological analysis of formalin fixed paraffin embedded half-brains that included 14 different brain areas (Pfc: piriform cortex, H: hippocampus, Oc: occipital cortex, Tc: temporal cortex, Pc: parietal cortex, Fc: frontal cortex, cc: corpus callosum; S: striatum, T: thalamus, HT: hypothalamus, M: mesencephalon, Mob: medulla oblongata, Cm: cerebellar nuclei, Cv: cerebellar vermis, Cc: cerebellar cortex) from TgVole 1x animals at terminal stage of disease was performed, in order to determine whether the potentially different recombinant preparations show different biological characteristics and could be thus, considered distinct strains. The graphical representations illustrate the localization and abundance of spongiform lesions (continuous line, black) and PrP^res^ deposit distribution (dashed line, grey) for each recombinant prion preparation. The scoring is based on a semi-quantitative scale (0-4, vertical axis). Spongiform change was evaluated using hematoxylin & eosin staining on formalin-fixed paraffin embedded brain sections, while PrP^res^ deposits were detected using the 6C2 (1:1000) monoclonal anti-PrP antibody in serially obtained sections. Upon first passage, Ust01 and Ust02 show similar lesion profiles. However, upon secondary transmission, all four recombinant prions exhibit clearly distinguishable profiles, indicating the presence of different prion strains.

**Specific infectivity *in vivo*, Lethal dose 50 (LD_50_) calculation for Ust02 recombinant prion**

Titration of prion infectivity in a particular inoculum through a specific bioassay for brain-derived prions is usually expressed as LD50/g of brain tissue (Lethal dose 50/g of tissue, meaning the necessary amount of infected tissue to cause clinical disease in 50 % of inoculated animals) [2]. Alternatively, in occasions, for *in vitro* prion propagation procedures, as LD50/µl of inoculum [3] that can be translated easily to LD50/g of tissue. Although this is widely accepted in the field, correlating titer with amount of tissue is not directly referring to the titer of the infectious agent, which would be the LD50 per amount of misfolded PrP (PrP^Sc^). However, this value, the amount of misfolded PrP within a tissue, is difficult to determine precisely. On the other hand, for recombinant prion preparations this is normally calculated as LD50/µg of PrP [4]. In order to compare brain-derived and recombinant prion preparations, specific infectivity, should be in both cases calculated as LD50 per amount of misfolded PrP, that some authors approach by considering protease-resistant PrP or PrP^res^ as the infectious entity, and thus, normalizing LD50 values per amount of PrP^res^ measured by PK digestion of the inoculum, immunoblotting and densitometry analysis. Nonetheless, the existence of protease-sensitive infectious entities [5], together with the relative resistance to PK of different isolates, which depends not only on PrP:PK ratio but also on the presence of other proteins and biomolecules in the mixture, led us to discard this approach. Therefore, the comparison of specific infectivity of brain-derived prions and that of recombinant prions needs to rely on several assumptions. Since the nature of the infectious prions is yet unclear in terms of PK-resistant and sensitive misfolded PrP species, we consider that the most reliable approach is to compare infectivity with respect of misfolded PrP, either in brain or in PMSA reaction products containing recombinant PrP (assuming it is mostly PrP^Sc^ or rec-PrP^Sc^). In this case, we could assume a conversion yield for the reaction for recombinant prion generation close to 100 %, based on precipitation experiments and quantification of total PrP in supernatant and pellet [1]. This consideration is the most conservative possible and impedes over-estimating infectious titers, as it considers that all the inoculated material corresponds to infectious prions. This would allow the most cautious estimation of specific infectivity in terms of LD50/µg of PrP^Sc^ or more specifically misfolded recombinant PrP. Similarly, the calculation for brain-derived prions would require an estimation of the amount of PrP^Sc^ present in the brain of a terminally ill animal per g of tissue. This data can be found in literature for some brain-derived prion strains, being the most precise quantitation, that of terminally ill hamsters after inoculation with 263k strain performed by Mass Spectrometry [6], which is of approximately 20 µg of PrP^Sc^/g of brain tissue. To perform these calculations, dilutions 10^-2^, 10^-3^, 10^-4^, 10^-5^ and 10^-6^ of Ust02 prions were also inoculated intracerebrally in TgVole models, as was done with the 10^-1^ dilution for the assessment of their infectious capacity. Again, animals were culled upon manifestation of signs of neurological disease, and brains harvested to evaluate presence of hallmarks of prion infection, mainly through Western blotting of brain homogenates after proteinase K digestion for PrP^Sc^ detection. With comparative purposes, inoculations of serial dilutions of a brain-derived bank vole prion strain was also included, CWD-vole, being the fastest prion disease described to date in animal models of TSE [2].

**Supplementary table 1.** **Results from intracerebral inoculation of serially diluted recombinant bank vole Ust02 prions and CWD-vole prions in TgVole (1x) mice to calculate specific infectivity.**


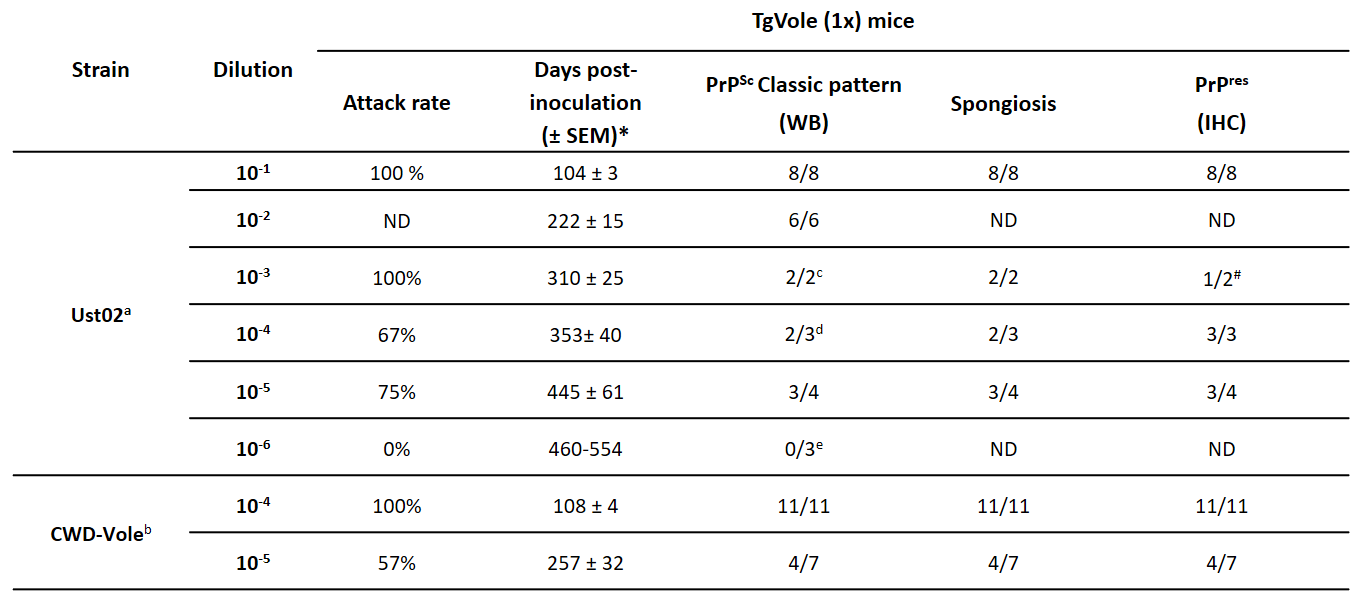


^a^ Recombinant inoculum was prepared diluting the PMSA product 1:10 in PBS, which was afterwards serially diluted 1:10 up to dilution 10^-6^ of the PMSA product. 20 µl of the diluted inocula were inoculated intracerebrally in each TgVole mouse.

^b^ Brain homogenate of a CWD-infected bank vole (at terminal stage of disease), was homogenized at 10 % (w/v) in PBS (with protease inhibitor cocktail) and afterwards serially diluted 1:10 in PBS up to dilution 10^-5^. 20 µl of the diluted inoculum were injected intracerebrally in each TgVole mouse.

^c^ 2 animals from this group died with intercurrent disease and were excluded from the study.

^d^ 1 animal from this group died with intercurrent disease and was excluded from the study.

^e^ 1 animal from this group died with intercurrent disease and was excluded from the study.

* Incubation periods were calculated taking into account only those animals from each group showing unequivocal signs of prion disease, that is, positive for either PrP^Sc^ by WB, spongiosis or PrP^res^ by IHQ.

# Despite PrP^res^ could not be detected by IHQ in one of the animals of the group, it was considered positive due to the detection of spongiosis and PrP^Sc^ by WB.

ND: Not determined.


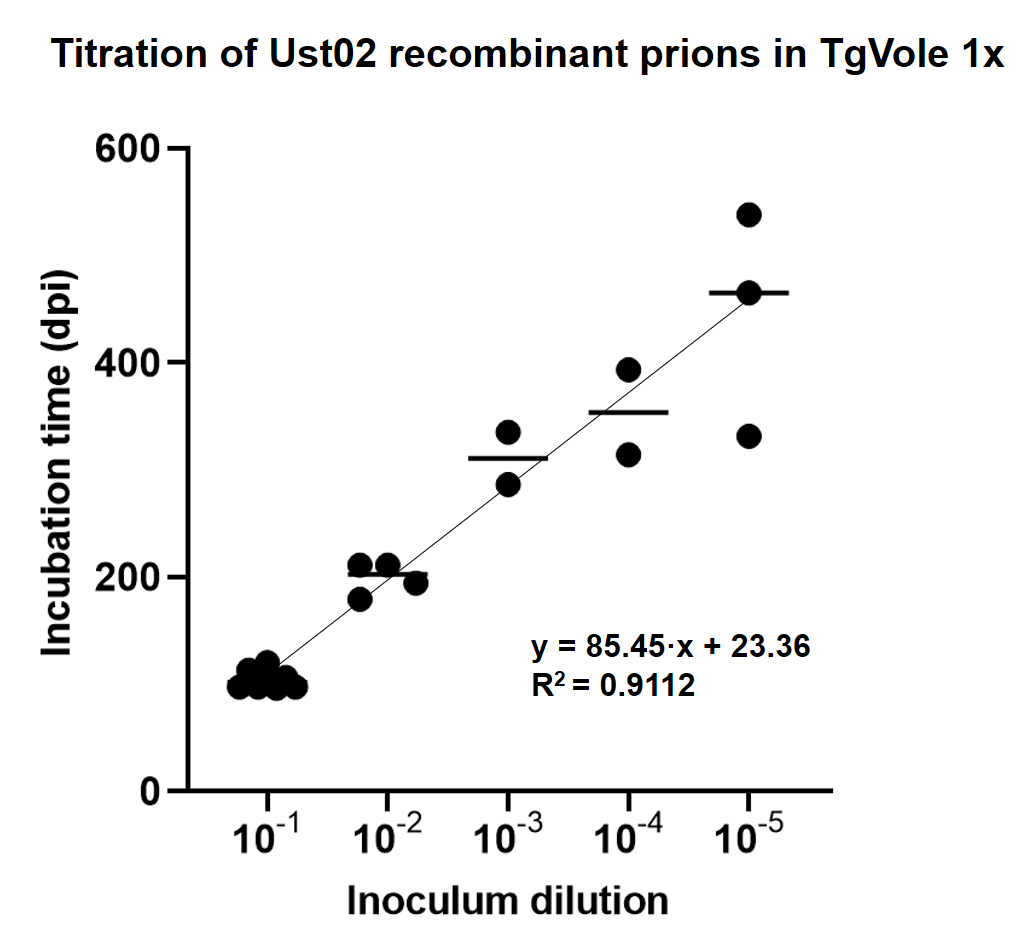


**Supplementary figure 7. Graphical representation of the results from the titration of Ust02 recombinant prions in TgVole 1x animals.** Results from the intracerebral inoculation of serially diluted Ust02 prions in TgVole 1x animals were plotted as a function of the incubation time, in order to facilitate extrapolation of Ust02 prion titer of a preparation based on the incubation period in TgVole 1x animals. Incubation periods of all PrP^Sc^ positive animals from each dilution and the mean of each group are depicted, as well as the linear regression line, along with its corresponding equation.

Therefore, we assume a misfolding or conversion yield of 100% and a total rec-PrP concentration at the substrate of 0.1 µg/µl, according to our precipitation experiments [1]. Taking into account that 20 µl of a 1:10 dilution of the preparation were inoculated (containing 0.2 µg of misfolded PrP) the LD50 is of 10^-4.92^ upon intracerebral inoculation. Adjusted for the misfolded PrP amount assuming 100% conversion, specific infectivity of Ust02 in TgVole is of 4.16·10^5^ LD50/µg of misfolded PrP, higher than another recombinant bank vole prion strain reported previously [1]. Assuming that the mean amount of PrP^Sc^ in brain-derived prions is similar to that found in hamster, we can estimate and compare specific infectivity as LD50 per µg of PrP^Sc^ also for CWD-Vole prion strain [2] which has been inoculated in the same TgVole model (unpublished results).

In our hands, and using the Spearman-Karber method [7], CWD-Vole in TgVole showed a LD50 of 10^-5.125^ upon intracerebral inoculation of 20 µl of 1% (w/V) brain homogenate. Thus, specific infectivity is of 6.6·10^8^ LD50/g of brain, close to that reported for another isolate of the same inoculum in bank voles, of 10^8.4^ LD50/g of brain [2]. Assuming that misfolded PrP amounts in TgVole at terminal stage are similar to those reported for 263K infected hamsters [6], of approximately 20 µg/g of brain, specific infectivity of CWD-Vole in TgVole would be of 3.34·10^7^ LD50/µg of misfolded PrP, approximately 80-fold higher than Ust02. The most relevant numbers have been included in table 3 to facilitate comparison.

**Supplementary table 2. Specific infectivity calculations for Ust02 recombinant prions compared to CWD-vole brain-derived prions**


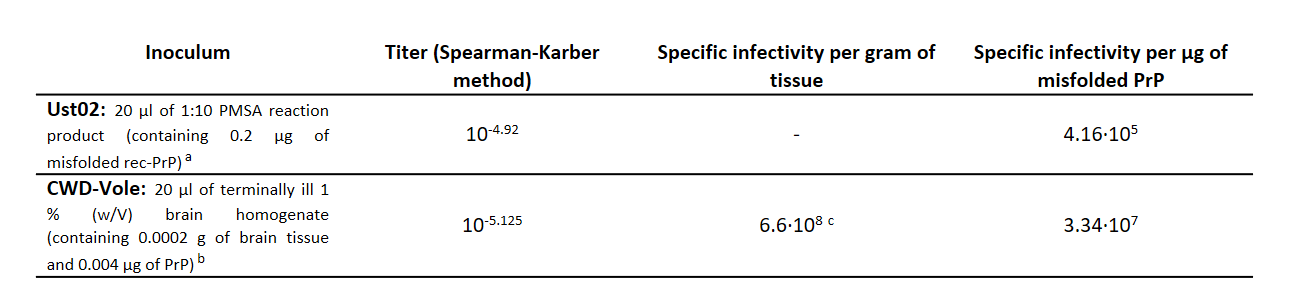


^a^ Assuming a conversion yield of 100% based on precipitation experiments (1).

^b^ Based on the misfolded PrP amount quantified in terminally ill hamsters inoculated with 263K prions (24).

^c^ Comparable to that reported for the same inoculum in bank voles (10^8.4^ LD50/g) (20).


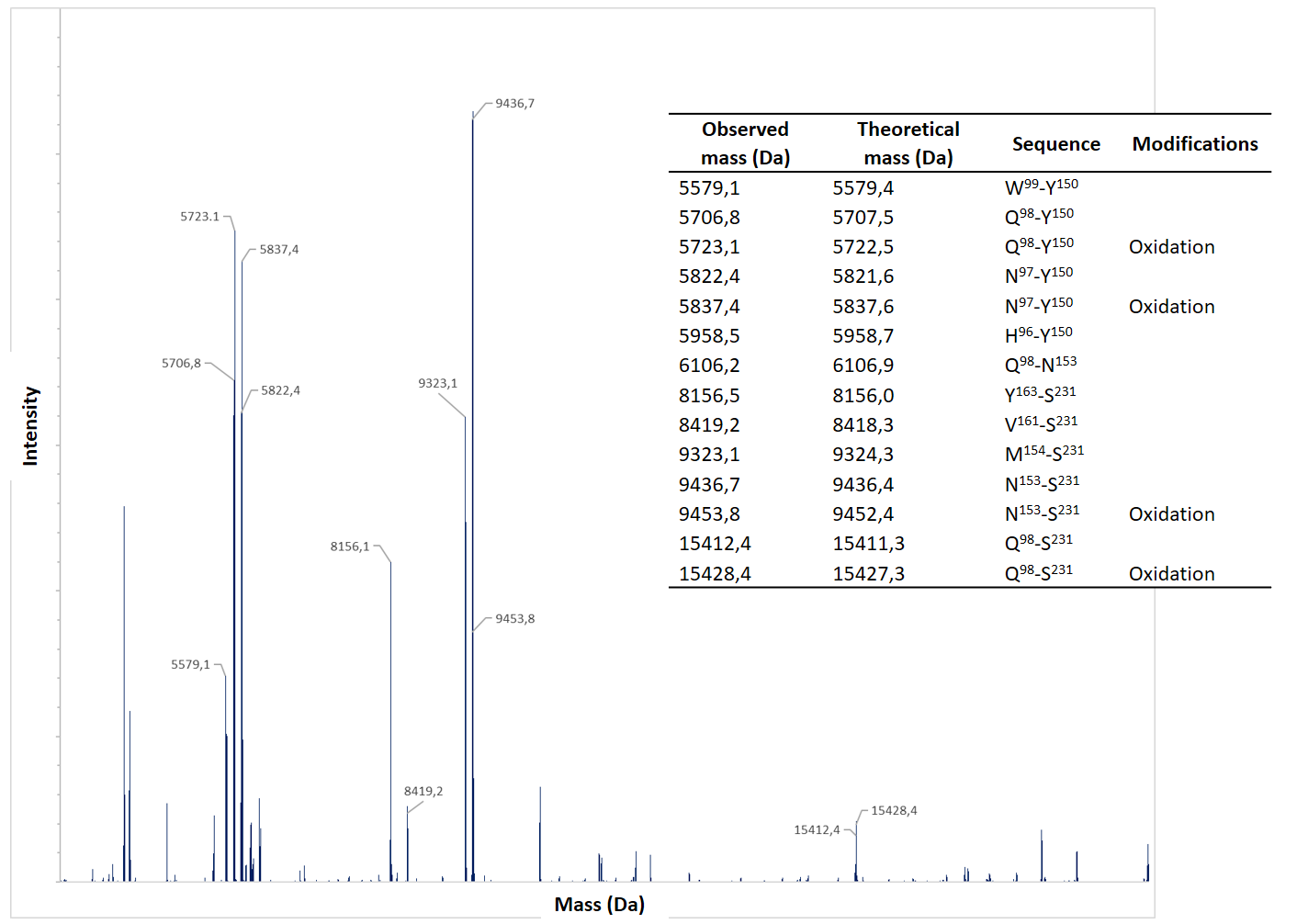


**Supplementary figure 8. Mass spectrometry-based analysis of the proteolytic fragments of the recombinant prion Ust02 after proteinase K digestion.** Ust02 PMSA product was digested with proteinase K (25 µg/ml at 42 ˚C for 1h), precipitated by centrifugation and analyzed in a mass spectrometer in order to determine the amino acid sequence of the PK-resistant fragments observed in acrylamide gels after the same digestion and total protein staining. The mass spectrometry analysis successfully identified the main fragments. The upper band, approximately 16 kDa in size according to electrophoresis was found to encompass residues 98 to 231. The central band, the most intense one and approximately 10 kDa in size, ranged from residues 153 or 154 to 231. Additionally, the fragment immediately below the central band contained residues from 161/163 to 231. Apart from these large fragments, low molecular weight fragments were also identified, matching the signal observed in the gels around 5 kDa. These fragments corresponded to residues 97/98-150/152/153, representing the N-terminal part of the ~10 kDa fragment.


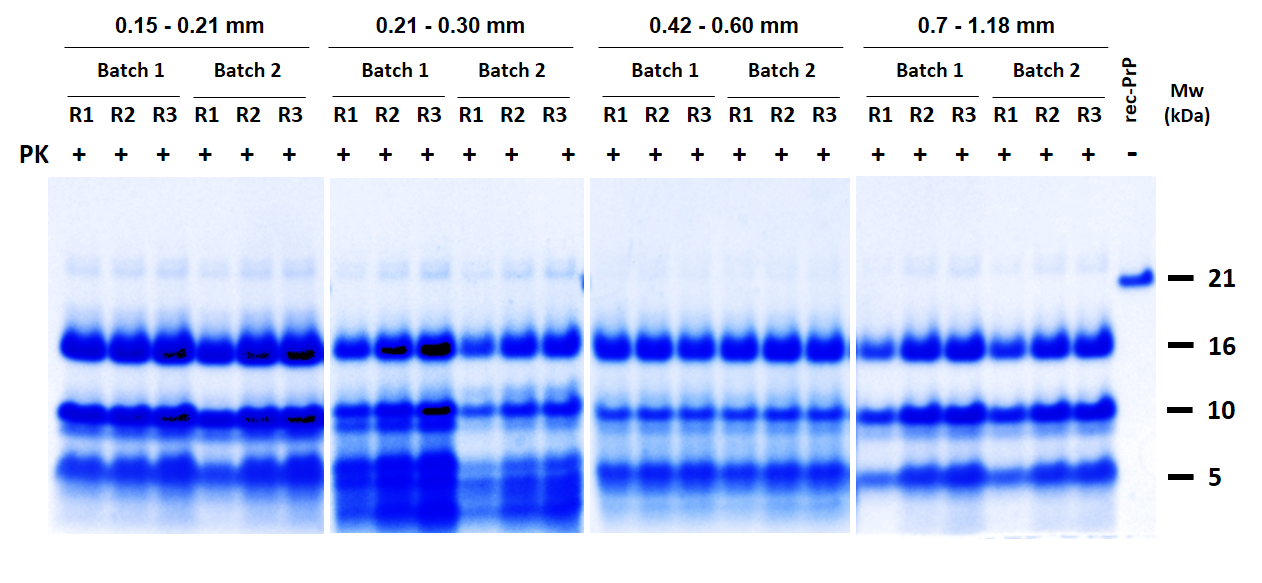


**Supplementary figure 9. Evaluation of the effect of glass bead size on the efficiency of spontaneous misfolding of recombinant PrP by PMSA.** The influence of four different glass bead sizes (0.15-0.21 mm, 0.21-0.30 mm, 0.42-0.60 mm and 0.7-1.18 mm diameter) on spontaneous misfolding was assessed. Two different batches of each bead type were used, with a comparable volume of beads added to each tube (ranging from approximately 50π mm^2^ with the largest beads to 100π mm^2^ for the smallest ones). Three consecutive 24 h PMSA rounds (R1 to R3) were conducted for each bead type, employing 1:10 dilutions from the previous round. The progress of spontaneous misfolding was monitored through proteinase K digestion, electrophoresis, and subsequent total protein staining. A representative experiment of the four replicates performed for each bead type is shown. The results indicate that irrespective of the bead size, rec-PrP^res^ was detected in all cases starting from the first PMSA round. This suggests that the efficiency of spontaneous misfolding is not dependent on bead size, but rather on the availability of a minimum glass surface area.


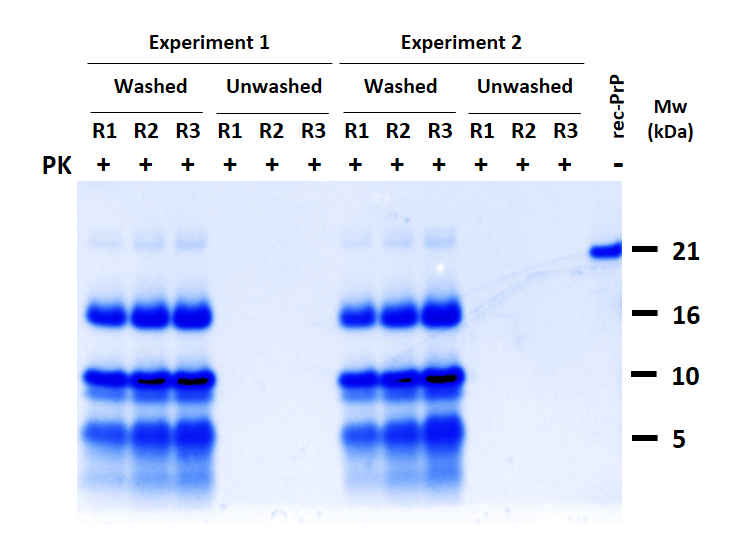

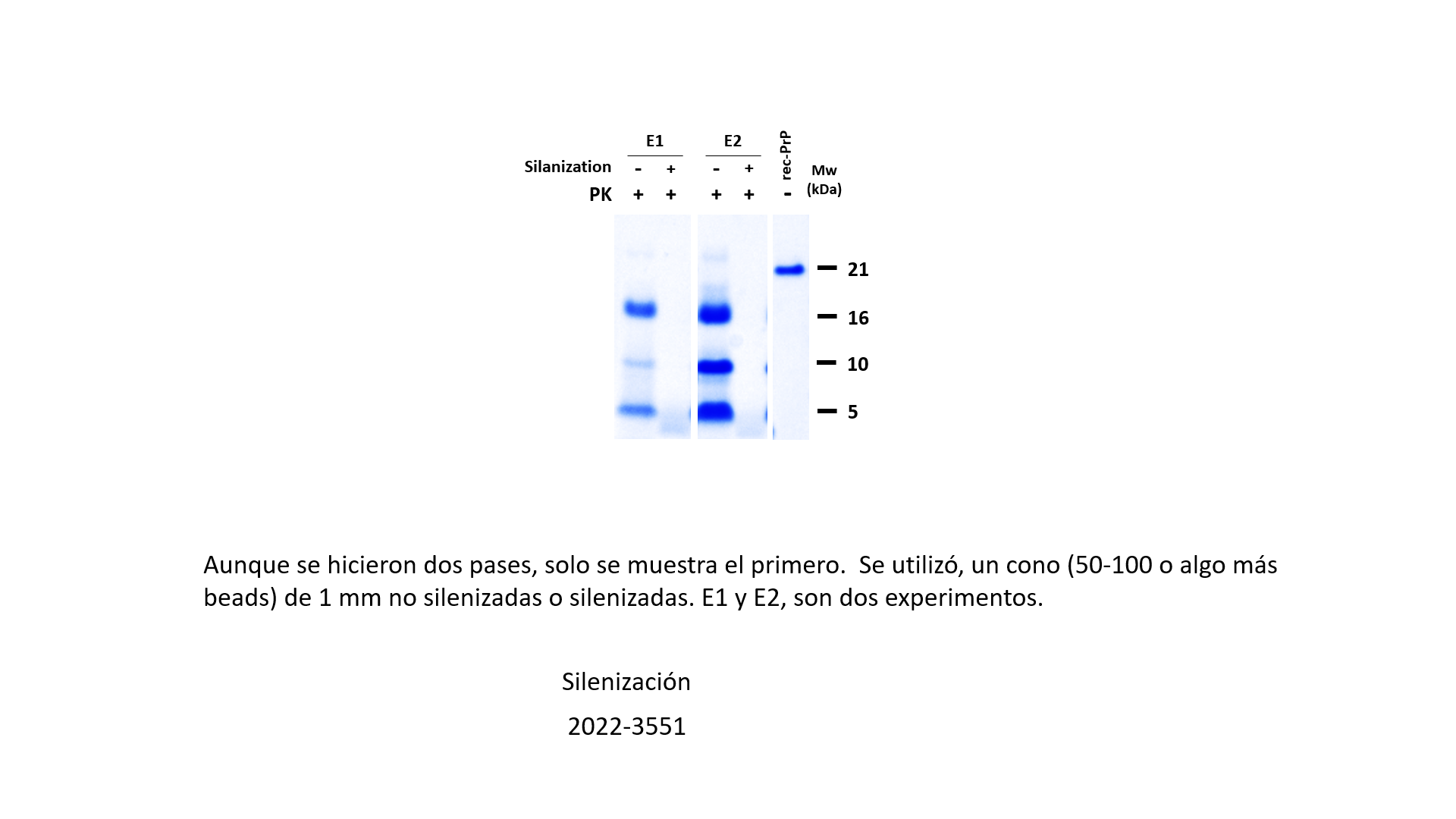


**B**

**A**

**Supplementary figure 10. Evaluation of the significance of a pristine and accessible glass surface on promoting spontaneous rec-PrP misfolding in PMSA. A) Effect of using acid-washed versus non acid-washed glass beads in promoting spontaneous misfolding of recombinant PrP by PMSA.** To evaluate the influence of glass surface quality on spontaneous misfolding, two types of glass beads were used: acid-washed glass beads (as previously employed) and non-acid-washed glass beads (with surface impurities due to the absence of acid washing during fabrication), both of the same size and quantity. Two independent experiments were performed, wherein the substrates supplemented with each type of beads were subjected to three consecutive 24 h PMSA rounds denoted as R1 to R3. For each round, 1:10 dilutions of the PMSA product from the previous round were used. Following proteinase K digestion (PK), electrophoresis, and total protein staining, rec-PrP^res^ could only be detected from the first round when acid-washed beads were employed, indicating the necessity of a clean glass surface to promote spontaneous misfolding in PMSA. **B) Effect of siliconization of the surface of glass beads on the spontaneous misfolding efficiency.** To further evaluate the importance of available glass surface, acid-washed beads were treated with dimethyldichlorosilane, creating a coat of silane on the surface of the glass beads. The silanized beads and untreated beads (used as a positive control) were added to the PMSA substrate. Two independent experiments (E1 and E2) were performed, subjecting tubes containing siliconized and non-siliconized beads to a single 24 h PMSA round. Following PK digestion, electrophoresis, and total protein staining, rec-PrP^res^ was exclusively detected in the tubes containing non-siliconized beads, demonstrating that the availability of a glass surface is crucial for highly efficient spontaneous misfolding in PMSA. MW: Molecular weight marker. Rec-PrP: undigested recombinant bank vole PrP containing PMSA substrate.


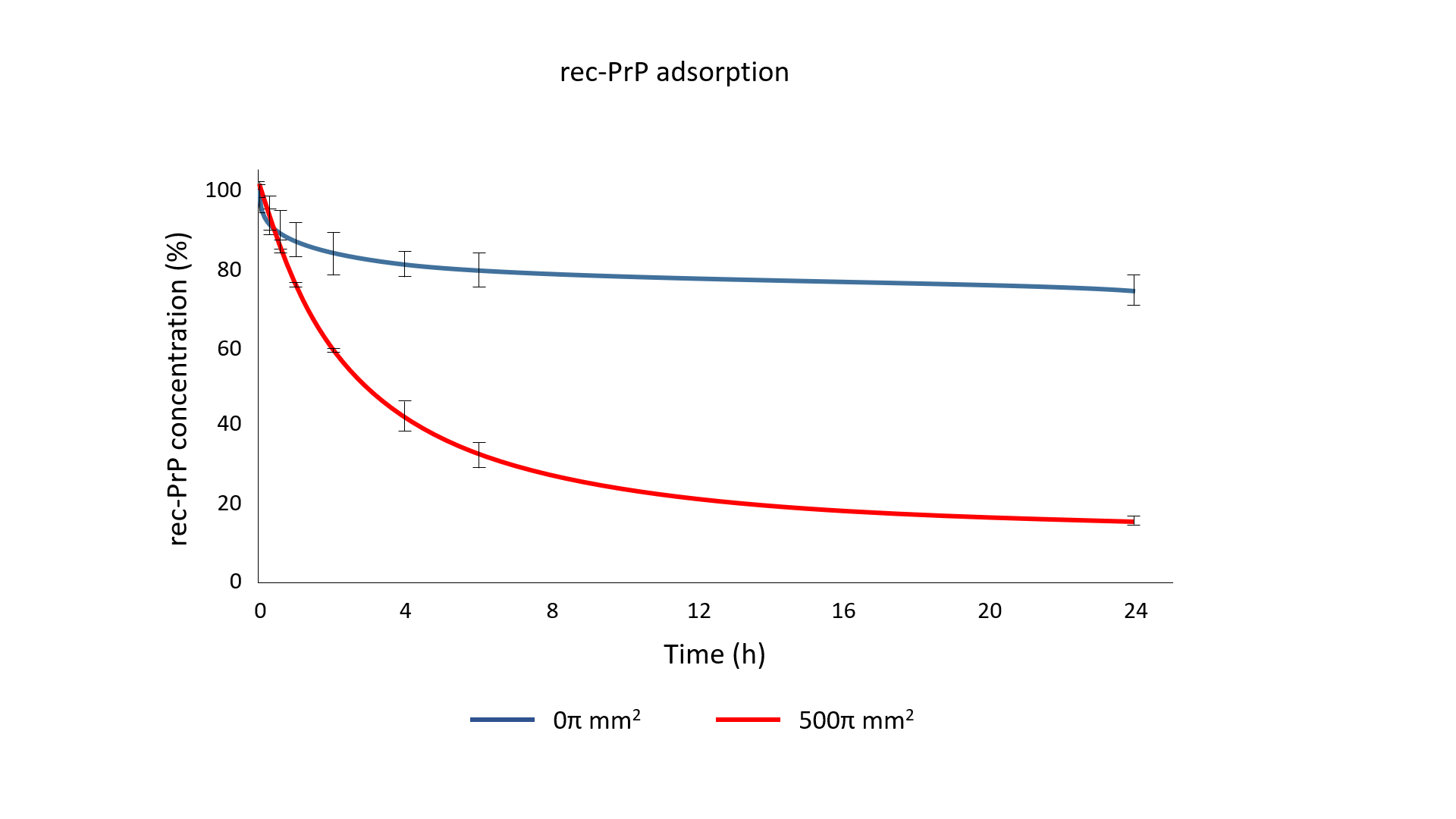


**Supplementary figure 11. Assessment of rec-PrP adsorption from PMSA substrate to glass beads over time.** To investigate the potential binding of rec-PrP from the PMSA substrate to the glass surface of the beads used in spontaneous PMSA reactions, the PMSA substrate was incubated for up to 24 h with beads equivalent to a surface area of approximately 500π mm^2^, while gentle shaking. As a control, a tube without beads underwent the same conditions. Aliquots from the supernatant of each tube were collected at 15 min, 30 min, 1 h, 2 h, 4h, 6 h, and 24 h, after letting beads to sediment, and the concentration of rec-PrP was estimated through electrophoresis and total protein staining. A densitometric analysis was performed to measure the soluble rec-PrP concentration in the supernatant over time. The results revealed that more than 50 % of the rec-PrP from the substrate becomes adsorbed to glass beads within 4 h of incubation. This adsorption gradually decreased with only 20 % of the initial concentration remaining after 24 h.


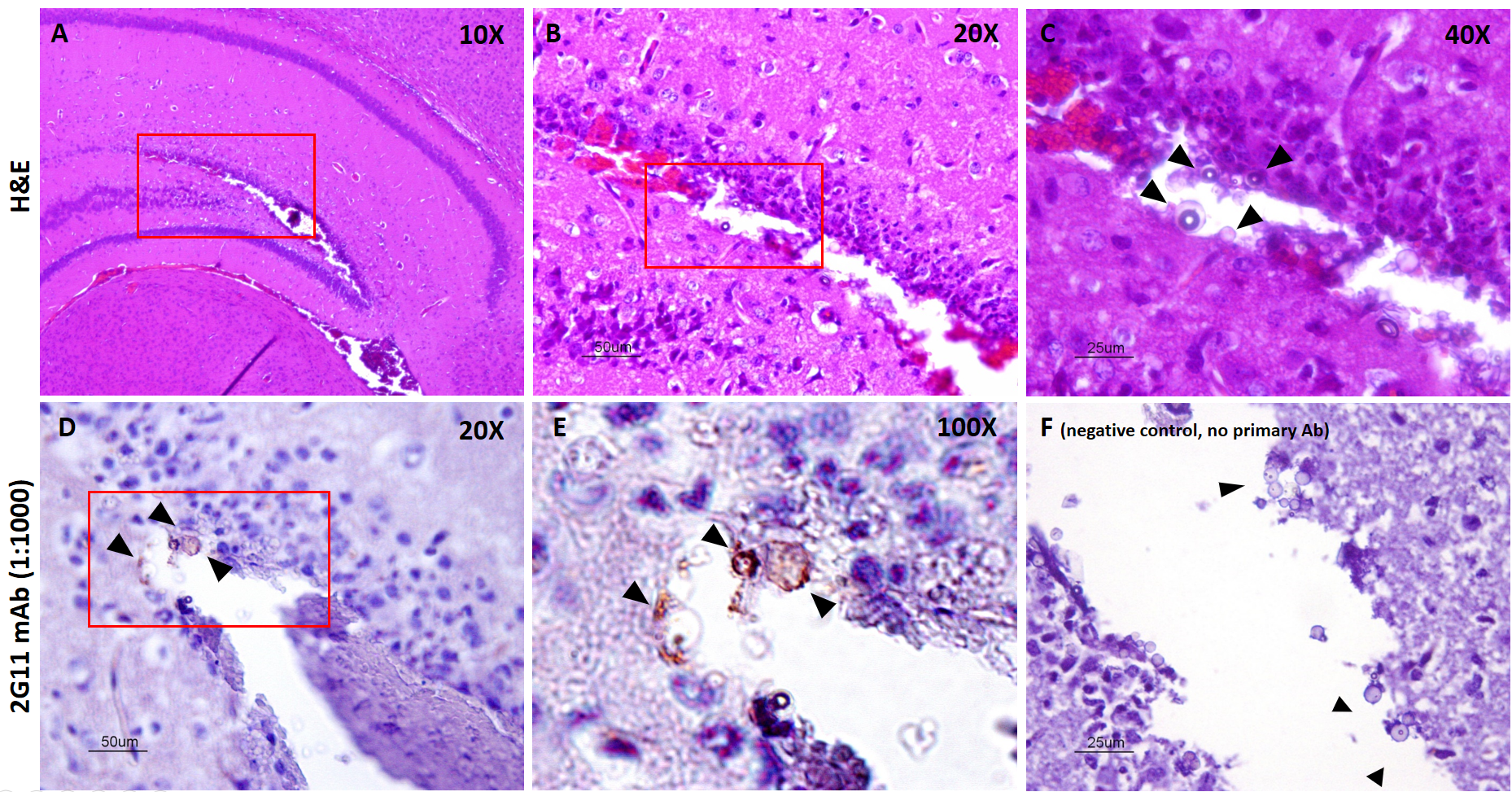


**Supplementary figure 12.** **Histopathological and immunohistochemical analysis of mouse brain 72 h after inoculation with recombinant prion-loaded glass spheres.** Ust02 recombinant prion-coated glass sphere solution was inoculated intracerebrally in C57BL6 mice to assess whether recombinant prions adsorbed to glass spheres were detectable in the injection site after 72 h. The aim was to determine if these prion-coated glass beads could serve as an inoculum. Following the injection, localized hemorrhagic and damaged tissue was observed in the hippocampus (2.2 points approximately caudolateral to the Bregma). Hematoxylin-eosin staining clearly revealed the presence of glass spheres (9-13 µm diameter) at magnifications of **A)** 10x, **B)** 20x, and **C)** 40x. Further immunohistochemical analysis was conducted using 2G11 anti-PrP antibody (1:1000) in the same area **D)** 20x and **E)** 100x magnifications. This analysis confirmed that the glass spheres were indeed coated with PrP^res^, as indicated by positive labeling. In the control condition **F)**, were the primary antibody was omitted, no labeling was observed, confirming the specificity of the immunohistochemical staining.

**Specific infectivity of recombinant prion-coated glass spheres *in vivo*, Lethal dose 50 (LD_50_) calculation for Ust02 recombinant prion adsorbed to glass spheres**

In order to calculate the specific infectivity of Ust02 prions adsorbed to glass spheres, a 24 h PMSA round was performed. The PMSA substrate was supplemented with glass spheres measuring 9-13 µm diameter and seeded with Ust02 recombinant prions at a 1:10 dilution. After de PMSA reaction, the supernatant was discarded and the sedimented spheres were thoroughly washed multiple times with PBS. Following the final wash, the spheres were, resuspended in the same volume as the initial substrate. The resulting suspension, containing Ust02 prion-loaded spheres, was subjected to serial dilutions ranging from 10^-1^ to 10^-6^ in PBS. Subsequently, all the serial dilutions were further diluted 1:10 in PBS, as commonly done with liquid inocula to obtain 1% solutions. Each dilution was then intracerebrally inoculated into TgVole 1x animals, with 20 µl administered to each animal following the same procedure used for standard liquid inocula.

To calculate the specific infectivity of Ust02 prions adsorbed to glass spheres, we applied similar considerations as before. The conversion yield for the PMSA reaction in generating recombinant prion was assumed to be close to 100 %, based on precipitation experiments and quantification of total PrP in the supernatant and pellet. This lead to the same calculation of misfolded PrP amount inoculated of 0.2 µg. As previously calculated for Ust02 prions in solution, the LD50 of sphere-adsorbed Ust02 prions was found to be 10^-3.19^ upon intracerebral inoculation. Adjusting for the 100 % conversion yield and the misfolded PrP amount inoculated, the specific infectivity in this case was determined to be 3.23·10^3^ LD50/µg of misfolded PrP. This value is approximately 130 times lower than that of the liquid Ust02 inoculum.

**Supplementary table 3. Results from intracerebral inoculation of serially diluted recombinant bank vole Ust02 prions adsorbed in glass spheres in TgVole (1x) mice to calculate specific infectivity.**


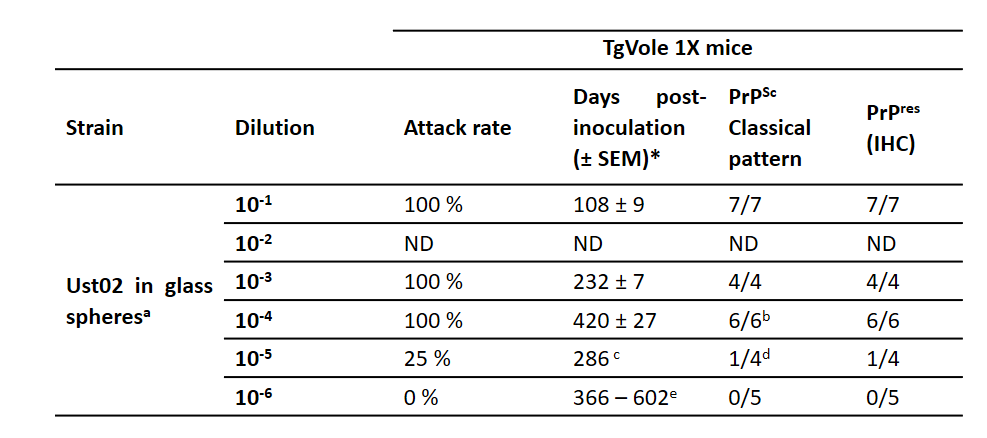


^a^ Inoculum was prepared diluting the PMSA product 1:10 in PBS, which was afterwards serially diluted 1:10 up to dilution 10^-6^ of the PMSA product. 20 µl of the diluted inocula were inoculated intracerebrally in each TgVole mouse.

^b^ 1 animal from this group died with intercurrent disease and was excluded from the study.

^C^ The only animal that showed PrP^Sc^ and PrP^res^ from the group was euthanized due to clinical signs compatible with a prion disease. The other three animals from the group were euthanized at 493, 556 and 603 dpi and were all negative for PrP^Sc^ and PrP^res^.

^d^ 1 animal from this group died with intercurrent disease and was excluded from the study.

^e^ Despite apparent neurological signs in some animals euthanized at 366, 438 and 462 dpi, they showed no PrP^Sc^ or PrP^res^. The rest of the animals from the group were euthanized at 602 dpi and were also negative for PrP^Sc^ and PrP^res^. Survival period range of the three animals from the group is reported in absence of PrP^Sc^ or PrP^res^ positive individuals within the experimental group.

* Incubation periods were calculated taking into account only those animals from each group that were positive for PrP^Sc^ (classical three-banded pattern) by WB or for PrP^res^ by IHC.

ND: Not determined.


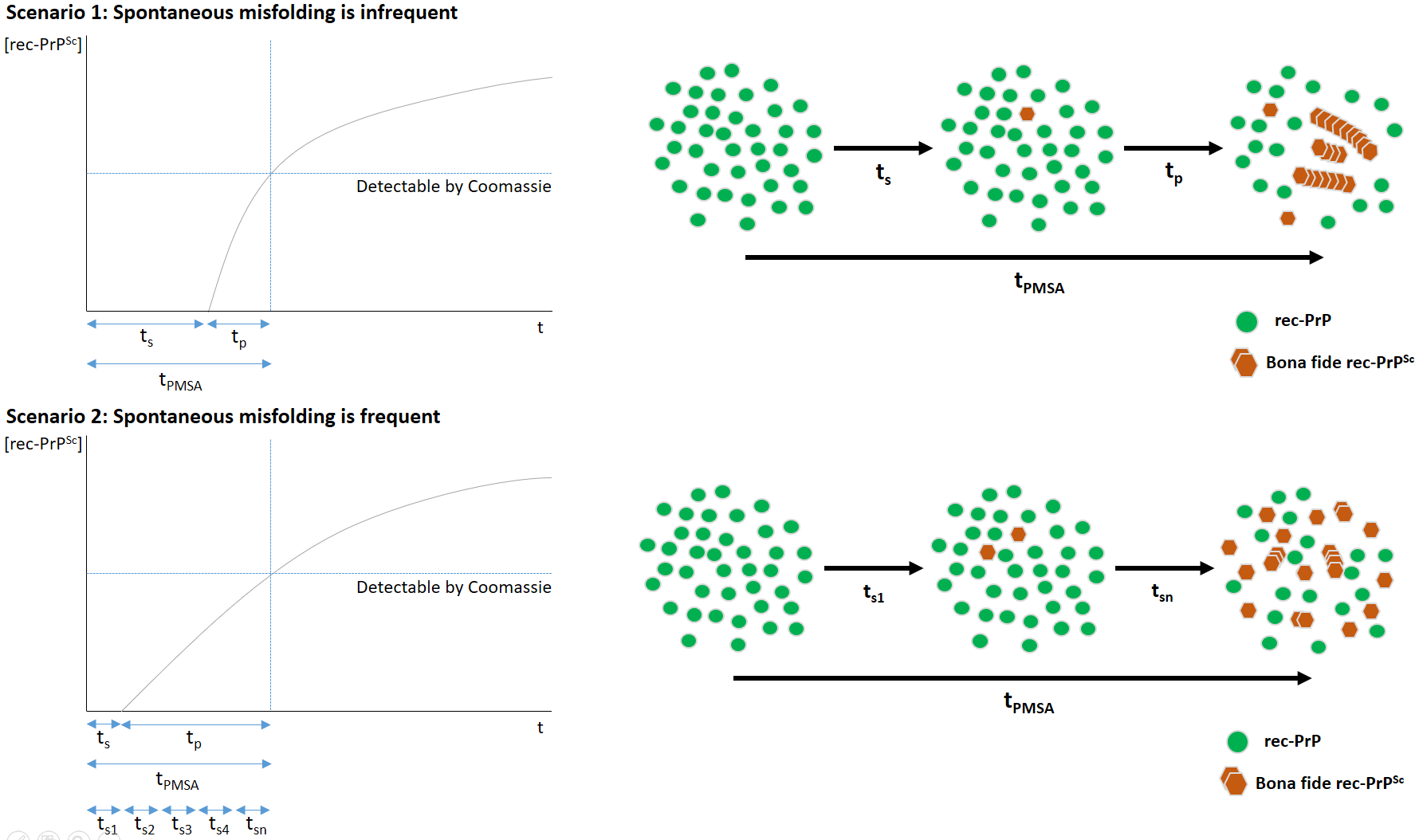


**B**

**A**


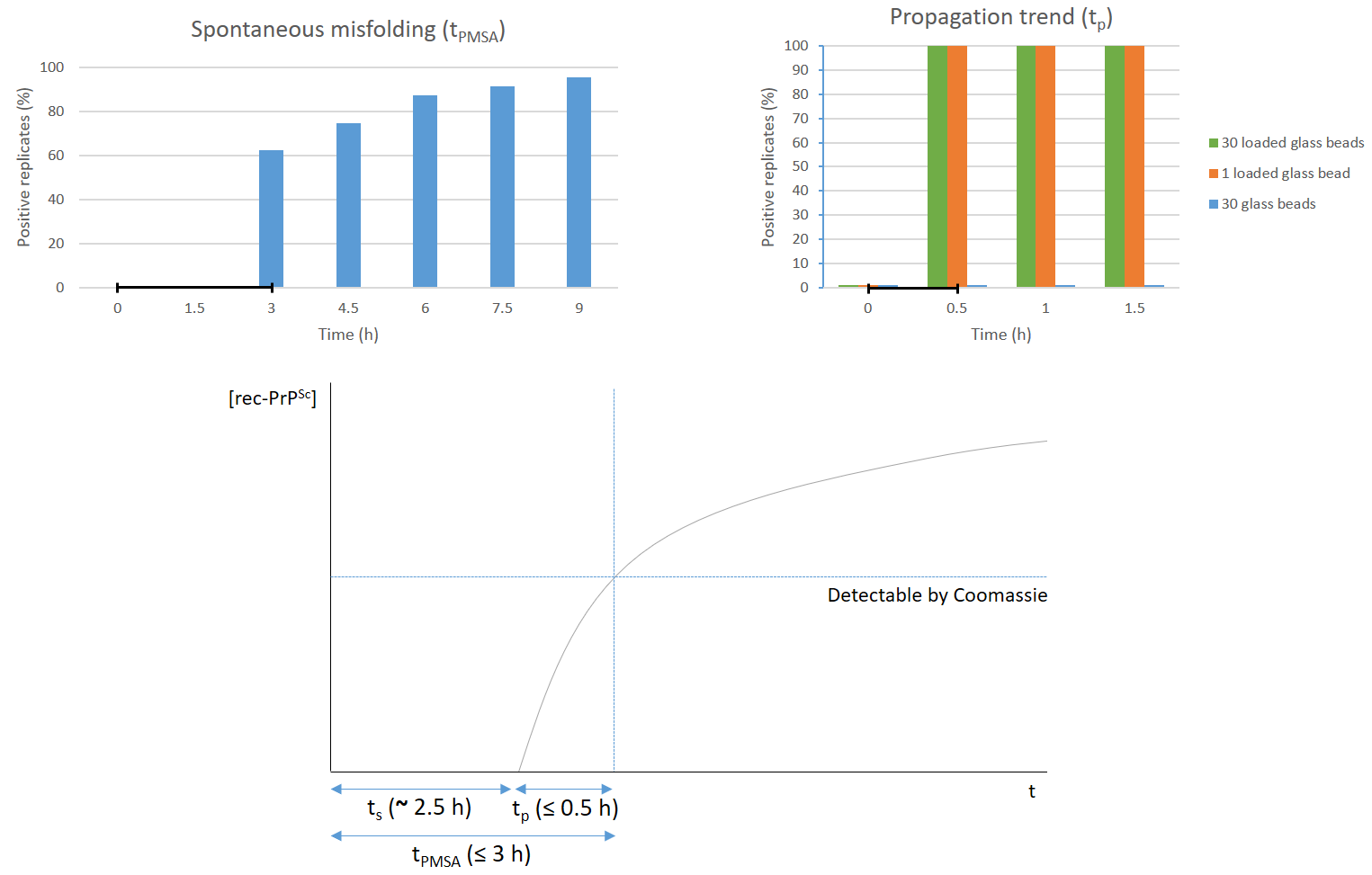


**Supplementary figure 13. Evaluation of the kinetics and occurrence rate of spontaneous prion misfolding in PMSA. A) Graphical representation of recombinant prion misfolding and propagation phenomena along a single PMSA round in terms of rec-PrP^res^ concentration and reaction time**. Two possible scenarios for the occurrence of spontaneous rec-PrP misfolding and subsequent propagation are depicted, leading to detectable rec-PrP^res^ levels by proteinase K digestion, electrophoresis and total protein staining. In the first scenario, rec-PrP^res^ concentration increases gradually during the PMSA reaction, assuming that spontaneous rec-PrP misfolding is an infrequent and slow event. This is represented by the parameter **t_s_**, which signifies the time required for the formation of a single propagation nucleus or a *bona fide* misfolded rec-PrP particle capable of rapid propagation. The subsequent propagation time is represented by the parameter **t_p_** or propagation time, and the total reaction time until detectable amounts of rec-PrP^res^ are obtained, is denoted as **t_PMSA_** (also defined as **t_s_ + t_p_**). In this scenario, if spontaneous misfolding is infrequent, ts would be long until the formation of the first propagation nucleus or spontaneously misfolded rec-PrP^res^, followed by a rapid propagation phase during which rec-PrP^res^ concentration increases quickly until detectable levels are reached. On the contrary, the scenario 2 depicts the opposite possibility, where spontaneous rec-PrP^res^ misfolding is frequent and occurs rapidly on several occasions during the initial phases of PMSA. This is followed by subsequent propagation until detectable amounts are reached. **B) Results from the experiment performed to assess misfolding and propagation times** were obtained considering that one prion-loaded bead represents the minimum amount of spontaneously misfolded rec-PrP^res^ that can be retrieved from or added to a PMSA reaction. To calculate **t_PMSA_**, an unseeded PMSA was conducted with 30 glass beads of 1 mm diameter in quadruplicate. Supernatant was collected every 90 min to evaluate presence of rec-PrP^res^ through proteinase K digestion, electrophoresis and total protein staining. Results showed detectable rec-PrP^res^ in 60 % of the replicate tubes in 3 h of reaction, serving as a model system for **t_PMSA_** calculation. Parallel to the unseeded reaction, two seeded PMSA reactions were performed to calculate the propagation time (**tp**). One reaction contained a single prion-loaded glass bead, simulating scenario 1, while the other had 30 prion-loaded glass beads, simulating scenario 2. Additionally, an unseeded reaction with 30 non-prion-loaded glass beads served as a negative control for spontaneous misfolding or cross-contamination. Supernatant from each type of PMSA reaction was retrieved every 30 min to evaluate rec-PrP^res^ formation by PK digestion, electrophoresis and total protein staining. The results showed that **t_p_** was similar for both 1 and 30 prion-loaded glass beads, with detectable rec-PrP^res^ in all replicates within 30 min, indicating that prion propagation in PMSA is an extraordinarily rapid and efficient event. In the light of these results, and considering that **t_PMSA_** in a spontaneous reaction is of 3 h and that the propagation time or **t_p_** either from a single bead, or from the same amount used in the unseeded reaction, is of 30 min; **t_s_** is calculated to be of approximately 2.5 h. Collectively these results suggest that spontaneous rec-PrP misfolding in PMSA is likely an infrequent and relatively slow event, followed by rapid and highly efficient propagation of the initial seeds formed, as illustrated in the graphical representation at the bottom of the figure.

**Descriptive video of the PMSA procedure developed for the generation of spontaneous protein misfolding**

This animation illustrating how the spontaneous misfolding event occurs was planned and done in 2020, prior to the publication of the first high-resolution structure [8]. For that reason, the video shows a PrP^Sc^ structure based on the 4 rung β-solenoid model, proposed before as the potential three dimensional structure of prions [9]. Please note that the protein misfolding depicted in the video is fictional and is only intended to illustrate one of the possible interpretations of the process. At the end of the video, a real image is shown featuring a glass bead completely covered by fibrillar aggregates.

**References**

1. Erana H, Charco JM, Di Bari MA, Diaz-Dominguez CM, Lopez-Moreno R, Vidal E, Gonzalez-Miranda E, Perez-Castro MA, Garcia-Martinez S, Bravo S, Fernandez-Borges N, Geijo M, D'Agostino C, Garrido J, Bian J, Konig A, Uluca-Yazgi B, Sabate R, Khaychuk V, Vanni I, Telling GC, Heise H, Nonno R, Requena JR, Castilla J (2019) Development of a new largely scalable in vitro prion propagation method for the production of infectious recombinant prions for high resolution structural studies. PLoS Pathog 15:e1008117. <https://doi.org/10.1371/journal.ppat.1008117>

2. Di Bari MA, Nonno R, Castilla J, D'Agostino C, Pirisinu L, Riccardi G, Conte M, Richt J, Kunkle R, Langeveld J, Vaccari G, Agrimi U (2013) Chronic wasting disease in bank voles: characterisation of the shortest incubation time model for prion diseases. PLoS Pathog 9:e1003219. <https://doi.org/10.1371/journal.ppat.1003219>

3. Klingeborn M, Race B, Meade-White KD, Chesebro B (2011) Lower specific infectivity of protease-resistant prion protein generated in cell-free reactions. Proc Natl Acad Sci U S A 108:E1244-1253. <https://doi.org/10.1073/pnas.1111255108>

4. Deleault NR, Walsh DJ, Piro JR, Wang F, Wang X, Ma J, Rees JR, Supattapone S (2012) Cofactor molecules maintain infectious conformation and restrict strain properties in purified prions. Proc Natl Acad Sci U S A 109:E1938-1946. <https://doi.org/10.1073/pnas.1206999109>

5. Kim C, Haldiman T, Cohen Y, Chen W, Blevins J, Sy MS, Cohen M, Safar JG (2011) Protease-sensitive conformers in broad spectrum of distinct PrPSc structures in sporadic Creutzfeldt-Jakob disease are indicator of progression rate. PLoS Pathog 7:e1002242. <https://doi.org/10.1371/journal.ppat.1002242>

6. Onisko BC, Silva CJ, Dynin I, Erickson M, Vensel WH, Hnasko R, Requena JR, Carter JM (2007) Sensitive, preclinical detection of prions in brain by nanospray liquid chromatography/tandem mass spectrometry. Rapid Commun Mass Spectrom 21:4023-4026. <https://doi.org/10.1002/rcm.3310>

7. Ramakrishnan MA (2016) Determination of 50% endpoint titer using a simple formula. World J Virol 5:85-86. <https://doi.org/10.5501/wjv.v5.i2.85>

8. Kraus A, Hoyt F, Schwartz CL, Hansen B, Artikis E, Hughson AG, Raymond GJ, Race B, Baron GS, Caughey B (2021) High-resolution structure and strain comparison of infectious mammalian prions. Molecular Cell. <https://doi.org/10.1016/j.molcel.2021.08.011> PMID - 34433091

9. Spagnolli G, Rigoli M, Orioli S, Sevillano AM, Faccioli P, Wille H, Biasini E, Requena JR (2019) Full atomistic model of prion structure and conversion. PLoS Pathog 15:e1007864. <https://doi.org/10.1371/journal.ppat.1007864>
